# Initiation of Pregabalin vs Gabapentin and Development of Heart Failure

**DOI:** 10.1001/jamanetworkopen.2025.24451

**Published:** 2025-08-01

**Authors:** Elizabeth E. Park, Laura L. Daniel, Alyson L. Dickson, Meghan Corriere, Puran Nepal, Kathi Hall, W. Dale Plummer, William D. Dupont, Katherine T. Murray, C. Michael Stein, Wayne A. Ray, Cecilia P. Chung

**Affiliations:** 1 Department of Medicine, Columbia University Irving Medical Center, New York, New York; 2 Department of Medicine, University of Miami and Miami VA Healthcare System, Miami, Florida; 3 Department of Medicine, Vanderbilt University Medical Center, Nashville, Tennessee; 4 Department of Pharmacology, Vanderbilt University Medical Center, Nashville, Tennessee; 5 Department of Biostatistics, Vanderbilt University Medical Center, Nashville, Tennessee; 6 Department of Health Policy, Vanderbilt University Medical Center, Nashville, Tennessee; 7 Vertex Pharmaceuticals, Boston, Massachusetts

## Abstract

**Importance:**

Both pregabalin and gabapentin are common nonopioid medications used to treat chronic pain, which affects up to 30% of patients. Because pregabalin has greater potency than gabapentin in binding to the α2δ subunit of the L-type calcium channel, pregabalin may be associated with an increased risk for heart failure (HF).

**Objective:**

To compare incident hospitalizations and emergency department (ED) visits for HF among new users of pregabalin vs gabapentin among Medicare beneficiaries with noncancer chronic pain.

**Design, Setting, and Participants:**

This retrospective cohort study included data collected between January 1, 2015, and December 21, 2018, for Medicare beneficiaries aged 65 to 89 years with chronic noncancer pain and without a history of HF and terminal illnesses. Data were analyzed from March 21 to December 2, 2024.

**Exposure:**

New use of pregabalin or gabapentin.

**Main Outcomes and Measures:**

The primary outcome was hospital admission or ED visit with a primary discharge diagnosis of HF. Secondary outcomes were incidence of outpatient HF diagnosis and all-cause mortality. Adjusted hazard ratios (AHRs) were calculated using inverse probability of treatment weighting propensity score adjustment accounting for 231 covariates, including demographic, clinical, and health care utilization variables and medication use.

**Results:**

The cohort included 246 237 Medicare beneficiaries, of whom 18 622 (7.6%) were new users of pregabalin and 227 615 (92.4%) were new users of gabapentin; the cohort was predominantly female (66.8%), with a median age of 73 years (IQR, 69-78 years). During 114 113 person-years of follow-up, 1470 patients had a hospital admission or ED visit for HF. The rate of HF was 18.2 (95% CI, 15.3-21.6) per 1000 person-years for pregabalin and 12.5 (95% CI, 11.9-13.2) per 1000 person-years for gabapentin (AHR, 1.48 [95% CI, 1.19-1.77]). When restricted to patients with a history of cardiovascular disease, pregabalin was associated with an elevated HF risk compared with gabapentin (AHR, 1.85 [95% CI, 1.38-2.47]). Increased risk of outpatient HF incidence was also noted among patients receiving pregabalin compared with those receiving gabapentin (AHR, 1.27 [95% CI, 1.02-1.58]). All-cause mortality was not significantly different between groups (AHR, 1.26 [95% CI, 0.95-1.76]).

**Conclusions and Relevance:**

In this retrospective cohort study of Medicare beneficiaries with chronic noncancer pain, incident HF was increased in new users of pregabalin compared with new users of gabapentin. These findings should be considered, especially in patients with a history of cardiovascular disease.

## Key Points

**Question:**

Is pregabalin associated with higher incidence of heart failure (HF) compared with gabapentin?

**Findings:**

In this cohort study of 246 237 Medicare beneficiaries aged 65 to 89 years with noncancer chronic pain, pregabalin was associated with a higher incidence of HF compared with gabapentin.

**Meaning:**

The findings suggest that pregabalin should be prescribed with caution in older patients with noncancer chronic pain.

## Teaser

This cohort study assesses whether pregabalin is associated with higher incidence of heart failure compared with gabapentin in older populations with noncancer chronic pain.

## Introduction

Nonopioid medications, such as pregabalin and gabapentin, are gabapentinoids (γ-aminobutyric acid analogues) widely prescribed for chronic pain disorders.^1^ These medications are preferred over opioids for treatment of chronic noncancer pain because of the increased risks of addiction, overdose, and mortality associated with opioid use.^2^ Reflecting this trend, in the US, the estimated proportion of the population reporting the use of gabapentinoid medications increased steadily from 1.2% (95% CI, 1.0%-1.4%) in 2002 to 4.0% (95% CI, 3.6%-4.4%) in 2015 and 4.7% (95% CI, 4.4%-5.1%) in 2021 (*P* < .001 for the 2002 to 2015 comparison; *P* < .01 for the 2015 to 2021 comparison).^3^ Nonopioid medications are specifically indicated for older adults (age >65 years) because they are among those at highest risk for opioid-related adverse effects.^4^

Both pregabalin and gabapentin bind specific subunits (α2δ-1 and α2δ-2) of P/Q-type and N-type neuronal voltage-gated calcium channels, decrease the release of neurotransmitters, and have antinociceptive effects.^5^ Cardiovascular adverse effects, such as peripheral edema^6^ and heart failure (HF),^7,8^ have been associated with both pregabalin and gabapentin potentially due to additional effects on α2δ subunits of L-type calcium channels located on arteries and ventricular cardiomyocytes. However, because of greater potency^4^ and receptor (α2δ-1) binding affinity^9,10^ of pregabalin compared with gabapentin, the risk for adverse events with pregabalin may be higher than that with gabapentin. Clinical findings have supported these concepts, as data from the French Pharmacovigilance Centers identified reports of HF occurring with pregabalin treatment but not with gabapentin.^6^

Based on currently available but limited evidence of increased risk of HF associated with pregabalin compared with gabapentin, the American Heart Association currently lists pregabalin, but not gabapentin, as a medication that may cause or exacerbate HF.^11^ The European Medicines Agency recommends caution when using pregabalin, but not gabapentin, in older patients with cardiovascular comorbidities due to the increased risk of HF.^12^

Despite these recommendations, only a few cohort studies^8,13,14,15,16^ have examined the comparative risk of HF between pregabalin and gabapentin. Most of these prior studies restricted their analyses to neurological indications for use (neuropathy or seizures),^8,16^ did not use a rigorous definition of HF,^8,13^ did not exclude those with a history of HF,^13,14^ or did not focus on older patients.^14^

Thus, the relationship between the incidence of HF and pregabalin use among patients at the highest risk of adverse reactions (ie, older patients with cardiovascular comorbidities) remains unclear. Our hypothesis was that pregabalin would be associated with a higher risk for HF in this population compared with gabapentin. To assess this association without a randomized clinical trial, we conducted a target trial emulation comparing HF incidence among Medicare beneficiaries prescribed pregabalin or gabapentin for a diverse range of chronic noncancer pain indications.

## Methods

### Data Source

This cohort study included a 20% sample of beneficiaries from the nationwide administrative claims Medicare database from January 1, 2015, to December 21, 2018. Medicare provides health care insurance for all US citizens aged 65 years or older. The Medicare claims data included the following parts: beneficiary summary file, which includes enrollment status and identifies deaths for beneficiaries; claims files for medical care services (pharmacy, hospital, outpatient, and nursing home); Part D event files; home health; durable medical equipment; outpatient standard analytic file (institutional outpatient); carrier file (noninstitutional outpatient); Medicare Provider Analysis and Review file (inpatient or skilled nursing facilities); and hospice file.^17,18,19,20^ The study was approved with a waiver of informed consent by the institutional review boards of Vanderbilt University Medical Center and the University of Miami because of the impracticality of obtaining consent from every Medicare patient. This study followed the Strengthening the Reporting of Observational Studies in Epidemiology (STROBE) reporting guideline.

### Design

This study emulated a hypothetical target trial,^21,22^ in which Medicare patients filled new prescriptions of pregabalin or gabapentin for noncancer pain (eTables 1 and 2 in Supplement 1). Target trial emulation uses observational data to mimic the design of a hypothetical randomized clinical trial, including all of its key components, to estimate causal effects while reducing common biases associated with observational data (eTable 1 in Supplement 1).

### Cohort Eligibility Criteria

The cohort consisted of Medicare beneficiaries aged 65 to 89 years and was restricted to those with Part A (hospitalization), Part B (outpatient medical care), and Part D (prescription drug) coverage. Part C enrollees, who had care through private insurance companies, were excluded from this study because of the possibility of missing claims data. To enter the cohort, participants must have had filled a new prescription (ie, no prescription in the prior 365 days) of pregabalin (study drug) or gabapentin (active comparator) during the study period. To be included in the study, patients must have had a diagnosis of chronic pain (eTable 3 in Supplement 1) in the year prior to being prescribed a study drug, continuous enrollment in Medicare (Part A, B, and D), and to ensure regular contact with medical care, at least 1 outpatient visit and 1 filled prescription. Participants were excluded if they had met the following conditions within 1 year prior to enrollment: a history of HF, terminal illnesses (severe illnesses including cancer, HIV infection, kidney failure, severe cardiorespiratory conditions, organ transplant, serious neuromuscular disorders, feeding problems, and other end-stage illness including spinal cord disorders and multiple sclerosis), a stay longer than 29 days at a long-term care facility (nursing home, other residential institution, or skilled nursing facility), a hospital stay exceeding 29 days (cumulative or continuous),^2,17^ hospitalization on the day of the prescription, or a hospice stay.

To determine whether the patient had a history of HF (exclusion criterion), we used *International Classification of Disease, Ninth Revision* (*ICD-9*) and *International Statistical Classification of Diseases and Related Health Problems, Tenth Revision* (*ICD-10*) codes for HF (eTable 4 in Supplement 1) and allowed the diagnosis to be at any position in inpatient, outpatient, or physician claims. We used this broad definition to increase sensitivity. Patients were only allowed to enter the study once, when they first met all inclusion and exclusion criteria.

### Follow-Up

Follow-up began the day following the first fill date, and it ended with the first of any of the following: a gap period of more than 179 days without filling a prescription for pregabalin or gabapentin (discontinuation),^2,23^ the day prior to filling a prescription for the other study drug, the day of death, the date of HF hospitalization or emergency department (ED) visit, the end of the study (December 21, 2018), the day prior to a 30-day long-term care stay or hospital stay (single stay, consecutive), the day prior to loss of full fee-for-service Medicare enrollment or enrollment in Part C, or the day prior to hospice admission (eTable 2 in Supplement 1).

### End Points

The primary study outcome was a hospitalization or ED visit with a primary diagnosis of HF. The secondary outcomes were an outpatient encounter with a primary diagnosis of HF and all-cause mortality. The outcome date was defined as the day of hospital admission, ED visit, or outpatient encounter associated with the HF diagnosis code. For the primary outcome, if prior records indicated a transfer from another hospital or ED the day of or the day before the outcome date, the outcome date was shifted to the prior admission date. HF cases were identified using *ICD-9* codes and *ICD-10* codes (eTable 4 in Supplement 1). These diagnostic algorithms have a positive predictive value (PPV) greater than 93% depending on the *ICD* version and admission type^24,25,26^ (eTable 4 in Supplement 1). For inpatient *ICD-10* codes specifically, the sensitivity is 0.80, specificity is 0.98, and PPV is 93.6%.^24^ For ED visits, the PPV for HF is 93.3%^27^; therefore, the primary end point included ED visits with a qualifying primary discharge diagnosis.

### Covariates

The study controlled for 231 covariates potentially associated with both drug exposure and outcome; this list was developed from extensive literature review and past studies.^8,10,13,14,28,29^ Covariates included demographic variables, health care utilization, various conditions abstracted from medical billing codes (Healthcare Common Procedure Coding System, *Current Procedural Terminology*, *ICD-9*, and *ICD-10*), and medications. The categories of conditions included chronic pain; trauma; cardiovascular, psychiatric, respiratory, neurological, gastrointestinal, and kidney conditions; bleeding; frailty; and others (eTables 5 and 6 in Supplement 1).

Race and ethnicity were obtained from the Medicare data and were included in the study to highlight any potential differences in use of pregabalin vs gabapentin among various racial and ethnic groups. Categories included Black or African American, non-Hispanic White, and other (American Indian or Alaska Native, Asian or Pacific Islander, Hispanic, other, or unknown).

### Statistical Analysis

Data were analyzed from March 21, 2024, to December 2, 2024. Cox proportional hazards regression models were used to estimate the hazard ratios (HRs) for pregabalin compared with gabapentin. The HRs were adjusted using inverse probability of treatment weighting. The propensity score was calculated as the probability of assignment to pregabalin given the set of 231 covariates.^30,31^ Any covariates with a frequency of less than 1% were not used in the propensity score. We trimmed patients with a propensity score less than the first percentile or greater than the 99th percentile to avoid the inclusion of patients whose treatment was predetermined by their clinical characteristics. Standardized differences were calculated and examined after applying inverse probability treatment weighting to ensure proper covariate balancing. Stratified analyses were performed by history of cardiovascular disease, race and ethnicity (Medicare Beneficiary Summary File),^32^ and sex. Cardiovascular disease history included any events 365 days prior to the outcome date (eTable 5 in Supplement 1). For a negative control, hip fracture was chosen, as it bears no causal relationship with the exposures (pregabalin or gabapentin) or the outcome (HF). We performed additional sensitivity analyses to account for unmeasured confounding by using E-values.^33,34^ All analyses were performed using Stata, version 17 (StataCorp LLC).

## Results

### Patient Characteristics

The cohort included 246 237 Medicare beneficiaries, of whom 18 622 (7.6%) were new users of pregabalin and 227 615 (92.4%) were new users of gabapentin (Figure). The cohort was predominantly female (66.8%); 33.2% were male. Median age was 73 years (IQR, 69-78 years). Of the total cohort, 7.4% were Black or African American, 79.9% were White, and 12.8% were other race or ethnicity (Table 1). The most common chronic pain diagnoses associated with prescriptions for pregabalin and gabapentin were musculoskeletal, back, and neuropathic (Table 1). Prior to inverse probability weighting, pregabalin users compared with gabapentin users overall had similar frequencies of cardiovascular diagnoses and medications as well as other types of conditions and medications. However, pregabalin users had higher use of cyclooxygenase-2 inhibitors (8.6% vs 5.0%) and duloxetine (10.1% vs 5.2%), had a higher prevalence of diabetic neuropathy (15.7% vs 11.2%) and fibromyalgia (20.5% vs 13.5%), and included a lower proportion of White patients (75.1% vs 80.3%) (eTable 5 in Supplement 1). After propensity score weighting, no covariates had a standardized difference greater than 0.10,^31^ indicating good balance (Table 1 and eTable 6 in Supplement 1).

**Figure.**
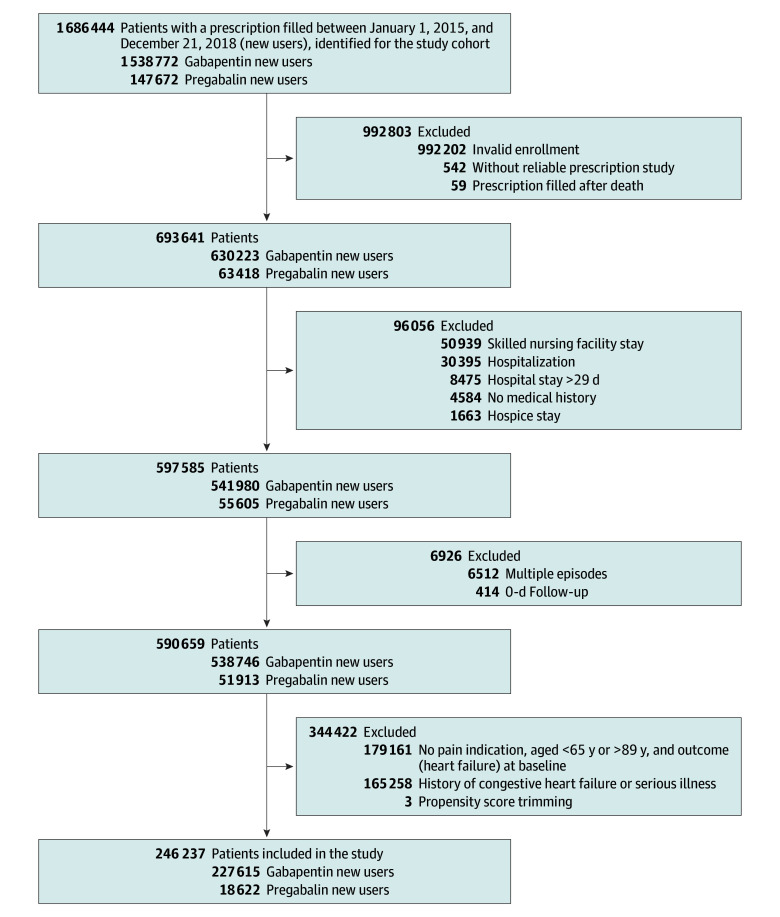
Selection and Inclusion of Participants in the Study

**Table 1.** Selected Weighted Baseline Characteristics of New Users of Pregabalin and Gabapentin

Variable	Patients, %		Standardized difference	Overall cohort, %
	Pregabalin (n = 18 622)	Gabapentin (n = 227 615)		
Age, median (IQR)	73 (69-78)	73 (69-78)	−.001	73 (69-78)
Sex				
Female	66.8	66.7	.006	66.8
Male	33.2	33.3	−.003	33.2
Race and ethnicity				
Black or African American	7.5	7.2	.013	7.4
Non-Hispanic White	79.8	79.9	−.002	79.9
Other^a^	12.7	12.9	−.008	12.8
Pain diagnoses				
Arthralgia	48.6	48.4	.500	48.5
Back pain or degenerative back disorders	67.8	68.1	−.006	68.0
Headache, including migraine	14.6	14.4	.005	14.5
Autoimmune or other rheumatic diseases	3.6	3.6	.001	3.6
Other musculoskeletal or soft tissue pain	71.9	71.6	.801	71.7
Fibromyalgia	14.7	14.1	.018	14.4
Inflammatory	22.4	22.0	.100	22.2
Neuropathic	60.7	60.8	−.002	60.8
Pain medication history				
Cyclooxygenase-2 inhibitors	5.4	5.2	.005	5.3
Cyclobenzaprine	5.9	5.8	.005	5.8
NSAIDs, nonselective	36.3	36.2	.002	36.3
Systemic oral corticosteroids	31.0	30.7	.601	30.9
Anticonvulsants, primary use pain	0.9	0.6	.042	0.7
Cardiovascular diagnoses				
Ischemic or unspecified stroke	9.9	9.8	.601	9.8
Hypertension, benign, or unspecified	79.3	79.0	.601	79.2
Myocardial infarction	3.4	3.3	.500	3.4
Other chronic ischemic heart disease	20.9	20.6	.008	20.7
Coronary artery bypass surgery	3.4	3.3	.007	3.3
Percutaneous intervention	4.5	4.4	.008	4.4
Heart failure	0	0	0.003	0
Cardiovascular medication history				
Angiotensin converting enzyme inhibitors	29.7	29.7	0	29.7
Angiotensin receptor blocker	25.9	25.8	.004	25.9
Antihypertensives, other	6.0	6.0	.002	6.0
Antiarrhythmics	6.6	6.5	.004	6.5
β-Blockers	35.0	34.8	.005	34.9
Calcium channel blockers	28.0	27.9	.002	27.9
Diuretics, potassium sparing, or hydrochlorothiazide	6.0	5.9	.002	5.9

Abbreviation: NSAIDs, nonsteroidal anti-inflammatory drugs.

^a^ Includes American Indian or Alaska Native, Asian or Pacific Islander, Hispanic, other, and unknown.

### Primary End Point and Individual Components

During 114 113 person-years of follow-up, 1470 patients (1.3%) developed new HF. The incidence of HF was 18.2 (95% CI, 15.3-21.6) per 1000 person-years for pregabalin vs 12.5 (95% CI, 11.9-13.2) per 1000 person-years for gabapentin. The adjusted HR (AHR) for pregabalin vs gabapentin was 1.48 (95% CI, 1.19-1.77) (Table 2).

**Table 2.** Hospitalization or Emergency Department Visit for Heart Failure in Pregabalin and Gabapentin New Users Unstratified and Stratified

Model	Pregabalin			Gabapentin			HR (95% CI)	
	Person-years	Events, No.	Rate, per 1000 person-years (95% CI)	Person-years	Events, No.	Rate, per 1000 person-years (95% CI)	Unadjusted	Adjusted
Primary analysis^a^	6912	126	18.2 (15.3-21.6)	107 201	1344	12.5 (11.9-13.2)	1.48 (1.23-1.77)	1.48 (1.19-1.77)
All-cause mortality	6973	67	9.6 (7.6-12.1)	107 942	844	7.8 (7.3-8.3)	1.25 (0.98-1.61)	1.26 (0.95-1.76)
History of cardiovascular disease								
No	5306	60	11.3 (8.8-14.5)	83 839	799	9.5 (8.9-10.2)	1.20 (0.93-1.56)	1.21 (0.91-1.60)
Yes	1606	66	41.1 (3.2-5.2)	23 363	545	23.3 (21.5-25.3)	1.80 (1.39-2.32)	1.85 (1.38-2.47)
Race and ethnicity								
Non-Hispanic White	5251	109	20.8 (17.2-24.9)	88 263	1075	12.2 (11.4-12.9)	1.73 (1.42-2.11)	1.65 (1.32-2.05)
Racial and ethnic minority groups	1661	17	10.2 (6.4-16.4)	18 937	269	14.2 (12.6-15.9)	0.72 (0.44-1.18)	0.69 (0.39-1.22)
Sex								
Female	4582	87	19.0 (15.4-23.3)	70 432	831	11.8 (11.0-12.6)	1.63 (1.31-2.03)	1.57 (1.23-2.00)
Male	2330	39	16.7 (12.2-22.8)	36 766	513	14.0 (12.8 15.2)	1.23 (0.88-1.70)	1.27 (0.86-1.89)

Abbreviation: HR, hazard ratio.

^a^ Hospitalization or emergency department visit.

### Stratified Analyses

We conducted stratified analyses by (1) history of cardiovascular disease, (2) race, and (3) sex. In each of these analyses, pregabalin was associated with increased risk of HF compared with gabapentin: history of cardiovascular disease (AHR, 1.85 [95% CI, 1.38-2.47]), White race (AHR, 1.65 [95% CI, 1.32-2.05]), and female (AHR, 1.57 [95% CI, 1.23-2.00]) (Table 2).

### Secondary Analyses

There was a significant increase in the risk of outpatient HF (AHR, 1.27 [95% CI, 1.02-1.58]) for patients receiving pregabalin compared with those receiving gabapentin (Table 3). However, there was no significant difference in mortality (AHR, 1.26 [95% CI, 0.95-1.76]) between the 2 groups (Table 2). We conducted a negative control outcome analysis using hip fracture as the outcome variable. We observed no difference between the 2 drugs (AHR, 1.02 [95% CI, 0.71-1.46]) (eTable 7 in Supplement 1).^35,36^ To account for unmeasured confounding, the E-values were 2.32 (95% CI, 1.19-1.77) for the adjusted and 2.32 (95% CI, 1.23-1.77) for the unadjusted point estimates for HF (eTable 8 in Supplement 1).

**Table 3.** Secondary Outcomes of Outpatient Heart Failure

Model	Pregabalin			Gabapentin			HR (95% CI)	
	Person-years	Events, No.	Rate, per 1000 person-years (95% CI)	Person-years	Events, %	Rate, per 1000 person-years (95% CI)	Unadjusted	Adjusted
Outpatient	6915	104	15.0 (12.4-18.2)	107 048	1316	12.3 (11.7-12.9)	1.27 (1.04-1.55)	1.27 (1.02-1.58)
Outpatient or inpatient	6893	152	22.0 (19.9-25.8)	106 797	1829	17.1 (16.3-17.9)	1.32 (1.12-1.56)	1.34 (1.12-1.60)

Abbreviation: HR, hazard ratio.

## Discussion

In this large retrospective cohort study of Medicare beneficiaries with chronic pain, initiation of pregabalin was associated with higher risk of incident HF compared with gabapentin initiation. We stratified by patients with a history of cardiovascular disease, the group most vulnerable to HF, and noted that risk was higher in this group. These findings further support current recommendations by the European Medicines Agency to exercise particular caution when prescribing pregabalin to older patients with cardiovascular disease.^12^

The study was designed to address limitations of prior publications that did not report an increased HF risk with pregabalin compared with gabapentin.^13,14,16^ The Medicare cohort in our study (18 622 pregabalin users) was larger than those included in the aforementioned studies (n = 1395,^13^ n = 3460,^14^ and n = 9855^15^), allowing us to detect meaningful differences between the 2 drugs. In addition, our study focused on a high-risk population—a cohort exclusively aged 65 years or older. The risk of HF essentially doubles with each decade of life and is a leading cause of mortality in older adults (age >65 years).^37^ Aging could be considered a potent risk factor for HF given that with advanced age, cardiovascular risk factors accumulate (including subclinical and silent coronary atherosclerosis), and vascular aging, characterized by arterial stiffening, occurs, with all of these factors contributing to HF incidence.^37,38,39,40^

To increase sensitivity, we used a rigorous definition of incident HF. Earlier postmarketing surveillance case reports described exacerbations of preexisting HF^7,41^ and/or reported peripheral edema.^6,41^ Edema is not a symptom exclusive to HF and could be attributed to the inhibitory effects of gabapentinoids on voltage-gated calcium channels in the peripheral arteries rather than systemic volume overload with HF. Prior studies lacked rigor in their outcome definitions. For example, one study used only 1 specific *ICD-10* code for HF (I50 HF),^8^ another study^13^ used HF diagnostic codes with lower PPV (79%), and 2 studies did not exclude those with a history of HF, examining only worsening HF.^13,14^ By restricting the cohort to patients without a history of HF, we were able to detect important differences in new acute HF between the 2 medications.

Furthermore, unlike other studies, we used the target trial design to reduce biases inherent in observational studies. We constructed and used a propensity score for adjustment of 231 relevant covariates, an approach taken by only 1 other study.^16^

Finally, we included patients with expanded indications for gabapentinoids, while previous studies have used a limited number of diagnoses and often a single indication.^8,16^ We chose to include multiple pain indications for the study drug (eTable 3 in Supplement 1), allowing for inclusion of a diverse patient population that better reflects clinical practice.

### Limitations

Several limitations should be acknowledged. The majority of the users were female and White, limiting generalizability. Because this may reflect a preferential practice among Medicare prescribers, additional studies need to be performed in male patients. The analysis was restricted to Medicare enrollees who were aged 65 years or older; therefore, findings cannot be generalized to younger patients. Additionally, we excluded Part C enrollees (private insurance companies), which could limit generalizability due to differential distribution of demographics and cardiovascular risk factors. Power was limited for racial and ethnic minority groups; more studies are needed to assess pregabalin safety in other racial and ethnic populations. Patient nonadherence could cause exposure misclassification, which if nondifferential, could bias the results in either direction.

In addition, despite controlling for 231 covariates, there was still the potential for unmeasured confounders. There are inherent limitations as to which covariates can be captured through a Medicare database. Factors such as body mass index, smoking, diet, physical activity levels, and socioeconomic status not captured by zip code could contribute to HF risk. However, the negative control and E-value analyses provided evidence that confounding was not responsible for the excess HF risk in patients treated with pregabalin.

The E-values for the adjusted and unadjusted point estimates for HF (HR) were both 2.32 (eTable 8 in Supplement 1). Given that the reported HR for the primary analysis was 1.48 (95% CI, 1.19-1.77), it seems unlikely that unmeasured confounders could pose a greater effect on HF (by an HR exceeding 2.32) compared with other risk factors, such as pregabalin or gabapentin use. We additionally attempted to check for unmeasured confounders by using a negative control outcome analysis (hip fracture) (eTable 7 in Supplement 1). We observed no difference between the 2 drugs (AHR, 1.02 [95% CI, 0.71-1.46]).

## Conclusions

In this retrospective cohort study of Medicare beneficiaries aged 65 to 89 years with chronic noncancer pain and no history of HF, new users of pregabalin had higher rates of incident HF hospitalizations or ED visits and outpatient visits compared with new users of gabapentin. Practicing clinicians should undertake a careful assessment of ongoing cardiovascular risk factors and perform adequate risk-benefit counseling for older patients before prescribing pregabalin for chronic pain.

## Notes

Supplement 1. **eTable 1.** Target Trial Summary Table **eTable 2.** End of Follow-Up Conditions **eTable 3.** Indicated *ICD-9*/*ICD-10* Diseases/Conditions for Gabapentin and Pregabalin Use **eTable 4.** Heart Failure (HF) Outcome *ICD-9*/*ICD-10* Definitions **eTable 5.** Extended Table of Variables Used to Develop Propensity Score (Unweighted) **eTable 6.** Extended Table of Variables Used to Develop Propensity Score (IPT Weighted) **eTable 7.** Negative Control for the Outcome (Hip Fracture) **eTable 8.** E-Values **eTable 9.** Proportional Assumption of Hazard Ratios **eTable 10.** Restricted Mean Survival Time **eTable 11.** Inverse Probability Weight Summary Statistics
eReferences

Supplement 2.
Data Sharing Statement
