# Supplementary material for: Initiation of Pregabalin vs Gabapentin and Development of Heart Failure
Source: JAMA Netw Open. 2025 Aug 1;8(8):e2524451. doi: 10.1001/jamanetworkopen.2025.24451 (PMC12317353; doi:10.1001/jamanetworkopen.2025.24451)
Supplement: Supplement 1. — eTable 1. Target Trial Summary Table eTable 2. End of Follow-Up Conditions eTable 3. Indicated ICD-9/ICD-10 Diseases/Conditions for Gabapentin and Pregabalin Use eTable 4. Heart Failure (HF) Outcome ICD-9/ICD-10 Definitions eTable 5. Extended Table of Variables Used to Develop Propensity Score (Unweighted) eTable 6. Extended Table of Variables Used to Develop Propensity Score (IPT Weighted) eTable 7. Negative Control for the Outcome (Hip Fracture) eTable 8. E-Values eTable 9. Proportional Assumption of Hazard Ratios eTable 10. Restricted Mean Survival Time eTable 11. Inverse Probability Weight Summary Statistics eReferences [file jamanetwopen-e2524451-s001.pdf]

Supplemental Online Content

Park EE, Daniel LL, Dickson AL, et al. Pregabalin vs gabapentin and incidence of heart failure. *JAMA Netw Open*. 2025;8(8):e2524451. doi:10.1001/jamanetworkopen.2025.24451

eTable 1. Target Trial Summary Table
eTable 2. End of Follow-Up Conditions
eTable 3. Indicated *ICD-9*/*ICD-10* Diseases/Conditions for Gabapentin and Pregabalin Use
eTable 4. Heart Failure (HF) Outcome *ICD-9*/*ICD-10* Definitions
eTable 5. Extended Table of Variables Used to Develop Propensity Score (Unweighted)
eTable 6. Extended Table of Variables Used to Develop Propensity Score (IPT Weighted)
eTable 7. Negative Control for the Outcome (Hip Fracture)
eTable 8. E-Values
eTable 9. Proportional Assumption of Hazard Ratios
eTable 10. Restricted Mean Survival Time
eTable 11. Inverse Probability Weight Summary Statistics
eReferences

This supplemental material has been provided by the authors to give readers additional information about their work.

© 2025 Park EE et al. *JAMA Network Open*.

eTable 1. Target Trial Summary Table

Component	Hypothetical trial	Cohort study
**Setting**	US Medicare beneficiaries with fee-for service enrollment.<br><br>Pragmatic large, simplified trial in context of real-world practice	Same
**Eligibility criteria**	1. Medicare Part A, B, and D<br>2. New prescription (nothing 365 days prior) of pregabalin or gabapentin during 2014-2018<br>3. > 1 outpatient visit and >1 filled prescription	Same
**Treatment Strategies**	1. Treat with pregabalin as clinically indicated or<br>2. Treat with gabapentin as clinically indicated	Same
**Treatment assignment**	Random assignment, not masked	Patient/provider driven or selected in Medicare<br><br>Observational analogue of randomization: Inverse probability of treatment weighting (IPTW) using propensity scores, given 231 covariates<br><br>Assignment occurs on date of filling of first pregabalin/gabapentin prescription<br><br>Patient/provider not blinded to treatment
**Follow-up begins**	Zero-time: day following randomization	T_0: Day following initial pregabalin/gabapentin prescription fill
**Follow-up ends**	First of:<br>1. Any study outcome<br>2. End of study (12/31/2018)<br>3. Day of death<br>4. Discontinuation of pregabalin or gabapentin prescription<br>5. Day prior to: filing another prescription for non-study drug, hospice admission; loss of Medicare enrollment; day 29 of long-term care stay or single hospital stay	Same
**Component**	**Hypothetical trial**	**Cohort study**
**Primary outcome**	Heart failure (HF) hospitalization or emergency room (ER) visits	Same
**Secondary outcomes**	Outpatient HF	Same
**Causal Contrasts**	Intention to treat effect: IPTW for baseline covariates associated with loss to follow up and/or outcome<br><br>Per protocol effect: censor individuals if deviation from assigned treatment, apply/adjust for baseline covariates associated with loss to follow up and/or outcome with IPTW	Observational analogs of intention to treat and per protocol effects: See eTables 3, 5, and 6 for details on covariates and models.
**Population summary measures**	Hazard ratios	Same
**Statistical analyses**	Cox proportional hazard regression models	Same

© 2025 Park EE et al. *JAMA Network Open*.

Target Trial Emulation Summary

This emulated target trial would study the differences in heart failure (HF) rates in patients prescribed pregabalin vs gabapentin. The design would be a large, simplified pragmatic trial in the context of real-world practice. The trial participants are U.S. Medicare beneficiaries with fee-for-service enrollment, with Medicare data facilitating identification and follow-up of trial participants. As a real-world practice study, investigators may influence assignment to pregabalin or gabapentin, but decisions regarding continuation or switching would be made by patients and their providers. eTable 1 summarizes the hypothetical trial and corresponding cohort study.

eTable 2. End of Follow-Up Conditions

Criterion	Rationale	Definition
**Last day of study**	Data availability	12/31/2018
**Study outcome (including death)**	Objective to study first occurrence any study outcome	Day of occurrence of:<br><br>Heart failure (HF) hospitalization or emergency room (ER) visits<br><br>Death
**Loss of Medicare Enrollment (or enrollment in part C)**	Data availability	First day of month in which person summary file shows absence of full fee-for-service enrollment
**Filling another prescription for non-study drug**	Contamination of control group	Day prior to other prescription
**Not filling study drug for 180 days**	Medication use required as exposure	Gap period of >179 days without filling pregabalin or gabapentin
**Long term care**<br><br>**Hospice admission**<br><br>**Single hospital stay**	Outcome could be related to a confounder	Direct admission to long term care or skilled nursing facility>29 days<br><br>Day prior to hospice admission<br><br>Day 29 of single hospital stay
**Discontinuation of pregabalin or gabapentin prescription**	Medication use required as exposure	End of user period

© 2025 Park EE et al. *JAMA Network Open*.

eTable 3. Indicated *ICD-9*/*ICD-10* Diseases/Conditions for Gabapentin and Pregabalin Use

Chronic Pain Category	*ICD-9*	*ICD-10*
**Fibromyalgia**	729.0, 729.1	M60.80, M60.811, M60.812, M60.819, M60.821, M60.822, M60.829, M60.831, M60.832, M60.839, M60.841, M60.842, M60.849, M60.851, M60.852, M60.859, M60.861, M60.862, M60.869, M60.871, M60.872, M60.879, M60.88, M60.89, M60.9, M79.0, M79.1, M79.7
**Neuropathic Pain**	053.12, 350.1, 350.2, 350.8, 350.9, 351.1, 352.1, 353.0, 353.1, 353.2, 353.3, 353.4, 353.5, 353.6, 353.8, 353.9, 354.0, 354.1, 354.2, 354.4, 354.5, 354.8, 354.9, 355.0, 355.1, 355.2, 355.3, 355.4, 355.5, 355.6, 355.71, 355.79, 355.8, 355.9, 356.1, 356.4, 356.8, 356.9, 357.1, 357.2, 357.3, 357.4, 357.5, 357.6, 357.7, 357.81, 357.82, 357.89, 357.9, 358.2, 358.8, 358.9, 721.41, 721.42, 721.91, 722.70, 722.71, 722.72, 722.73, 724.3, 724.4, 729.2, 907.1, 907.2, 907.3, 907.4, 907.5, 907.9	A52.15, B02.22, E08.40, E08.41, E08.42, E08.44, E09.40, E09.41, E09.42, E09.44, E10.40, E10.41, E10.42, E10.44, E11.40, E11.41, E11.42, E11.44, E13.40, E13.41, E13.42, E13.44, G13.0, G13.1, G50.0, G50.1, G50.8, G50.9, G51.1, G52.1, G54.0, G54.1, G54.2, G54.3, G54.4, G54.5, G54.6, G54.7, G54.8, G54.9, G55, G56.00, G56.01, G56.02, G56.03, G56.10, G56.11, G56.12, G56.13, G56.20, G56.21, G56.22, G56.23, G56.40, G56.41, G56.42, G56.43, G56.80, G56.81, G56.82, G56.83, G56.90, G56.91, G56.92, G56.93, G57.00, G57.01, G57.02, G57.03, G57.10, G57.11, G57.12, G57.13, G57.20, G57.21, G57.22, G57.23, G57.30, G57.31, G57.32, G57.33, G57.40, G57.41, G57.42, G57.43, G57.50, G57.51, G57.52, G57.53, G57.60, G57.61, G57.62, G57.63, G57.70, G57.71, G57.72, G57.73, G57.80, G57.81, G57.82, G57.83, G57.90, G57.91, G57.92, G57.93, G58.0, G58.7, G58.8, G58.9, G59, G60.0, G60.3, G60.8, G60.9, G61.1, G61.81, G61.82, G61.89, G61.9, G62.0, G62.1, G62.2, G62.81, G62.82, G62.89, G62.9, G63, G64, G65.0, G65.1, G65.2, G70.1, G70.2, G70.89, G70.9, M05.50, M05.511, M05.512, M05.519, M05.521, M05.522, M05.529, M05.531, M05.532, M05.539, M05.541, M05.542, M05.549, M05.551, M05.552, M05.559, M05.561, M05.562, M05.569, M05.571, M05.572, M05.579, M05.59, M34.83, M47.10, M47.14, M47.15, M47.16, M50.00, M50.01, M50.020, M50.021, M50.022, M50.023, M50.03, M51.04, M51.05, M51.06, M51.9, M54.10, M54.14, M54.15, M54.16, M54.17, M54.18, M54.30, M54.31, M54.32, M54.40, M54.41, M54.42, M79.2, S04.011S, S04.012S, S04.019S, S04.02XS, S04.031S, S04.032S, S04.039S, S04.041S, S04.042S, S04.049S, S04.10XS, S04.11XS, S04.12XS, S04.20XS, S04.21XS, S04.22XS, S04.30XS, S04.31XS, S04.32XS, S04.40XS, S04.41XS, S04.42XS, S04.50XS, S04.51XS, S04.52XS, S04.60XS, S04.61XS, S04.62XS, S04.70XS, S04.71XS, S04.72XS, S04.811S, S04.812S, S04.819S, S04.891S, S04.892S, S04.899S, S04.9XXS, S14.0XXS, S14.101S, S14.102S, S14.103S, S14.104S, S14.105S, S14.106S, S14.107S, S14.108S, S14.109S, S14.111S, S14.112S, S14.113S, S14.114S, S14.115S, S14.116S, S14.117S, S14.118S, S14.119S, S14.121S, S14.122S, S14.123S, S14.124S, S14.125S, S14.126S, S14.127S, S14.128S, S14.129S, S14.131S, S14.132S, S14.133S, S14.134S, S14.135S, S14.136S, S14.137S, S14.138S, S14.139S, S14.141S, S14.142S, S14.143S, S14.144S, S14.145S, S14.146S, S14.147S, S14.148S, S14.149S, S14.151S, S14.152S, S14.153S, S14.154S, S14.155S, S14.156S, S14.157S, S14.158S, S14.159S, S14.2XXS, S14.3XXS, S14.4XXS, S14.5XXS, S14.8XXS, S14.9XXS, S24.0XXS, S24.101S, S24.102S, S24.103S, S24.104S, S24.109S, S24.111S, S24.112S, S24.113S, S24.114S, S24.119S, S24.131S, S24.132S, S24.133S, S24.134S, S24.139S, S24.141S, S24.142S, S24.143S, S24.144S, S24.149S, S24.151S, S24.152S, S24.153S, S24.154S, S24.159S, S24.2XXS, S24.3XXS, S24.4XXS, S24.8XXS, S24.9XXS, S34.01XS, S34.02XS, S34.101S, S34.102S, S34.103S, S34.104S, S34.105S, S34.109S, S34.111S, S34.112S, S34.113S, S34.114S, S34.115S, S34.119S, S34.121S, S34.122S, S34.123S, S34.124S, S34.125S, S34.129S, S34.131S, S34.132S, S34.139S, S34.21XS, S34.22XS, S34.3XXS, S34.4XXS, S34.5XXS, S34.6XXS, S34.8XXS, S34.9XXS, S44.00XS, S44.01XS, S44.02XS, S44.10XS, S44.11XS, S44.12XS, S44.20XS, S44.21XS, S44.22XS, S44.30XS, S44.31XS, S44.32XS, S44.40XS, S44.41XS, S44.42XS, S44.50XS, S44.51XS, S44.52XS, S44.8X1S, S44.8X2S, S44.8X9S, S44.90XS, S44.91XS, S44.92XS, S54.00XS, S54.01XS, S54.02XS, S54.10XS, S54.11XS, S54.12XS, S54.20XS, S54.21XS, S54.22XS, S54.30XS, S54.31XS, S54.32XS, S54.8X1S, S54.8X2S, S54.8X9S, S54.90XS, S54.91XS, S54.92XS, S64.00XS, S64.01XS, S64.02XS, S64.10XS, S64.11XS, S64.12XS, S64.20XS, S64.21XS, S64.22XS, S64.30XS, S64.31XS, S64.32XS,

© 2025 Park EE et al. *JAMA Network Open*.

		S64.40XS, S64.490S, S64.491S, S64.492S, S64.493S, S64.494S, S64.495S, S64.496S, S64.497S, S64.498S, S64.8X1S, S64.8X2S, S64.8X9S, S64.90XS, S64.91XS, S64.92XS, S74.00XS, S74.01XS, S74.02XS, S74.10XS, S74.11XS, S74.12XS, S74.20XS, S74.21XS, S74.22XS, S74.8X1S, S74.8X2S, S74.8X9S, S74.90XS, S74.91XS, S74.92XS, S84.00XS, S84.01XS, S84.02XS, S84.10XS, S84.11XS, S84.12XS, S84.20XS, S84.21XS, S84.22XS, S84.801S, S84.802S, S84.809S, S84.90XS, S84.91XS, S84.92XS, S94.00XS, S94.01XS, S94.02XS, S94.10XS, S94.11XS, S94.12XS, S94.20XS, S94.21XS, S94.22XS, S94.30XS, S94.31XS, S94.32XS, S94.8X1S, S94.8X2S, S94.8X9S, S94.90XS, S94.91XS, S94.92XS
**Back pain / degenerative back disorders**	720.1, 721.0, 721.2, 721.3, 721.90, 722.0, 722.10, 722.11, 722.2, 722.30, 722.31, 722.32, 722.39, 722.4, 722.51, 722.52, 722.6, 722.70, 722.71, 722.72, 722.73, 722.80, 722.81, 722.82, 722.83, 722.90, 722.91, 722.92, 722.93, 723.0, 723.1, 723.2, 723.3, 723.4, 723.5, 723.6, 723.8, 723.9, 724.00, 724.01, 724.02, 724.03, 724.09, 724.1, 724.2, 724.5, 724.6, 724.70, 724.71, 724.79, 724.8, 724.9, 737.0, 737.10, 737.11, 737.12, 737.19, 737.20, 737.21, 737.22, 737.29, 737.30, 737.31, 737.32, 737.33, 737.34, 737.39, 737.40, 737.41, 737.42, 737.43, 737.8, 737.9, 738.4, 738.5, 738.6, 739.1, 739.2, 739.3, 739.4, 739.5, 756.10, 756.11, 756.12, 756.13, 756.14, 756.15, 756.16, 756.17, 756.19	A18.01, M40.00, M40.03, M40.04, M40.05, M40.10, M40.12, M40.13, M40.14, M40.15, M40.202, M40.203, M40.204, M40.205, M40.209, M40.292, M40.293, M40.294, M40.295, M40.299, M40.30, M40.35, M40.36, M40.37, M40.40, M40.45, M40.46, M40.47, M40.50, M40.55, M40.56, M40.57, M41.00, M41.02, M41.03, M41.04, M41.05, M41.06, M41.07, M41.08, M41.112, M41.113, M41.114, M41.115, M41.116, M41.117, M41.119, M41.122, M41.123, M41.124, M41.125, M41.126, M41.127, M41.129, M41.20, M41.22, M41.23, M41.24, M41.25, M41.26, M41.27, M41.30, M41.34, M41.35, M41.40, M41.41, M41.42, M41.43, M41.44, M41.45, M41.46, M41.47, M41.50, M41.52, M41.53, M41.54, M41.55, M41.56, M41.57, M41.80, M41.82, M41.83, M41.84, M41.85, M41.86, M41.87, M41.9, M43.00, M43.01, M43.02, M43.03, M43.04, M43.05, M43.06, M43.07, M43.08, M43.09, M43.10, M43.11, M43.12, M43.13, M43.14, M43.15, M43.16, M43.17, M43.18, M43.19, M43.20, M43.21, M43.22, M43.23, M43.24, M43.25, M43.26, M43.27, M43.28, M43.6, M43.8X1, M43.8X2, M43.8X3, M43.8X4, M43.8X5, M43.8X6, M43.8X7, M43.8X8, M43.8X9, M43.9, M46.00, M46.01, M46.02, M46.03, M46.04, M46.05, M46.06, M46.07, M46.08, M46.09, M46.40, M46.41, M46.42, M46.43, M46.44, M46.45, M46.46, M46.47, M46.48, M46.49, M47.20, M47.21, M47.22, M47.23, M47.24, M47.25, M47.26, M47.27, M47.28, M47.811, M47.812, M47.813, M47.814, M47.815, M47.816, M47.817, M47.818, M47.819, M47.891, M47.892, M47.893, M47.894, M47.895, M47.896, M47.897, M47.898, M47.899, M47.9, M48.00, M48.01, M48.02, M48.03, M48.04, M48.05, M48.061, M48.062, M48.07, M48.08, M50.00, M50.01, M50.020, M50.021, M50.022, M50.023, M50.03, M50.10, M50.11, M50.120, M50.121, M50.122, M50.123, M50.13, M50.20, M50.21, M50.220, M50.221, M50.222, M50.223, M50.23, M50.30, M50.31, M50.320, M50.321, M50.322, M50.323, M50.33, M50.80, M50.81, M50.820, M50.821, M50.822, M50.823, M50.83, M50.90, M50.91, M50.920, M50.921, M50.922, M50.923, M50.93, M51.04, M51.05, M51.06, M51.14, M51.15, M51.16, M51.17, M51.24, M51.25, M51.26, M51.27, M51.34, M51.35, M51.36, M51.37, M51.44, M51.45, M51.46, M51.47, M51.84, M51.85, M51.86, M51.87, M51.9, M53.0, M53.1, M53.2X7, M53.2X8, M53.3, M53.80, M53.81, M53.82, M53.83, M53.84, M53.85, M53.86, M53.87, M53.88, M53.9, M54.00, M54.01, M54.02, M54.03, M54.04, M54.05, M54.06, M54.07, M54.08, M54.09, M54.11, M54.12, M54.13, M54.2, M54.5, M54.6, M54.81, M54.89, M54.9, M62.830, M95.5, M96.1, M96.2, M96.3, M96.4, M96.5, M99.01, M99.02, M99.03, M99.04, M99.05, M99.20, M99.21, M99.22, M99.23, M99.24, M99.25, M99.26, M99.27, M99.28, M99.29, M99.30, M99.31, M99.32, M99.33, M99.34, M99.35, M99.36, M99.37, M99.38, M99.39, M99.40, M99.41, M99.42, M99.43, M99.44, M99.45, M99.46, M99.47, M99.48, M99.49, M99.50, M99.51, M99.52, M99.53, M99.54, M99.55, M99.56, M99.57, M99.58, M99.59, M99.60, M99.61, M99.62, M99.63, M99.64, M99.65, M99.66, M99.67, M99.68, M99.69, M99.70, M99.71, M99.72, M99.73, M99.74, M99.75, M99.76, M99.77, M99.78, M99.79, M99.83, M99.84, M99.85, M99.89, Q76.0, Q76.1, Q76.2, Q76.411, Q76.412, Q76.413, Q76.414, Q76.415, Q76.419, Q76.49
**Inflammatory arthritis / arthropathy**	274.00, 274.01, 274.02, 274.03, 274.10, 274.19, 274.81, 274.82, 274.89, 274.9,	A18.01, A52.16, E08.610, E09.610, E10.610, E11.610, E13.610, M02.00, M02.011, M02.012, M02.019, M02.021, M02.022, M02.029, M02.031, M02.032, M02.039, M02.041, M02.042, M02.049, M02.051, M02.052, M02.059, M02.061, M02.062, M02.069, M02.071, M02.072, M02.079, M02.08, M02.09, M02.10, M02.111, M02.112, M02.119, M02.121, M02.122, M02.129, M02.131, M02.132,

© 2025 Park EE et al. *JAMA Network Open*.

	711.10, 711.11,	M02.139, M02.141, M02.142, M02.149, M02.151, M02.152, M02.159, M02.161,
	711.12, 711.13,	M02.162, M02.169, M02.171, M02.172, M02.179, M02.18, M02.19, M02.20,
	711.14, 711.15,	M02.211, M02.212, M02.219, M02.221, M02.222, M02.229, M02.231, M02.232,
	711.16, 711.17,	M02.239, M02.241, M02.242, M02.249, M02.251, M02.252, M02.259, M02.261,
	711.18, 711.19,	M02.262, M02.269, M02.271, M02.272, M02.279, M02.28, M02.29, M02.30,
	711.20, 711.21,	M02.311, M02.312, M02.319, M02.321, M02.322, M02.329, M02.331, M02.332,
	711.22, 711.23,	M02.339, M02.341, M02.342, M02.349, M02.351, M02.352, M02.359, M02.361,
	711.24, 711.25,	M02.362, M02.369, M02.371, M02.372, M02.379, M02.38, M02.39, M02.9,
	711.26, 711.27,	M05.00, M05.011, M05.012, M05.019, M05.021, M05.022, M05.029, M05.031,
	711.28, 711.29,	M05.032, M05.039, M05.041, M05.042, M05.049, M05.051, M05.052, M05.059,
	711.30, 711.31,	M05.061, M05.062, M05.069, M05.071, M05.072, M05.079, M05.09, M05.10,
	711.32, 711.33,	M05.111, M05.112, M05.119, M05.121, M05.122, M05.129, M05.131, M05.132,
	711.34, 711.35,	M05.139, M05.141, M05.142, M05.149, M05.151, M05.152, M05.159, M05.161,
	711.36, 711.37,	M05.162, M05.169, M05.171, M05.172, M05.179, M05.19, M05.20, M05.211,
	711.38, 711.39,	M05.212, M05.219, M05.221, M05.222, M05.229, M05.231, M05.232, M05.239,
	712.10, 712.11,	M05.241, M05.242, M05.249, M05.251, M05.252, M05.259, M05.261, M05.262,
	712.12, 712.13,	M05.269, M05.271, M05.272, M05.279, M05.29, M05.30, M05.311, M05.312,
	712.14, 712.15,	M05.319, M05.321, M05.322, M05.329, M05.331, M05.332, M05.339, M05.341,
	712.16, 712.17,	M05.342, M05.349, M05.351, M05.352, M05.359, M05.361, M05.362, M05.369,
	712.18, 712.19,	M05.371, M05.372, M05.379, M05.39, M05.40, M05.411, M05.412, M05.419,
	712.20, 712.21,	M05.421, M05.422, M05.429, M05.431, M05.432, M05.439, M05.441, M05.442,
	712.22, 712.23,	M05.449, M05.451, M05.452, M05.459, M05.461, M05.462, M05.469, M05.471,
	712.24, 712.25,	M05.472, M05.479, M05.49, M05.50, M05.511, M05.512, M05.519, M05.521,
	712.26, 712.27,	M05.522, M05.529, M05.531, M05.532, M05.539, M05.541, M05.542, M05.549,
	712.28, 712.29,	M05.551, M05.552, M05.559, M05.561, M05.562, M05.569, M05.571, M05.572,
	712.30, 712.31,	M05.579, M05.59, M05.60, M05.611, M05.612, M05.619, M05.621, M05.622,
	712.32, 712.33,	M05.629, M05.631, M05.632, M05.639, M05.641, M05.642, M05.649, M05.651,
	712.34, 712.35,	M05.652, M05.659, M05.661, M05.662, M05.669, M05.671, M05.672, M05.679,
	712.36, 712.37,	M05.69, M05.70, M05.711, M05.712, M05.719, M05.721, M05.722, M05.729,
	712.38, 712.39,	M05.731, M05.732, M05.739, M05.741, M05.742, M05.749, M05.751, M05.752,
	712.80, 712.81,	M05.759, M05.761, M05.762, M05.769, M05.771, M05.772, M05.779, M05.79,
	712.82, 712.83,	M05.80, M05.811, M05.812, M05.819, M05.821, M05.822, M05.829, M05.831,
	712.84, 712.85,	M05.832, M05.839, M05.841, M05.842, M05.849, M05.851, M05.852, M05.859,
	712.86, 712.87,	M05.861, M05.862, M05.869, M05.871, M05.872, M05.879, M05.89, M05.9,
	712.88, 712.89,	M06.00, M06.011, M06.012, M06.019, M06.021, M06.022, M06.029, M06.031,
	712.90, 712.91,	M06.032, M06.039, M06.041, M06.042, M06.049, M06.051, M06.052, M06.059,
	712.92, 712.93,	M06.061, M06.062, M06.069, M06.071, M06.072, M06.079, M06.08, M06.09,
	712.94, 712.95,	M06.1, M06.20, M06.211, M06.212, M06.219, M06.221, M06.222, M06.229,
	712.96, 712.97,	M06.231, M06.232, M06.239, M06.241, M06.242, M06.249, M06.251, M06.252,
	712.98, 712.99,	M06.259, M06.261, M06.262, M06.269, M06.271, M06.272, M06.279, M06.28,
	713.0, 713.1,	M06.29, M06.30, M06.311, M06.312, M06.319, M06.321, M06.322, M06.329,
	713.2, 713.3,	M06.331, M06.332, M06.339, M06.341, M06.342, M06.349, M06.351, M06.352,
	713.4, 713.5,	M06.359, M06.361, M06.362, M06.369, M06.371, M06.372, M06.379, M06.38,
	713.6, 713.7,	M06.39, M06.4, M06.80, M06.811, M06.812, M06.819, M06.821, M06.822,
	713.8, 714.0,	M06.829, M06.831, M06.832, M06.839, M06.841, M06.842, M06.849, M06.851,
	714.1, 714.2,	M06.852, M06.859, M06.861, M06.862, M06.869, M06.871, M06.872, M06.879,
	714.30, 714.31,	M06.88, M06.89, M06.9, M07.60, M07.611, M07.612, M07.619, M07.621,
	714.32, 714.33,	M07.622, M07.629, M07.631, M07.632, M07.639, M07.641, M07.642, M07.649,
	714.4, 714.81,	M07.651, M07.652, M07.659, M07.661, M07.662, M07.669, M07.671, M07.672,
	714.89, 714.9,	M07.679, M07.68, M07.69, M08.00, M08.011, M08.012, M08.019, M08.021,
	716.40, 716.41,	M08.022, M08.029, M08.031, M08.032, M08.039, M08.041, M08.042, M08.049,
	716.42, 716.43,	M08.051, M08.052, M08.059, M08.061, M08.062, M08.069, M08.071, M08.072,
	716.44, 716.45,	M08.079, M08.08, M08.09, M08.1, M08.20, M08.211, M08.212, M08.219,
	716.46, 716.47,	M08.221, M08.222, M08.229, M08.231, M08.232, M08.239, M08.241, M08.242,
	716.48, 716.49,	M08.249, M08.251, M08.252, M08.259, M08.261, M08.262, M08.269, M08.271,
	716.50, 716.51,	M08.272, M08.279, M08.28, M08.29, M08.3, M08.40, M08.411, M08.412,
	716.52, 716.53,	M08.419, M08.421, M08.422, M08.429, M08.431, M08.432, M08.439, M08.441,
	716.54, 716.55,	M08.442, M08.449, M08.451, M08.452, M08.459, M08.461, M08.462, M08.469,
	716.56, 716.57,	M08.471, M08.472, M08.479, M08.48, M08.80, M08.811, M08.812, M08.819,
	716.58, 716.59,	M08.821, M08.822, M08.829, M08.831, M08.832, M08.839, M08.841, M08.842,

	716.60, 716.61, 716.62, 716.63, 716.64, 716.65, 716.66, 716.67, 716.68, 720.0, 720.2, 720.81, 720.89, 720.9, 727.00, 727.01, 727.03, 727.04, 727.05, 727.06, 727.09	M08.849, M08.851, M08.852, M08.859, M08.861, M08.862, M08.869, M08.871, M08.872, M08.879, M08.88, M08.89, M08.90, M08.911, M08.912, M08.919, M08.921, M08.922, M08.929, M08.931, M08.932, M08.939, M08.941, M08.942, M08.949, M08.951, M08.952, M08.959, M08.961, M08.962, M08.969, M08.971, M08.972, M08.979, M08.98, M08.99, M10.00, M10.011, M10.012, M10.019, M10.021, M10.022, M10.029, M10.031, M10.032, M10.039, M10.041, M10.042, M10.049, M10.051, M10.052, M10.059, M10.061, M10.062, M10.069, M10.071, M10.072, M10.079, M10.08, M10.09, M10.10, M10.111, M10.112, M10.119, M10.121, M10.122, M10.129, M10.131, M10.132, M10.139, M10.141, M10.142, M10.149, M10.151, M10.152, M10.159, M10.161, M10.162, M10.169, M10.171, M10.172, M10.179, M10.18, M10.19, M10.20, M10.211, M10.212, M10.219, M10.221, M10.222, M10.229, M10.231, M10.232, M10.239, M10.241, M10.242, M10.249, M10.251, M10.252, M10.259, M10.261, M10.262, M10.269, M10.271, M10.272, M10.279, M10.28, M10.29, M10.30, M10.311, M10.312, M10.319, M10.321, M10.322, M10.329, M10.331, M10.332, M10.339, M10.341, M10.342, M10.349, M10.351, M10.352, M10.359, M10.361, M10.362, M10.369, M10.371, M10.372, M10.379, M10.38, M10.39, M10.40, M10.411, M10.412, M10.419, M10.421, M10.422, M10.429, M10.431, M10.432, M10.439, M10.441, M10.442, M10.449, M10.451, M10.452, M10.459, M10.461, M10.462, M10.469, M10.471, M10.472, M10.479, M10.48, M10.49, M10.9, M11.00, M11.011, M11.012, M11.019, M11.021, M11.022, M11.029, M11.031, M11.032, M11.039, M11.041, M11.042, M11.049, M11.051, M11.052, M11.059, M11.061, M11.062, M11.069, M11.071, M11.072, M11.079, M11.08, M11.09, M11.10, M11.111, M11.112, M11.119, M11.121, M11.122, M11.129, M11.131, M11.132, M11.139, M11.141, M11.142, M11.149, M11.151, M11.152, M11.159, M11.161, M11.162, M11.169, M11.171, M11.172, M11.179, M11.18, M11.19, M11.20, M11.211, M11.212, M11.219, M11.221, M11.222, M11.229, M11.231, M11.232, M11.239, M11.241, M11.242, M11.249, M11.251, M11.252, M11.259, M11.261, M11.262, M11.269, M11.271, M11.272, M11.279, M11.28, M11.29, M11.80, M11.811, M11.812, M11.819, M11.821, M11.822, M11.829, M11.831, M11.832, M11.839, M11.841, M11.842, M11.849, M11.851, M11.852, M11.859, M11.861, M11.862, M11.869, M11.871, M11.872, M11.879, M11.88, M11.89, M11.9, M12.00, M12.011, M12.012, M12.019, M12.021, M12.022, M12.029, M12.031, M12.032, M12.039, M12.041, M12.042, M12.049, M12.051, M12.052, M12.059, M12.061, M12.062, M12.069, M12.071, M12.072, M12.079, M12.08, M12.09, M12.80, M12.811, M12.812, M12.819, M12.821, M12.822, M12.829, M12.831, M12.832, M12.839, M12.841, M12.842, M12.849, M12.851, M12.852, M12.859, M12.861, M12.862, M12.869, M12.871, M12.872, M12.879, M12.88, M12.89, M13.0, M13.10, M13.111, M13.112, M13.119, M13.121, M13.122, M13.129, M13.131, M13.132, M13.139, M13.141, M13.142, M13.149, M13.151, M13.152, M13.159, M13.161, M13.162, M13.169, M13.171, M13.172, M13.179, M14.60, M14.611, M14.612, M14.619, M14.621, M14.622, M14.629, M14.631, M14.632, M14.639, M14.641, M14.642, M14.649, M14.651, M14.652, M14.659, M14.661, M14.662, M14.669, M14.671, M14.672, M14.679, M14.68, M14.69, M14.80, M14.811, M14.812, M14.819, M14.821, M14.822, M14.829, M14.831, M14.832, M14.839, M14.841, M14.842, M14.849, M14.851, M14.852, M14.859, M14.861, M14.862, M14.869, M14.871, M14.872, M14.879, M14.88, M14.89, M1A.00X0, M1A.00X1, M1A.0110, M1A.0111, M1A.0120, M1A.0121, M1A.0190, M1A.0191, M1A.0210, M1A.0211, M1A.0220, M1A.0221, M1A.0290, M1A.0291, M1A.0310, M1A.0311, M1A.0320, M1A.0321, M1A.0390, M1A.0391, M1A.0410, M1A.0411, M1A.0420, M1A.0421, M1A.0490, M1A.0491, M1A.0510, M1A.0511, M1A.0520, M1A.0521, M1A.0590, M1A.0591, M1A.0610, M1A.0611, M1A.0620, M1A.0621, M1A.0690, M1A.0691, M1A.0710, M1A.0711, M1A.0720, M1A.0721, M1A.0790, M1A.0791, M1A.08X0, M1A.08X1, M1A.09X0, M1A.09X1, M1A.20X0, M1A.20X1, M1A.2110, M1A.2111, M1A.2120, M1A.2121, M1A.2190, M1A.2191, M1A.2210, M1A.2211, M1A.2220, M1A.2221, M1A.2290, M1A.2291, M1A.2310, M1A.2311, M1A.2320, M1A.2321, M1A.2390, M1A.2391, M1A.2410, M1A.2411, M1A.2420, M1A.2421, M1A.2490, M1A.2491, M1A.2510, M1A.2511, M1A.2520, M1A.2521, M1A.2590, M1A.2591, M1A.2610, M1A.2611, M1A.2620, M1A.2621, M1A.2690, M1A.2691, M1A.2710, M1A.2711, M1A.2720, M1A.2721, M1A.2790, M1A.2791, M1A.28X0,

© 2025 Park EE et al. *JAMA Network Open*.

		M1A.28X1, M1A.29X0, M1A.29X1, M1A.30X0, M1A.30X1, M1A.3110, M1A.3111, M1A.3120, M1A.3121, M1A.3190, M1A.3191, M1A.3210, M1A.3211, M1A.3220, M1A.3221, M1A.3290, M1A.3291, M1A.3310, M1A.3311, M1A.3320, M1A.3321, M1A.3390, M1A.3391, M1A.3410, M1A.3411, M1A.3420, M1A.3421, M1A.3490, M1A.3491, M1A.3510, M1A.3511, M1A.3520, M1A.3521, M1A.3590, M1A.3591, M1A.3610, M1A.3611, M1A.3620, M1A.3621, M1A.3690, M1A.3691, M1A.3710, M1A.3711, M1A.3720, M1A.3721, M1A.3790, M1A.3791, M1A.38X0, M1A.38X1, M1A.39X0, M1A.39X1, M1A.40X0, M1A.40X1, M1A.4110, M1A.4111, M1A.4120, M1A.4121, M1A.4190, M1A.4191, M1A.4210, M1A.4211, M1A.4220, M1A.4221, M1A.4290, M1A.4291, M1A.4310, M1A.4311, M1A.4320, M1A.4321, M1A.4390, M1A.4391, M1A.4410, M1A.4411, M1A.4420, M1A.4421, M1A.4490, M1A.4491, M1A.4510, M1A.4511, M1A.4520, M1A.4521, M1A.4590, M1A.4591, M1A.4610, M1A.4611, M1A.4620, M1A.4621, M1A.4690, M1A.4691, M1A.4710, M1A.4711, M1A.4720, M1A.4721, M1A.4790, M1A.4791, M1A.48X0, M1A.48X1, M1A.49X0, M1A.49X1, M1A.9XX0, M1A.9XX1, M35.2, M36.1, M36.2, M36.3, M36.4, M45.0, M45.1, M45.2, M45.3, M45.4, M45.5, M45.6, M45.7, M45.8, M45.9, M46.1, M46.50, M46.51, M46.52, M46.53, M46.54, M46.55, M46.56, M46.57, M46.58, M46.59, M46.80, M46.81, M46.82, M46.83, M46.84, M46.85, M46.86, M46.87, M46.88, M46.89, M46.90, M46.91, M46.92, M46.93, M46.94, M46.95, M46.96, M46.97, M46.98, M46.99, M48.8X1, M48.8X2, M48.8X3, M48.8X4, M48.8X5, M48.8X6, M48.8X7, M48.8X8, M48.8X9, M49.80, M49.81, M49.82, M49.83, M49.84, M49.85, M49.86, M49.87, M49.88, M49.89, M65.10, M65.111, M65.112, M65.119, M65.121, M65.122, M65.129, M65.131, M65.132, M65.139, M65.141, M65.142, M65.149, M65.151, M65.152, M65.159, M65.161, M65.162, M65.169, M65.171, M65.172, M65.179, M65.18, M65.19, M65.30, M65.311, M65.312, M65.319, M65.321, M65.322, M65.329, M65.331, M65.332, M65.339, M65.341, M65.342, M65.349, M65.351, M65.352, M65.359, M65.4, M65.80, M65.811, M65.812, M65.819, M65.821, M65.822, M65.829, M65.831, M65.832, M65.839, M65.841, M65.842, M65.849, M65.851, M65.852, M65.859, M65.861, M65.862, M65.869, M65.871, M65.872, M65.879, M65.88, M65.89, M65.9, M67.30, M67.311, M67.312, M67.319, M67.321, M67.322, M67.329, M67.331, M67.332, M67.339, M67.341, M67.342, M67.349, M67.351, M67.352, M67.359, M67.361, M67.362, M67.369, M67.371, M67.372, M67.379, M67.38, M67.39
**Arthralgia**	719.40, 719.41, 719.42, 719.43, 719.44, 719.45, 719.46, 719.47, 719.48, 719.49	M25.50, M25.511, M25.512, M25.519, M25.521, M25.522, M25.529, M25.531, M25.532, M25.539, M25.541, M25.542, M25.549, M25.551, M25.552, M25.559, M25.561, M25.562, M25.569, M25.571, M25.572, M25.579, M79.646
**Autoimmune / other rheumatic diseases**	446.0, 446.1, 446.20, 446.21, 446.29, 446.4, 446.5, 446.7, 710.0, 710.1, 710.2, 710.3, 710.4, 710.5, 710.8, 710.9, 725	M04.2, M04.8, M04.9, M30.0, M30.1, M30.2, M30.3, M30.8, M31.0, M31.30, M31.31, M31.4, M31.5, M31.6, M31.7, M32.0, M32.10, M32.11, M32.12, M32.13, M32.14, M32.15, M32.19, M32.8, M32.9, M33.00, M33.01, M33.02, M33.03, M33.09, M33.10, M33.11, M33.12, M33.13, M33.19, M33.20, M33.21, M33.22, M33.29, M33.90, M33.91, M33.92, M33.93, M33.99, M34.0, M34.1, M34.2, M34.81, M34.82, M34.83, M34.89, M34.9, M35.00, M35.01, M35.02, M35.03, M35.04, M35.09, M35.1, M35.3, M35.5, M35.8, M35.9, M36.0, M36.8
**Other musculoskeletal / soft tissue pain**	307.80, 307.89, 524.60, 524.61, 524.62, 524.63, 524.64, 524.69, 611.71, 715.00, 715.04, 715.09, 715.10, 715.11, 715.12, 715.13, 715.14, 715.15, 715.16, 715.17, 715.18, 715.20, 715.21, 715.22, 715.23, 715.24, 715.25, 715.26,	E08.618, E09.618, E10.618, E11.618, E13.618, F45.41, F45.42, M07.60, M07.611, M07.612, M07.619, M07.621, M07.622, M07.629, M07.631, M07.632, M07.639, M07.641, M07.642, M07.649, M07.651, M07.652, M07.659, M07.661, M07.662, M07.669, M07.671, M07.672, M07.679, M07.68, M07.69, M12.10, M12.111, M12.112, M12.119, M12.121, M12.122, M12.129, M12.131, M12.132, M12.139, M12.141, M12.142, M12.149, M12.151, M12.152, M12.159, M12.161, M12.162, M12.169, M12.171, M12.172, M12.179, M12.18, M12.19, M12.20, M12.211, M12.212, M12.219, M12.221, M12.222, M12.229, M12.231, M12.232, M12.239, M12.241, M12.242, M12.249, M12.251, M12.252, M12.259, M12.261, M12.262, M12.269, M12.271, M12.272, M12.279, M12.28, M12.29, M12.50, M12.511, M12.512, M12.519, M12.521, M12.522, M12.529, M12.531, M12.532, M12.539, M12.541, M12.542, M12.549, M12.551, M12.552, M12.559, M12.561, M12.562, M12.569, M12.571, M12.572, M12.579, M12.58, M12.59, M12.80, M12.811, M12.812, M12.819, M12.821, M12.822, M12.829, M12.831, M12.832,

© 2025 Park EE et al. *JAMA Network Open*.

	715.27, 715.28, 715.30, 715.31, 715.32, 715.33, 715.34, 715.35, 715.36, 715.37, 715.38, 715.80, 715.89, 715.90, 715.91, 715.92, 715.93, 715.94, 715.95, 715.96, 715.97, 715.98, 716.00, 716.01, 716.02, 716.03, 716.04, 716.05, 716.06, 716.07, 716.08, 716.09, 716.10, 716.11, 716.12, 716.13, 716.14, 716.15, 716.16, 716.17, 716.18, 716.19, 716.20, 716.21, 716.22, 716.23, 716.24, 716.25, 716.26, 716.27, 716.28, 716.29, 716.30, 716.31, 716.32, 716.33, 716.34, 716.35, 716.36, 716.37, 716.38, 716.39, 716.80, 716.81, 716.82, 716.83, 716.84, 716.85, 716.86, 716.87, 716.88, 716.89, 716.90, 716.91, 716.92, 716.93, 716.94, 716.95, 716.96, 716.97, 716.98, 716.99, 717.0, 717.1, 717.2, 717.3, 717.40, 717.41, 717.42, 717.43, 717.49, 717.5, 717.6, 717.7, 717.81, 717.82, 717.83, 717.84, 717.85, 717.89, 717.9, 718.00, 718.01, 718.02, 718.03, 718.04, 718.05, 718.07, 718.08, 718.09, 718.10, 718.11, 718.12, 718.13, 718.14, 718.15, 718.17, 718.18, 718.19, 718.20,	M12.839, M12.841, M12.842, M12.849, M12.851, M12.852, M12.859, M12.861, M12.862, M12.869, M12.871, M12.872, M12.879, M12.88, M12.89, M12.9, M13.80, M13.811, M13.812, M13.819, M13.821, M13.822, M13.829, M13.831, M13.832, M13.839, M13.841, M13.842, M13.849, M13.851, M13.852, M13.859, M13.861, M13.862, M13.869, M13.871, M13.872, M13.879, M13.88, M13.89, M15.0, M15.1, M15.2, M15.3, M15.4, M15.8, M15.9, M16.0, M16.10, M16.11, M16.12, M16.2, M16.30, M16.31, M16.32, M16.4, M16.50, M16.51, M16.52, M16.6, M16.7, M16.9, M17.0, M17.10, M17.11, M17.12, M17.2, M17.30, M17.31, M17.32, M17.4, M17.5, M17.9, M18.0, M18.10, M18.11, M18.12, M18.2, M18.30, M18.31, M18.32, M18.4, M18.50, M18.51, M18.52, M18.9, M19.011, M19.012, M19.019, M19.021, M19.022, M19.029, M19.031, M19.032, M19.039, M19.041, M19.042, M19.049, M19.071, M19.072, M19.079, M19.111, M19.112, M19.119, M19.121, M19.122, M19.129, M19.131, M19.132, M19.139, M19.141, M19.142, M19.149, M19.171, M19.172, M19.179, M19.211, M19.212, M19.219, M19.221, M19.222, M19.229, M19.231, M19.232, M19.239, M19.241, M19.242, M19.249, M19.271, M19.272, M19.279, M19.90, M19.91, M19.92, M19.93, M20.001, M20.002, M20.009, M20.011, M20.012, M20.019, M20.021, M20.022, M20.029, M20.031, M20.032, M20.039, M20.091, M20.092, M20.099, M20.10, M20.11, M20.12, M20.20, M20.21, M20.22, M20.30, M20.31, M20.32, M20.40, M20.41, M20.42, M20.5X1, M20.5X2, M20.5X9, M20.60, M20.61, M20.62, M21.00, M21.021, M21.022, M21.029, M21.051, M21.052, M21.059, M21.061, M21.062, M21.069, M21.071, M21.072, M21.079, M21.10, M21.121, M21.122, M21.129, M21.151, M21.152, M21.159, M21.161, M21.162, M21.169, M21.171, M21.172, M21.179, M21.20, M21.211, M21.212, M21.219, M21.221, M21.222, M21.229, M21.231, M21.232, M21.239, M21.241, M21.242, M21.249, M21.251, M21.252, M21.259, M21.261, M21.262, M21.269, M21.271, M21.272, M21.279, M21.331, M21.332, M21.339, M21.371, M21.372, M21.379, M21.511, M21.512, M21.519, M21.521, M21.522, M21.529, M21.531, M21.532, M21.539, M21.541, M21.542, M21.549, M21.611, M21.612, M21.619, M21.621, M21.622, M21.629, M21.6X1, M21.6X2, M21.6X9, M21.70, M21.721, M21.722, M21.729, M21.731, M21.732, M21.733, M21.734, M21.739, M21.751, M21.752, M21.759, M21.761, M21.762, M21.763, M21.764, M21.769, M21.80, M21.821, M21.822, M21.829, M21.831, M21.832, M21.839, M21.851, M21.852, M21.859, M21.861, M21.862, M21.869, M21.90, M21.921, M21.922, M21.929, M21.931, M21.932, M21.939, M21.941, M21.942, M21.949, M21.951, M21.952, M21.959, M21.961, M21.962, M21.969, M22.00, M22.01, M22.02, M22.10, M22.11, M22.12, M22.2X1, M22.2X2, M22.2X9, M22.3X1, M22.3X2, M22.3X9, M22.40, M22.41, M22.42, M22.8X1, M22.8X2, M22.8X9, M22.90, M22.91, M22.92, M23.000, M23.001, M23.002, M23.003, M23.004, M23.005, M23.006, M23.007, M23.009, M23.011, M23.012, M23.019, M23.021, M23.022, M23.029, M23.031, M23.032, M23.039, M23.041, M23.042, M23.049, M23.051, M23.052, M23.059, M23.061, M23.062, M23.069, M23.200, M23.201, M23.202, M23.203, M23.204, M23.205, M23.206, M23.207, M23.209, M23.211, M23.212, M23.219, M23.221, M23.222, M23.229, M23.231, M23.232, M23.239, M23.241, M23.242, M23.249, M23.251, M23.252, M23.259, M23.261, M23.262, M23.269, M23.300, M23.301, M23.302, M23.303, M23.304, M23.305, M23.306, M23.307, M23.309, M23.311, M23.312, M23.319, M23.321, M23.322, M23.329, M23.331, M23.332, M23.339, M23.341, M23.342, M23.349, M23.351, M23.352, M23.359, M23.361, M23.362, M23.369, M23.40, M23.41, M23.42, M23.50, M23.51, M23.52, M23.601, M23.602, M23.609, M23.611, M23.612, M23.619, M23.621, M23.622, M23.629, M23.631, M23.632, M23.639, M23.641, M23.642, M23.649, M23.671, M23.672, M23.679, M23.8X1, M23.8X2, M23.8X9, M23.90, M23.91, M23.92, M24.00, M24.011, M24.012, M24.019, M24.021, M24.022, M24.029, M24.031, M24.032, M24.039, M24.041, M24.042, M24.049, M24.051, M24.052, M24.059, M24.071, M24.072, M24.073, M24.074, M24.075, M24.076, M24.08, M24.10, M24.111, M24.112, M24.119, M24.121, M24.122, M24.129, M24.131, M24.132, M24.139, M24.141, M24.142, M24.149, M24.151, M24.152, M24.159, M24.171, M24.172, M24.173, M24.174, M24.175, M24.176, M24.20, M24.211, M24.212, M24.219, M24.221, M24.222, M24.229, M24.231, M24.232, M24.239, M24.241, M24.242, M24.249, M24.251, M24.252, M24.259, M24.271, M24.272, M24.273, M24.274, M24.275, M24.276, M24.28,

© 2025 Park EE et al. *JAMA Network Open*.

	718.21, 718.22,	M24.30, M24.311, M24.312, M24.319, M24.321, M24.322, M24.329, M24.331,
	718.23, 718.24,	M24.332, M24.339, M24.341, M24.342, M24.349, M24.351, M24.352, M24.359,
	718.25, 718.26,	M24.361, M24.362, M24.369, M24.371, M24.372, M24.373, M24.374, M24.375,
	718.27, 718.28,	M24.376, M24.40, M24.411, M24.412, M24.419, M24.421, M24.422, M24.429,
	718.29, 718.30,	M24.431, M24.432, M24.439, M24.441, M24.442, M24.443, M24.444, M24.445,
	718.31, 718.32,	M24.446, M24.451, M24.452, M24.459, M24.461, M24.462, M24.469, M24.471,
	718.33, 718.34,	M24.472, M24.473, M24.474, M24.475, M24.476, M24.477, M24.478, M24.479,
	718.35, 718.36,	M24.50, M24.511, M24.512, M24.519, M24.521, M24.522, M24.529, M24.531,
	718.37, 718.40,	M24.532, M24.539, M24.541, M24.542, M24.549, M24.551, M24.552, M24.559,
	718.41, 718.42,	M24.561, M24.562, M24.569, M24.571, M24.572, M24.573, M24.574, M24.575,
	718.43, 718.44,	M24.576, M24.60, M24.611, M24.612, M24.619, M24.621, M24.622, M24.629,
	718.45, 718.46,	M24.631, M24.632, M24.639, M24.641, M24.642, M24.649, M24.651, M24.652,
	718.47, 718.48,	M24.659, M24.661, M24.662, M24.669, M24.671, M24.672, M24.673, M24.674,
	718.49, 718.50,	M24.675, M24.676, M24.7, M24.80, M24.811, M24.812, M24.819, M24.821,
	718.51, 718.52,	M24.822, M24.829, M24.831, M24.832, M24.839, M24.841, M24.842, M24.849,
	718.53, 718.54,	M24.851, M24.852, M24.859, M24.871, M24.872, M24.873, M24.874, M24.875,
	718.55, 718.56,	M24.876, M24.9, M25.00, M25.011, M25.012, M25.019, M25.021, M25.022,
	718.57, 718.58,	M25.029, M25.031, M25.032, M25.039, M25.041, M25.042, M25.049, M25.051,
	718.59, 718.65,	M25.052, M25.059, M25.061, M25.062, M25.069, M25.071, M25.072, M25.073,
	718.70, 718.71,	M25.074, M25.075, M25.076, M25.08, M25.10, M25.111, M25.112, M25.119,
	718.72, 718.73,	M25.121, M25.122, M25.129, M25.131, M25.132, M25.139, M25.141, M25.142,
	718.74, 718.75,	M25.149, M25.151, M25.152, M25.159, M25.161, M25.162, M25.169, M25.171,
	718.76, 718.77,	M25.172, M25.173, M25.174, M25.175, M25.176, M25.18, M25.20, M25.211,
	718.78, 718.79,	M25.212, M25.219, M25.221, M25.222, M25.229, M25.231, M25.232, M25.239,
	718.80, 718.81,	M25.241, M25.242, M25.249, M25.251, M25.252, M25.259, M25.261, M25.262,
	718.82, 718.83,	M25.269, M25.271, M25.272, M25.279, M25.28, M25.30, M25.311, M25.312,
	718.84, 718.85,	M25.319, M25.321, M25.322, M25.329, M25.331, M25.332, M25.339, M25.341,
	718.86, 718.87,	M25.342, M25.349, M25.351, M25.352, M25.359, M25.361, M25.362, M25.369,
	718.88, 718.89,	M25.371, M25.372, M25.373, M25.374, M25.375, M25.376, M25.40, M25.411,
	718.90, 718.91,	M25.412, M25.419, M25.421, M25.422, M25.429, M25.431, M25.432, M25.439,
	718.92, 718.93,	M25.441, M25.442, M25.449, M25.451, M25.452, M25.459, M25.461, M25.462,
	718.94, 718.95,	M25.469, M25.471, M25.472, M25.473, M25.474, M25.475, M25.476, M25.48,
	718.97, 718.98,	M25.60, M25.611, M25.612, M25.619, M25.621, M25.622, M25.629, M25.631,
	718.99, 719.00,	M25.632, M25.639, M25.641, M25.642, M25.649, M25.651, M25.652, M25.659,
	719.01, 719.02,	M25.661, M25.662, M25.669, M25.671, M25.672, M25.673, M25.674, M25.675,
	719.03, 719.04,	M25.676, M25.70, M25.711, M25.712, M25.719, M25.721, M25.722, M25.729,
	719.05, 719.06,	M25.731, M25.732, M25.739, M25.741, M25.742, M25.749, M25.751, M25.752,
	719.07, 719.08,	M25.759, M25.761, M25.762, M25.769, M25.771, M25.772, M25.773, M25.774,
	719.09, 719.10,	M25.775, M25.776, M25.80, M25.811, M25.812, M25.819, M25.821, M25.822,
	719.11, 719.12,	M25.829, M25.831, M25.832, M25.839, M25.841, M25.842, M25.849, M25.851,
	719.13, 719.14,	M25.852, M25.859, M25.861, M25.862, M25.869, M25.871, M25.872, M25.879,
	719.15, 719.16,	M25.9, M26.601, M26.602, M26.603, M26.609, M26.611, M26.612, M26.613,
	719.17, 719.18,	M26.619, M26.621, M26.622, M26.623, M26.629, M26.631, M26.632, M26.633,
	719.19, 719.20,	M26.639, M26.69, M35.4, M35.7, M53.2X9, M60.10, M60.111, M60.112,
	719.21, 719.22,	M60.119, M60.121, M60.122, M60.129, M60.131, M60.132, M60.139, M60.141,
	719.23, 719.24,	M60.142, M60.149, M60.151, M60.152, M60.159, M60.161, M60.162, M60.169,
	719.25, 719.26,	M60.171, M60.172, M60.179, M60.18, M60.19, M60.20, M60.211, M60.212,
	719.27, 719.28,	M60.219, M60.221, M60.222, M60.229, M60.231, M60.232, M60.239, M60.241,
	719.29, 719.50,	M60.242, M60.249, M60.251, M60.252, M60.259, M60.261, M60.262, M60.269,
	719.51, 719.52,	M60.271, M60.272, M60.279, M60.28, M61.00, M61.011, M61.012, M61.019,
	719.53, 719.54,	M61.021, M61.022, M61.029, M61.031, M61.032, M61.039, M61.041, M61.042,
	719.55, 719.56,	M61.049, M61.051, M61.052, M61.059, M61.061, M61.062, M61.069, M61.071,
	719.57, 719.58,	M61.072, M61.079, M61.08, M61.09, M61.10, M61.111, M61.112, M61.119,
	719.59, 719.60,	M61.121, M61.122, M61.129, M61.131, M61.132, M61.139, M61.141, M61.142,
	719.61, 719.62,	M61.143, M61.144, M61.145, M61.146, M61.151, M61.152, M61.159, M61.161,
	719.63, 719.64,	M61.162, M61.169, M61.171, M61.172, M61.173, M61.174, M61.175, M61.176,
	719.65, 719.66,	M61.177, M61.178, M61.179, M61.18, M61.19, M61.20, M61.211, M61.212,
	719.67, 719.68,	M61.219, M61.221, M61.222, M61.229, M61.231, M61.232, M61.239, M61.241,
	719.69, 719.7,	M61.242, M61.249, M61.251, M61.252, M61.259, M61.261, M61.262, M61.269,
	719.80, 719.81,	M61.271, M61.272, M61.279, M61.28, M61.29, M61.30, M61.311, M61.312,

© 2025 Park EE et al. *JAMA Network Open*.

	719.82, 719.83, 719.84, 719.85, 719.86, 719.87, 719.88, 719.89, 719.90, 719.91, 719.92, 719.93, 719.94, 719.95, 719.96, 719.97, 719.98, 719.99, 726.0, 726.10, 726.11, 726.12, 726.13, 726.19, 726.2, 726.30, 726.31, 726.32, 726.33, 726.39, 726.4, 726.5, 726.60, 726.61, 726.62, 726.63, 726.64, 726.65, 726.69, 726.70, 726.71, 726.72, 726.73, 726.79, 726.8, 726.90, 726.91, 727.1, 727.2, 727.3, 727.40, 727.41, 727.42, 727.43, 727.49, 727.50, 727.51, 727.59, 727.60, 727.61, 727.62, 727.63, 727.64, 727.65, 727.66, 727.67, 727.68, 727.69, 727.81, 727.82, 727.83, 727.89, 727.9, 728.10, 728.11, 728.12, 728.13, 728.19, 728.2, 728.3, 728.4, 728.5, 728.6, 728.81, 728.82, 728.83, 728.84, 728.85, 728.86, 728.87, 728.88, 728.89, 728.9, 733.40, 733.41, 733.42, 733.43, 733.44, 733.45, 733.49, 733.5, 733.6, 733.7, 733.81, 733.82, 733.90, 733.91, 733.92, 733.99, 735.0, 735.1, 735.2, 735.3, 735.4, 735.5, 735.8, 735.9, 736.00, 736.01, 736.02,	M61.319, M61.321, M61.322, M61.329, M61.331, M61.332, M61.339, M61.341, M61.342, M61.349, M61.351, M61.352, M61.359, M61.361, M61.362, M61.369, M61.371, M61.372, M61.379, M61.38, M61.39, M61.40, M61.411, M61.412, M61.419, M61.421, M61.422, M61.429, M61.431, M61.432, M61.439, M61.441, M61.442, M61.449, M61.451, M61.452, M61.459, M61.461, M61.462, M61.469, M61.471, M61.472, M61.479, M61.48, M61.49, M61.50, M61.511, M61.512, M61.519, M61.521, M61.522, M61.529, M61.531, M61.532, M61.539, M61.541, M61.542, M61.549, M61.551, M61.552, M61.559, M61.561, M61.562, M61.569, M61.571, M61.572, M61.579, M61.58, M61.59, M61.9, M62.00, M62.011, M62.012, M62.019, M62.021, M62.022, M62.029, M62.031, M62.032, M62.039, M62.041, M62.042, M62.049, M62.051, M62.052, M62.059, M62.061, M62.062, M62.069, M62.071, M62.072, M62.079, M62.08, M62.10, M62.111, M62.112, M62.119, M62.121, M62.122, M62.129, M62.131, M62.132, M62.139, M62.141, M62.142, M62.149, M62.151, M62.152, M62.159, M62.161, M62.162, M62.169, M62.171, M62.172, M62.179, M62.18, M62.20, M62.211, M62.212, M62.219, M62.221, M62.222, M62.229, M62.231, M62.232, M62.239, M62.241, M62.242, M62.249, M62.251, M62.252, M62.259, M62.261, M62.262, M62.269, M62.271, M62.272, M62.279, M62.28, M62.40, M62.411, M62.412, M62.419, M62.421, M62.422, M62.429, M62.431, M62.432, M62.439, M62.441, M62.442, M62.449, M62.451, M62.452, M62.459, M62.461, M62.462, M62.469, M62.471, M62.472, M62.479, M62.48, M62.49, M62.50, M62.511, M62.512, M62.519, M62.521, M62.522, M62.529, M62.531, M62.532, M62.539, M62.541, M62.542, M62.549, M62.551, M62.552, M62.559, M62.561, M62.562, M62.569, M62.571, M62.572, M62.579, M62.58, M62.59, M62.81, M62.82, M62.831, M62.838, M62.84, M62.89, M62.9, M63.80, M63.811, M63.812, M63.819, M63.821, M63.822, M63.829, M63.831, M63.832, M63.839, M63.841, M63.842, M63.849, M63.851, M63.852, M63.859, M63.861, M63.862, M63.869, M63.871, M63.872, M63.879, M63.88, M63.89, M65.00, M65.011, M65.012, M65.019, M65.021, M65.022, M65.029, M65.031, M65.032, M65.039, M65.041, M65.042, M65.049, M65.051, M65.052, M65.059, M65.061, M65.062, M65.069, M65.071, M65.072, M65.079, M65.08, M65.20, M65.221, M65.222, M65.229, M65.231, M65.232, M65.239, M65.241, M65.242, M65.249, M65.251, M65.252, M65.259, M65.261, M65.262, M65.269, M65.271, M65.272, M65.279, M65.28, M65.29, M66.0, M66.10, M66.111, M66.112, M66.119, M66.121, M66.122, M66.129, M66.131, M66.132, M66.139, M66.141, M66.142, M66.143, M66.144, M66.145, M66.146, M66.151, M66.152, M66.159, M66.171, M66.172, M66.173, M66.174, M66.175, M66.176, M66.177, M66.178, M66.179, M66.18, M66.20, M66.211, M66.212, M66.219, M66.221, M66.222, M66.229, M66.231, M66.232, M66.239, M66.241, M66.242, M66.249, M66.251, M66.252, M66.259, M66.261, M66.262, M66.269, M66.271, M66.272, M66.279, M66.28, M66.29, M66.30, M66.311, M66.312, M66.319, M66.321, M66.322, M66.329, M66.331, M66.332, M66.339, M66.341, M66.342, M66.349, M66.351, M66.352, M66.359, M66.361, M66.362, M66.369, M66.371, M66.372, M66.379, M66.38, M66.39, M66.80, M66.811, M66.812, M66.819, M66.821, M66.822, M66.829, M66.831, M66.832, M66.839, M66.841, M66.842, M66.849, M66.851, M66.852, M66.859, M66.861, M66.862, M66.869, M66.871, M66.872, M66.879, M66.88, M66.89, M66.9, M67.00, M67.01, M67.02, M67.20, M67.211, M67.212, M67.219, M67.221, M67.222, M67.229, M67.231, M67.232, M67.239, M67.241, M67.242, M67.249, M67.251, M67.252, M67.259, M67.261, M67.262, M67.269, M67.271, M67.272, M67.279, M67.28, M67.29, M67.40, M67.411, M67.412, M67.419, M67.421, M67.422, M67.429, M67.431, M67.432, M67.439, M67.441, M67.442, M67.449, M67.451, M67.452, M67.459, M67.461, M67.462, M67.469, M67.471, M67.472, M67.479, M67.48, M67.49, M67.50, M67.51, M67.52, M67.80, M67.811, M67.812, M67.813, M67.814, M67.819, M67.821, M67.822, M67.823, M67.824, M67.829, M67.831, M67.832, M67.833, M67.834, M67.839, M67.841, M67.842, M67.843, M67.844, M67.849, M67.851, M67.852, M67.853, M67.854, M67.859, M67.861, M67.862, M67.863, M67.864, M67.869, M67.871, M67.872, M67.873, M67.874, M67.879, M67.88, M67.89, M67.90, M67.911, M67.912, M67.919, M67.921, M67.922, M67.929, M67.931, M67.932, M67.939, M67.941, M67.942, M67.949, M67.951, M67.952, M67.959, M67.961, M67.962, M67.969, M67.971, M67.972, M67.979, M67.98, M67.99,

© 2025 Park EE et al. *JAMA Network Open*.

	736.03, 736.04, 736.05, 736.06, 736.07, 736.09, 736.1, 736.20, 736.21, 736.22, 736.29, 736.30, 736.31, 736.32, 736.39, 736.41, 736.42, 736.5, 736.6, 736.70, 736.71, 736.72, 736.73, 736.74, 736.75, 736.76, 736.79, 736.81, 736.89, 736.9, 738.10, 738.11, 738.12, 738.19, 738.3, 738.8, 738.9, 739.0, 739.6, 739.7, 739.8	M70.031, M70.032, M70.039, M70.041, M70.042, M70.049, M70.10, M70.11, M70.12, M70.20, M70.21, M70.22, M70.30, M70.31, M70.32, M70.40, M70.41, M70.42, M70.50, M70.51, M70.52, M70.60, M70.61, M70.62, M70.70, M70.71, M70.72, M71.00, M71.011, M71.012, M71.019, M71.021, M71.022, M71.029, M71.031, M71.032, M71.039, M71.041, M71.042, M71.049, M71.051, M71.052, M71.059, M71.061, M71.062, M71.069, M71.071, M71.072, M71.079, M71.08, M71.09, M71.10, M71.111, M71.112, M71.119, M71.121, M71.122, M71.129, M71.131, M71.132, M71.139, M71.141, M71.142, M71.149, M71.151, M71.152, M71.159, M71.161, M71.162, M71.169, M71.171, M71.172, M71.179, M71.18, M71.19, M71.20, M71.21, M71.22, M71.30, M71.311, M71.312, M71.319, M71.321, M71.322, M71.329, M71.331, M71.332, M71.339, M71.341, M71.342, M71.349, M71.351, M71.352, M71.359, M71.371, M71.372, M71.379, M71.38, M71.39, M71.40, M71.421, M71.422, M71.429, M71.431, M71.432, M71.439, M71.441, M71.442, M71.449, M71.451, M71.452, M71.459, M71.461, M71.462, M71.469, M71.471, M71.472, M71.479, M71.48, M71.49, M71.50, M71.521, M71.522, M71.529, M71.531, M71.532, M71.539, M71.541, M71.542, M71.549, M71.551, M71.552, M71.559, M71.561, M71.562, M71.569, M71.571, M71.572, M71.579, M71.58, M71.80, M71.811, M71.812, M71.819, M71.821, M71.822, M71.829, M71.831, M71.832, M71.839, M71.841, M71.842, M71.849, M71.851, M71.852, M71.859, M71.861, M71.862, M71.869, M71.871, M71.872, M71.879, M71.88, M71.89, M71.9, M72.0, M72.6, M75.00, M75.01, M75.02, M75.100, M75.101, M75.102, M75.110, M75.111, M75.112, M75.120, M75.121, M75.122, M75.20, M75.21, M75.22, M75.30, M75.31, M75.32, M75.40, M75.41, M75.42, M75.50, M75.51, M75.52, M75.80, M75.81, M75.82, M75.90, M75.91, M75.92, M76.00, M76.01, M76.02, M76.10, M76.11, M76.12, M76.20, M76.21, M76.22, M76.30, M76.31, M76.32, M76.40, M76.41, M76.42, M76.50, M76.51, M76.52, M76.60, M76.61, M76.62, M76.70, M76.71, M76.72, M76.811, M76.812, M76.819, M76.821, M76.822, M76.829, M76.891, M76.892, M76.899, M76.9, M77.00, M77.01, M77.02, M77.10, M77.11, M77.12, M77.20, M77.21, M77.22, M77.30, M77.31, M77.32, M77.40, M77.41, M77.42, M77.50, M77.51, M77.52, M77.8, M77.9, M80.00XK, M80.00XP, M80.011K, M80.011P, M80.012K, M80.012P, M80.019K, M80.019P, M80.021K, M80.021P, M80.022K, M80.022P, M80.029K, M80.029P, M80.031K, M80.031P, M80.032K, M80.032P, M80.039K, M80.039P, M80.041K, M80.041P, M80.042K, M80.042P, M80.049K, M80.049P, M80.051K, M80.051P, M80.052K, M80.052P, M80.059K, M80.059P, M80.061K, M80.061P, M80.062K, M80.062P, M80.069K, M80.069P, M80.071K, M80.071P, M80.072K, M80.072P, M80.079K, M80.079P, M80.08XK, M80.08XP, M80.80XK, M80.80XP, M80.811K, M80.811P, M80.812K, M80.812P, M80.819K, M80.819P, M80.821K, M80.821P, M80.822K, M80.822P, M80.829K, M80.829P, M80.831K, M80.831P, M80.832K, M80.832P, M80.839K, M80.839P, M80.841K, M80.841P, M80.842K, M80.842P, M80.849K, M80.849P, M80.851K, M80.851P, M80.852K, M80.852P, M80.859K, M80.859P, M80.861K, M80.861P, M80.862K, M80.862P, M80.869K, M80.869P, M80.871K, M80.871P, M80.872K, M80.872P, M80.879K, M80.879P, M80.88XK, M80.88XP, M84.30XK, M84.30XP, M84.311K, M84.311P, M84.312K, M84.312P, M84.319K, M84.319P, M84.321K, M84.321P, M84.322K, M84.322P, M84.329K, M84.329P, M84.331K, M84.331P, M84.332K, M84.332P, M84.333K, M84.333P, M84.334K, M84.334P, M84.339K, M84.339P, M84.341K, M84.341P, M84.342K, M84.342P, M84.343K, M84.343P, M84.344K, M84.344P, M84.345K, M84.345P, M84.346K, M84.346P, M84.350K, M84.350P, M84.351K, M84.351P, M84.352K, M84.352P, M84.353K, M84.353P, M84.359K, M84.359P, M84.361K, M84.361P, M84.362K, M84.362P, M84.363K, M84.363P, M84.364K, M84.364P, M84.369K, M84.369P, M84.371K, M84.371P, M84.372K, M84.372P, M84.373K, M84.373P, M84.374K, M84.374P, M84.375K, M84.375P, M84.376K, M84.376P, M84.377K, M84.377P, M84.378K, M84.378P, M84.379K, M84.379P, M84.38XK, M84.38XP, M84.40XK, M84.40XP, M84.411K, M84.411P, M84.412K, M84.412P, M84.419K, M84.419P, M84.421K, M84.421P, M84.422K, M84.422P, M84.429K, M84.429P, M84.431K, M84.431P, M84.432K, M84.432P, M84.433K, M84.433P, M84.434K, M84.434P, M84.439K, M84.439P, M84.441K, M84.441P, M84.442K, M84.442P, M84.443K, M84.443P, M84.444K, M84.444P, M84.445K, M84.445P, M84.446K, M84.446P, M84.451K, M84.451P, M84.452K, M84.452P,

© 2025 Park EE et al. *JAMA Network Open*.

		M84.453K, M84.453P, M84.454K, M84.454P, M84.459K, M84.459P, M84.461K, M84.461P, M84.462K, M84.462P, M84.463K, M84.463P, M84.464K, M84.464P, M84.469K, M84.469P, M84.471K, M84.471P, M84.472K, M84.472P, M84.473K, M84.473P, M84.474K, M84.474P, M84.475K, M84.475P, M84.476K, M84.476P, M84.477K, M84.477P, M84.478K, M84.478P, M84.479K, M84.479P, M84.48XK, M84.48XP, M84.50XK, M84.50XP, M84.511K, M84.511P, M84.512K, M84.512P, M84.519K, M84.519P, M84.521K, M84.521P, M84.522K, M84.522P, M84.529K, M84.529P, M84.531K, M84.531P, M84.532K, M84.532P, M84.533K, M84.533P, M84.534K, M84.534P, M84.539K, M84.539P, M84.541K, M84.541P, M84.542K, M84.542P, M84.549K, M84.549P, M84.550K, M84.550P, M84.551K, M84.551P, M84.552K, M84.552P, M84.553K, M84.553P, M84.559K, M84.559P, M84.561K, M84.561P, M84.562K, M84.562P, M84.563K, M84.563P, M84.564K, M84.564P, M84.569K, M84.569P, M84.571K, M84.571P, M84.572K, M84.572P, M84.573K, M84.573P, M84.574K, M84.574P, M84.575K, M84.575P, M84.576K, M84.576P, M84.58XK, M84.58XP, M84.60XK, M84.60XP, M84.611K, M84.611P, M84.612K, M84.612P, M84.619K, M84.619P, M84.621K, M84.621P, M84.622K, M84.622P, M84.629K, M84.629P, M84.631K, M84.631P, M84.632K, M84.632P, M84.633K, M84.633P, M84.634K, M84.634P, M84.639K, M84.639P, M84.641K, M84.641P, M84.642K, M84.642P, M84.649K, M84.649P, M84.650K, M84.650P, M84.651K, M84.651P, M84.652K, M84.652P, M84.653K, M84.653P, M84.659K, M84.659P, M84.661K, M84.661P, M84.662K, M84.662P, M84.663K, M84.663P, M84.664K, M84.664P, M84.669K, M84.669P, M84.671K, M84.671P, M84.672K, M84.672P, M84.673K, M84.673P, M84.674K, M84.674P, M84.675K, M84.675P, M84.676K, M84.676P, M84.68XK, M84.68XP, M84.750K, M84.750P, M84.751K, M84.751P, M84.752K, M84.752P, M84.753K, M84.753P, M84.754K, M84.754P, M84.755K, M84.755P, M84.756K, M84.756P, M84.757K, M84.757P, M84.758K, M84.758P, M84.759K, M84.759P, M84.80, M84.811, M84.812, M84.819, M84.821, M84.822, M84.829, M84.831, M84.832, M84.833, M84.834, M84.839, M84.841, M84.842, M84.849, M84.851, M84.852, M84.859, M84.861, M84.862, M84.863, M84.864, M84.869, M84.871, M84.872, M84.879, M84.88, M84.9, M85.10, M85.111, M85.112, M85.119, M85.121, M85.122, M85.129, M85.131, M85.132, M85.139, M85.141, M85.142, M85.149, M85.151, M85.152, M85.159, M85.161, M85.162, M85.169, M85.171, M85.172, M85.179, M85.18, M85.19, M85.30, M85.311, M85.312, M85.319, M85.321, M85.322, M85.329, M85.331, M85.332, M85.339, M85.341, M85.342, M85.349, M85.351, M85.352, M85.359, M85.361, M85.362, M85.369, M85.371, M85.372, M85.379, M85.38, M85.39, M85.80, M85.811, M85.812, M85.819, M85.821, M85.822, M85.829, M85.831, M85.832, M85.839, M85.841, M85.842, M85.849, M85.851, M85.852, M85.859, M85.861, M85.862, M85.869, M85.871, M85.872, M85.879, M85.88, M85.89, M85.9, M87.00, M87.011, M87.012, M87.019, M87.021, M87.022, M87.029, M87.031, M87.032, M87.033, M87.034, M87.035, M87.036, M87.037, M87.038, M87.039, M87.041, M87.042, M87.043, M87.044, M87.045, M87.046, M87.050, M87.051, M87.052, M87.059, M87.061, M87.062, M87.063, M87.064, M87.065, M87.066, M87.071, M87.072, M87.073, M87.074, M87.075, M87.076, M87.077, M87.078, M87.079, M87.08, M87.09, M87.10, M87.111, M87.112, M87.119, M87.121, M87.122, M87.129, M87.131, M87.132, M87.133, M87.134, M87.135, M87.136, M87.137, M87.138, M87.139, M87.141, M87.142, M87.143, M87.144, M87.145, M87.146, M87.150, M87.151, M87.152, M87.159, M87.161, M87.162, M87.163, M87.164, M87.165, M87.166, M87.171, M87.172, M87.173, M87.174, M87.175, M87.176, M87.177, M87.178, M87.179, M87.180, M87.188, M87.19, M87.20, M87.211, M87.212, M87.219, M87.221, M87.222, M87.229, M87.231, M87.232, M87.233, M87.234, M87.235, M87.236, M87.237, M87.238, M87.239, M87.241, M87.242, M87.243, M87.244, M87.245, M87.246, M87.250, M87.251, M87.252, M87.256, M87.261, M87.262, M87.263, M87.264, M87.265, M87.266, M87.271, M87.272, M87.273, M87.274, M87.275, M87.276, M87.277, M87.278, M87.279, M87.28, M87.29, M87.30, M87.311, M87.312, M87.319, M87.321, M87.322, M87.329, M87.331, M87.332, M87.333, M87.334, M87.335, M87.336, M87.337, M87.338, M87.339, M87.341, M87.342, M87.343, M87.344, M87.345, M87.346, M87.350, M87.351, M87.352, M87.353, M87.361, M87.362, M87.363, M87.364, M87.365, M87.366, M87.371, M87.372, M87.373, M87.374, M87.375, M87.376, M87.377,

© 2025 Park EE et al. *JAMA Network Open*.

		M87.378, M87.379, M87.38, M87.39, M87.80, M87.811, M87.812, M87.819, M87.821, M87.822, M87.829, M87.831, M87.832, M87.833, M87.834, M87.835, M87.836, M87.837, M87.838, M87.839, M87.841, M87.842, M87.843, M87.844, M87.845, M87.849, M87.850, M87.851, M87.852, M87.859, M87.861, M87.862, M87.863, M87.864, M87.865, M87.869, M87.871, M87.872, M87.873, M87.874, M87.875, M87.876, M87.877, M87.878, M87.879, M87.88, M87.89, M87.9, M89.00, M89.011, M89.012, M89.019, M89.021, M89.022, M89.029, M89.031, M89.032, M89.039, M89.041, M89.042, M89.049, M89.051, M89.052, M89.059, M89.061, M89.062, M89.069, M89.071, M89.072, M89.079, M89.08, M89.09, M89.121, M89.122, M89.123, M89.124, M89.125, M89.126, M89.127, M89.128, M89.129, M89.131, M89.132, M89.133, M89.134, M89.138, M89.139, M89.151, M89.152, M89.153, M89.154, M89.155, M89.156, M89.157, M89.158, M89.159, M89.160, M89.161, M89.162, M89.163, M89.164, M89.165, M89.166, M89.167, M89.168, M89.169, M89.18, M89.20, M89.211, M89.212, M89.219, M89.221, M89.222, M89.229, M89.231, M89.232, M89.233, M89.234, M89.239, M89.241, M89.242, M89.249, M89.251, M89.252, M89.259, M89.261, M89.262, M89.263, M89.264, M89.269, M89.271, M89.272, M89.279, M89.28, M89.29, M89.30, M89.311, M89.312, M89.319, M89.321, M89.322, M89.329, M89.331, M89.332, M89.333, M89.334, M89.339, M89.341, M89.342, M89.349, M89.351, M89.352, M89.359, M89.361, M89.362, M89.363, M89.364, M89.369, M89.371, M89.372, M89.379, M89.38, M89.39, M89.50, M89.511, M89.512, M89.519, M89.521, M89.522, M89.529, M89.531, M89.532, M89.539, M89.541, M89.542, M89.549, M89.551, M89.552, M89.559, M89.561, M89.562, M89.569, M89.571, M89.572, M89.579, M89.58, M89.59, M89.8X0, M89.8X1, M89.8X2, M89.8X3, M89.8X4, M89.8X5, M89.8X6, M89.8X7, M89.8X8, M89.8X9, M89.9, M90.50, M90.511, M90.512, M90.519, M90.521, M90.522, M90.529, M90.531, M90.532, M90.539, M90.541, M90.542, M90.549, M90.551, M90.552, M90.559, M90.561, M90.562, M90.569, M90.571, M90.572, M90.579, M90.58, M90.59, M94.0, M94.1, M94.20, M94.211, M94.212, M94.219, M94.221, M94.222, M94.229, M94.231, M94.232, M94.239, M94.241, M94.242, M94.249, M94.251, M94.252, M94.259, M94.261, M94.262, M94.269, M94.271, M94.272, M94.279, M94.28, M94.29, M94.351, M94.352, M94.359, M94.8X0, M94.8X1, M94.8X2, M94.8X3, M94.8X4, M94.8X5, M94.8X6, M94.8X7, M94.8X8, M94.8X9, M94.9, M95.2, M95.4, M95.8, M95.9, M99.00, M99.06, M99.07, M99.08, M99.80, M99.82, M99.86, M99.87, M99.88, M99.89, M99.9, N64.4, Q68.6, R26.2, R29.4, R29.898, S02.0XXK, S02.101K, S02.102K, S02.109K, S02.110K, S02.111K, S02.112K, S02.113K, S02.118K, S02.119K, S02.11AK, S02.11BK, S02.11CK, S02.11DK, S02.11EK, S02.11FK, S02.11GK, S02.11HK, S02.19XK, S02.2XXK, S02.30XK, S02.31XK, S02.32XK, S02.400K, S02.401K, S02.402K, S02.40AK, S02.40BK, S02.40CK, S02.40DK, S02.40EK, S02.40FK, S02.411K, S02.412K, S02.413K, S02.42XK, S02.5XXK, S02.600K, S02.601K, S02.602K, S02.609K, S02.610K, S02.611K, S02.612K, S02.620K, S02.621K, S02.622K, S02.630K, S02.631K, S02.632K, S02.640K, S02.641K, S02.642K, S02.650K, S02.651K, S02.652K, S02.66XK, S02.670K, S02.671K, S02.672K, S02.69XK, S02.80XK, S02.81XK, S02.82XK, S02.91XK, S02.92XK, S12.000K, S12.001K, S12.01XK, S12.02XK, S12.030K, S12.031K, S12.040K, S12.041K, S12.090K, S12.091K, S12.100K, S12.101K, S12.110K, S12.111K, S12.112K, S12.120K, S12.121K, S12.130K, S12.131K, S12.14XK, S12.150K, S12.151K, S12.190K, S12.191K, S12.200K, S12.201K, S12.230K, S12.231K, S12.24XK, S12.250K, S12.251K, S12.290K, S12.291K, S12.300K, S12.301K, S12.330K, S12.331K, S12.34XK, S12.350K, S12.351K, S12.390K, S12.391K, S12.400K, S12.401K, S12.430K, S12.431K, S12.44XK, S12.450K, S12.451K, S12.490K, S12.491K, S12.500K, S12.501K, S12.530K, S12.531K, S12.54XK, S12.550K, S12.551K, S12.590K, S12.591K, S12.600K, S12.601K, S12.630K, S12.631K, S12.64XK, S12.650K, S12.651K, S12.690K, S12.691K, S22.000K, S22.001K, S22.002K, S22.008K, S22.009K, S22.010K, S22.011K, S22.012K, S22.018K, S22.019K, S22.020K, S22.021K, S22.022K, S22.028K, S22.029K, S22.030K, S22.031K, S22.032K, S22.038K, S22.039K, S22.040K, S22.041K, S22.042K, S22.048K, S22.049K, S22.050K, S22.051K, S22.052K, S22.058K, S22.059K, S22.060K, S22.061K, S22.062K, S22.068K, S22.069K, S22.070K, S22.071K, S22.072K, S22.078K, S22.079K, S22.080K, S22.081K,

© 2025 Park EE et al. *JAMA Network Open*.

		S22.082K, S22.088K, S22.089K, S22.20XK, S22.21XK, S22.22XK, S22.23XK, S22.24XK, S22.31XK, S22.32XK, S22.39XK, S22.41XK, S22.42XK, S22.43XK, S22.49XK, S22.5XXK, S22.9XXK, S32.000K, S32.001K, S32.002K, S32.008K, S32.009K, S32.010K, S32.011K, S32.012K, S32.018K, S32.019K, S32.020K, S32.021K, S32.022K, S32.028K, S32.029K, S32.030K, S32.031K, S32.032K, S32.038K, S32.039K, S32.040K, S32.041K, S32.042K, S32.048K, S32.049K, S32.050K, S32.051K, S32.052K, S32.058K, S32.059K, S32.10XK, S32.110K, S32.111K, S32.112K, S32.119K, S32.120K, S32.121K, S32.122K, S32.129K, S32.130K, S32.131K, S32.132K, S32.139K, S32.14XK, S32.15XK, S32.16XK, S32.17XK, S32.19XK, S32.2XXK, S32.301K, S32.302K, S32.309K, S32.311K, S32.312K, S32.313K, S32.314K, S32.315K, S32.316K, S32.391K, S32.392K, S32.399K, S32.401K, S32.402K, S32.409K, S32.411K, S32.412K, S32.413K, S32.414K, S32.415K, S32.416K, S32.421K, S32.422K, S32.423K, S32.424K, S32.425K, S32.426K, S32.431K, S32.432K, S32.433K, S32.434K, S32.435K, S32.436K, S32.441K, S32.442K, S32.443K, S32.444K, S32.445K, S32.446K, S32.451K, S32.452K, S32.453K, S32.454K, S32.455K, S32.456K, S32.461K, S32.462K, S32.463K, S32.464K, S32.465K, S32.466K, S32.471K, S32.472K, S32.473K, S32.474K, S32.475K, S32.476K, S32.481K, S32.482K, S32.483K, S32.484K, S32.485K, S32.486K, S32.491K, S32.492K, S32.499K, S32.501K, S32.502K, S32.509K, S32.511K, S32.512K, S32.519K, S32.591K, S32.592K, S32.599K, S32.601K, S32.602K, S32.609K, S32.611K, S32.612K, S32.613K, S32.614K, S32.615K, S32.616K, S32.691K, S32.692K, S32.699K, S32.810K, S32.811K, S32.82XK, S32.89XK, S32.9XXK, S42.001K, S42.001P, S42.002K, S42.002P, S42.009K, S42.009P, S42.011K, S42.011P, S42.012K, S42.012P, S42.013K, S42.013P, S42.014K, S42.014P, S42.015K, S42.015P, S42.016K, S42.016P, S42.017K, S42.017P, S42.018K, S42.018P, S42.019K, S42.019P, S42.021K, S42.021P, S42.022K, S42.022P, S42.023K, S42.023P, S42.024K, S42.024P, S42.025K, S42.025P, S42.026K, S42.026P, S42.031K, S42.031P, S42.032K, S42.032P, S42.033K, S42.033P, S42.034K, S42.034P, S42.035K, S42.035P, S42.036K, S42.036P, S42.101K, S42.101P, S42.102K, S42.102P, S42.109K, S42.109P, S42.111K, S42.111P, S42.112K, S42.112P, S42.113K, S42.113P, S42.114K, S42.114P, S42.115K, S42.115P, S42.116K, S42.116P, S42.121K, S42.121P, S42.122K, S42.122P, S42.123K, S42.123P, S42.124K, S42.124P, S42.125K, S42.125P, S42.126K, S42.126P, S42.131K, S42.131P, S42.132K, S42.132P, S42.133K, S42.133P, S42.134K, S42.134P, S42.135K, S42.135P, S42.136K, S42.136P, S42.141K, S42.141P, S42.142K, S42.142P, S42.143K, S42.143P, S42.144K, S42.144P, S42.145K, S42.145P, S42.146K, S42.146P, S42.151K, S42.151P, S42.152K, S42.152P, S42.153K, S42.153P, S42.154K, S42.154P, S42.155K, S42.155P, S42.156K, S42.156P, S42.191K, S42.191P, S42.192K, S42.192P, S42.199K, S42.199P, S42.201K, S42.201P, S42.202K, S42.202P, S42.209K, S42.209P, S42.211K, S42.211P, S42.212K, S42.212P, S42.213K, S42.213P, S42.214K, S42.214P, S42.215K, S42.215P, S42.216K, S42.216P, S42.221K, S42.221P, S42.222K, S42.222P, S42.223K, S42.223P, S42.224K, S42.224P, S42.225K, S42.225P, S42.226K, S42.226P, S42.231K, S42.231P, S42.232K, S42.232P, S42.239K, S42.239P, S42.241K, S42.241P, S42.242K, S42.242P, S42.249K, S42.249P, S42.251K, S42.251P, S42.252K, S42.252P, S42.253K, S42.253P, S42.254K, S42.254P, S42.255K, S42.255P, S42.256K, S42.256P, S42.261K, S42.261P, S42.262K, S42.262P, S42.263K, S42.263P, S42.264K, S42.264P, S42.265K, S42.265P, S42.266K, S42.266P, S42.271K, S42.271P, S42.272K, S42.272P, S42.279K, S42.279P, S42.291K, S42.291P, S42.292K, S42.292P, S42.293K, S42.293P, S42.294K, S42.294P, S42.295K, S42.295P, S42.296K, S42.296P, S42.301K, S42.301P, S42.302K, S42.302P, S42.309K, S42.309P, S42.311K, S42.311P, S42.312K, S42.312P, S42.319K, S42.319P, S42.321K, S42.321P, S42.322K, S42.322P, S42.323K, S42.323P, S42.324K, S42.324P, S42.325K, S42.325P, S42.326K, S42.326P, S42.331K, S42.331P, S42.332K, S42.332P, S42.333K, S42.333P, S42.334K, S42.334P, S42.335K, S42.335P, S42.336K, S42.336P, S42.341K, S42.341P, S42.342K, S42.342P, S42.343K, S42.343P, S42.344K, S42.344P, S42.345K, S42.345P, S42.346K, S42.346P, S42.351K, S42.351P, S42.352K, S42.352P, S42.353K, S42.353P, S42.354K, S42.354P, S42.355K, S42.355P,

© 2025 Park EE et al. *JAMA Network Open*.

		S42.356K, S42.356P, S42.361K, S42.361P, S42.362K, S42.362P, S42.363K, S42.363P, S42.364K, S42.364P, S42.365K, S42.365P, S42.366K, S42.366P, S42.391K, S42.391P, S42.392K, S42.392P, S42.399K, S42.399P, S42.401K, S42.401P, S42.402K, S42.402P, S42.409K, S42.409P, S42.411K, S42.411P, S42.412K, S42.412P, S42.413K, S42.413P, S42.414K, S42.414P, S42.415K, S42.415P, S42.416K, S42.416P, S42.421K, S42.421P, S42.422K, S42.422P, S42.423K, S42.423P, S42.424K, S42.424P, S42.425K, S42.425P, S42.426K, S42.426P, S42.431K, S42.431P, S42.432K, S42.432P, S42.433K, S42.433P, S42.434K, S42.434P, S42.435K, S42.435P, S42.436K, S42.436P, S42.441K, S42.441P, S42.442K, S42.442P, S42.443K, S42.443P, S42.444K, S42.444P, S42.445K, S42.445P, S42.446K, S42.446P, S42.447K, S42.447P, S42.448K, S42.448P, S42.449K, S42.449P, S42.451K, S42.451P, S42.452K, S42.452P, S42.453K, S42.453P, S42.454K, S42.454P, S42.455K, S42.455P, S42.456K, S42.456P, S42.461K, S42.461P, S42.462K, S42.462P, S42.463K, S42.463P, S42.464K, S42.464P, S42.465K, S42.465P, S42.466K, S42.466P, S42.471K, S42.471P, S42.472K, S42.472P, S42.473K, S42.473P, S42.474K, S42.474P, S42.475K, S42.475P, S42.476K, S42.476P, S42.481K, S42.481P, S42.482K, S42.482P, S42.489K, S42.489P, S42.491K, S42.491P, S42.492K, S42.492P, S42.493K, S42.493P, S42.494K, S42.494P, S42.495K, S42.495P, S42.496K, S42.496P, S42.90XK, S42.90XP, S42.91XK, S42.91XP, S42.92XK, S42.92XP, S49.001K, S49.001P, S49.002K, S49.002P, S49.009K, S49.009P, S49.011K, S49.011P, S49.012K, S49.012P, S49.019K, S49.019P, S49.021K, S49.021P, S49.022K, S49.022P, S49.029K, S49.029P, S49.031K, S49.031P, S49.032K, S49.032P, S49.039K, S49.039P, S49.041K, S49.041P, S49.042K, S49.042P, S49.049K, S49.049P, S49.091K, S49.091P, S49.092K, S49.092P, S49.099K, S49.099P, S49.101K, S49.101P, S49.102K, S49.102P, S49.109K, S49.109P, S49.111K, S49.111P, S49.112K, S49.112P, S49.119K, S49.119P, S49.121K, S49.121P, S49.122K, S49.122P, S49.129K, S49.129P, S49.131K, S49.131P, S49.132K, S49.132P, S49.139K, S49.139P, S49.141K, S49.141P, S49.142K, S49.142P, S49.149K, S49.149P, S49.191K, S49.191P, S49.192K, S49.192P, S49.199K, S49.199P, S52.001K, S52.001M, S52.001N, S52.001P, S52.001Q, S52.001R, S52.002K, S52.002M, S52.002N, S52.002P, S52.002Q, S52.002R, S52.009K, S52.009M, S52.009N, S52.009P, S52.009Q, S52.009R, S52.011K, S52.011P, S52.012K, S52.012P, S52.019K, S52.019P, S52.021K, S52.021M, S52.021N, S52.021P, S52.021Q, S52.021R, S52.022K, S52.022M, S52.022N, S52.022P, S52.022Q, S52.022R, S52.023K, S52.023M, S52.023N, S52.023P, S52.023Q, S52.023R, S52.024K, S52.024M, S52.024N, S52.024P, S52.024Q, S52.024R, S52.025K, S52.025M, S52.025N, S52.025P, S52.025Q, S52.025R, S52.026K, S52.026M, S52.026N, S52.026P, S52.026Q, S52.026R, S52.031K, S52.031M, S52.031N, S52.031P, S52.031Q, S52.031R, S52.032K, S52.032M, S52.032N, S52.032P, S52.032Q, S52.032R, S52.033K, S52.033M, S52.033N, S52.033P, S52.033Q, S52.033R, S52.034K, S52.034M, S52.034N, S52.034P, S52.034Q, S52.034R, S52.035K, S52.035M, S52.035N, S52.035P, S52.035Q, S52.035R, S52.036K, S52.036M, S52.036N, S52.036P, S52.036Q, S52.036R, S52.041K, S52.041M, S52.041N, S52.041P, S52.041Q, S52.041R, S52.042K, S52.042M, S52.042N, S52.042P, S52.042Q, S52.042R, S52.043K, S52.043M, S52.043N, S52.043P, S52.043Q, S52.043R, S52.044K, S52.044M, S52.044N, S52.044P, S52.044Q, S52.044R, S52.045K, S52.045M, S52.045N, S52.045P, S52.045Q, S52.045R, S52.046K, S52.046M, S52.046N, S52.046P, S52.046Q, S52.046R, S52.091K, S52.091M, S52.091N, S52.091P, S52.091Q, S52.091R, S52.092K, S52.092M, S52.092N, S52.092P, S52.092Q, S52.092R, S52.099K, S52.099M, S52.099N, S52.099P, S52.099Q, S52.099R, S52.101K, S52.101M, S52.101N, S52.101P, S52.101Q, S52.101R, S52.102K, S52.102M, S52.102N, S52.102P, S52.102Q, S52.102R, S52.109K, S52.109M, S52.109N, S52.109P, S52.109Q, S52.109R, S52.111K, S52.111P, S52.112K, S52.112P, S52.119K, S52.119P, S52.121K, S52.121M, S52.121N, S52.121P, S52.121Q, S52.121R, S52.122K, S52.122M, S52.122N, S52.122P, S52.122Q, S52.122R, S52.123K, S52.123M, S52.123N, S52.123P, S52.123Q, S52.123R, S52.124K, S52.124M, S52.124N, S52.124P, S52.124Q, S52.124R, S52.125K, S52.125M, S52.125N, S52.125P, S52.125Q, S52.125R, S52.126K, S52.126M, S52.126N, S52.126P,

© 2025 Park EE et al. *JAMA Network Open*.

		S52.126Q, S52.126R, S52.131K, S52.131M, S52.131N, S52.131P, S52.131Q, S52.131R, S52.132K, S52.132M, S52.132N, S52.132P, S52.132Q, S52.132R, S52.133K, S52.133M, S52.133N, S52.133P, S52.133Q, S52.133R, S52.134K, S52.134M, S52.134N, S52.134P, S52.134Q, S52.134R, S52.135K, S52.135M, S52.135N, S52.135P, S52.135Q, S52.135R, S52.136K, S52.136M, S52.136N, S52.136P, S52.136Q, S52.136R, S52.181K, S52.181M, S52.181N, S52.181P, S52.181Q, S52.181R, S52.182K, S52.182M, S52.182N, S52.182P, S52.182Q, S52.182R, S52.189K, S52.189M, S52.189N, S52.189P, S52.189Q, S52.189R, S52.201K, S52.201M, S52.201N, S52.201P, S52.201Q, S52.201R, S52.202K, S52.202M, S52.202N, S52.202P, S52.202Q, S52.202R, S52.209K, S52.209M, S52.209N, S52.209P, S52.209Q, S52.209R, S52.211K, S52.211P, S52.212K, S52.212P, S52.219K, S52.219P, S52.221K, S52.221M, S52.221N, S52.221P, S52.221Q, S52.221R, S52.222K, S52.222M, S52.222N, S52.222P, S52.222Q, S52.222R, S52.223K, S52.223M, S52.223N, S52.223P, S52.223Q, S52.223R, S52.224K, S52.224M, S52.224N, S52.224P, S52.224Q, S52.224R, S52.225K, S52.225M, S52.225N, S52.225P, S52.225Q, S52.225R, S52.226K, S52.226M, S52.226N, S52.226P, S52.226Q, S52.226R, S52.231K, S52.231M, S52.231N, S52.231P, S52.231Q, S52.231R, S52.232K, S52.232M, S52.232N, S52.232P, S52.232Q, S52.232R, S52.233K, S52.233M, S52.233N, S52.233P, S52.233Q, S52.233R, S52.234K, S52.234M, S52.234N, S52.234P, S52.234Q, S52.234R, S52.235K, S52.235M, S52.235N, S52.235P, S52.235Q, S52.235R, S52.236K, S52.236M, S52.236N, S52.236P, S52.236Q, S52.236R, S52.241K, S52.241M, S52.241N, S52.241P, S52.241Q, S52.241R, S52.242K, S52.242M, S52.242N, S52.242P, S52.242Q, S52.242R, S52.243K, S52.243M, S52.243N, S52.243P, S52.243Q, S52.243R, S52.244K, S52.244M, S52.244N, S52.244P, S52.244Q, S52.244R, S52.245K, S52.245M, S52.245N, S52.245P, S52.245Q, S52.245R, S52.246K, S52.246M, S52.246N, S52.246P, S52.246Q, S52.246R, S52.251K, S52.251M, S52.251N, S52.251P, S52.251Q, S52.251R, S52.252K, S52.252M, S52.252N, S52.252P, S52.252Q, S52.252R, S52.253K, S52.253M, S52.253N, S52.253P, S52.253Q, S52.253R, S52.254K, S52.254M, S52.254N, S52.254P, S52.254Q, S52.254R, S52.255K, S52.255M, S52.255N, S52.255P, S52.255Q, S52.255R, S52.256K, S52.256M, S52.256N, S52.256P, S52.256Q, S52.256R, S52.261K, S52.261M, S52.261N, S52.261P, S52.261Q, S52.261R, S52.262K, S52.262M, S52.262N, S52.262P, S52.262Q, S52.262R, S52.263K, S52.263M, S52.263N, S52.263P, S52.263Q, S52.263R, S52.264K, S52.264M, S52.264N, S52.264P, S52.264Q, S52.264R, S52.265K, S52.265M, S52.265N, S52.265P, S52.265Q, S52.265R, S52.266K, S52.266M, S52.266N, S52.266P, S52.266Q, S52.266R, S52.271K, S52.271M, S52.271N, S52.271P, S52.271Q, S52.271R, S52.272K, S52.272M, S52.272N, S52.272P, S52.272Q, S52.272R, S52.279K, S52.279M, S52.279N, S52.279P, S52.279Q, S52.279R, S52.281K, S52.281M, S52.281N, S52.281P, S52.281Q, S52.281R, S52.282K, S52.282M, S52.282N, S52.282P, S52.282Q, S52.282R, S52.283K, S52.283M, S52.283N, S52.283P, S52.283Q, S52.283R, S52.291K, S52.291M, S52.291N, S52.291P, S52.291Q, S52.291R, S52.292K, S52.292M, S52.292N, S52.292P, S52.292Q, S52.292R, S52.299K, S52.299M, S52.299N, S52.299P, S52.299Q, S52.299R, S52.301K, S52.301M, S52.301N, S52.301P, S52.301Q, S52.301R, S52.302K, S52.302M, S52.302N, S52.302P, S52.302Q, S52.302R, S52.309K, S52.309M, S52.309N, S52.309P, S52.309Q, S52.309R, S52.311K, S52.311P, S52.312K, S52.312P, S52.319K, S52.319P, S52.321K, S52.321M, S52.321N, S52.321P, S52.321Q, S52.321R, S52.322K, S52.322M, S52.322N, S52.322P, S52.322Q, S52.322R, S52.323K, S52.323M, S52.323N, S52.323P, S52.323Q, S52.323R, S52.324K, S52.324M, S52.324N, S52.324P, S52.324Q, S52.324R, S52.325K, S52.325M, S52.325N, S52.325P, S52.325Q, S52.325R, S52.326K, S52.326M, S52.326N, S52.326P, S52.326Q, S52.326R, S52.331K, S52.331M, S52.331N, S52.331P, S52.331Q, S52.331R, S52.332K, S52.332M, S52.332N, S52.332P, S52.332Q, S52.332R, S52.333K, S52.333M, S52.333N, S52.333P, S52.333Q, S52.333R, S52.334K, S52.334M, S52.334N, S52.334P, S52.334Q, S52.334R, S52.335K, S52.335M, S52.335N, S52.335P, S52.335Q, S52.335R, S52.336K, S52.336M, S52.336N, S52.336P, S52.336Q, S52.336R, S52.341K, S52.341M, S52.341N, S52.341P, S52.341Q, S52.341R, S52.342K, S52.342M, S52.342N, S52.342P,

© 2025 Park EE et al. *JAMA Network Open*.

		S52.342Q, S52.342R, S52.343K, S52.343M, S52.343N, S52.343P, S52.343Q, S52.343R, S52.344K, S52.344M, S52.344N, S52.344P, S52.344Q, S52.344R, S52.345K, S52.345M, S52.345N, S52.345P, S52.345Q, S52.345R, S52.346K, S52.346M, S52.346N, S52.346P, S52.346Q, S52.346R, S52.351K, S52.351M, S52.351N, S52.351P, S52.351Q, S52.351R, S52.352K, S52.352M, S52.352N, S52.352P, S52.352Q, S52.352R, S52.353K, S52.353M, S52.353N, S52.353P, S52.353Q, S52.353R, S52.354K, S52.354M, S52.354N, S52.354P, S52.354Q, S52.354R, S52.355K, S52.355M, S52.355N, S52.355P, S52.355Q, S52.355R, S52.356K, S52.356M, S52.356N, S52.356P, S52.356Q, S52.356R, S52.361K, S52.361M, S52.361N, S52.361P, S52.361Q, S52.361R, S52.362K, S52.362M, S52.362N, S52.362P, S52.362Q, S52.362R, S52.363K, S52.363M, S52.363N, S52.363P, S52.363Q, S52.363R, S52.364K, S52.364M, S52.364N, S52.364P, S52.364Q, S52.364R, S52.365K, S52.365M, S52.365N, S52.365P, S52.365Q, S52.365R, S52.366K, S52.366M, S52.366N, S52.366P, S52.366Q, S52.366R, S52.371K, S52.371M, S52.371N, S52.371P, S52.371Q, S52.371R, S52.372K, S52.372M, S52.372N, S52.372P, S52.372Q, S52.372R, S52.379K, S52.379M, S52.379N, S52.379P, S52.379Q, S52.379R, S52.381K, S52.381M, S52.381N, S52.381P, S52.381Q, S52.381R, S52.382K, S52.382M, S52.382N, S52.382P, S52.382Q, S52.382R, S52.389K, S52.389M, S52.389N, S52.389P, S52.389Q, S52.389R, S52.391K, S52.391M, S52.391N, S52.391P, S52.391Q, S52.391R, S52.392K, S52.392M, S52.392N, S52.392P, S52.392Q, S52.392R, S52.399K, S52.399M, S52.399N, S52.399P, S52.399Q, S52.399R, S52.501K, S52.501M, S52.501N, S52.501P, S52.501Q, S52.501R, S52.502K, S52.502M, S52.502N, S52.502P, S52.502Q, S52.502R, S52.509K, S52.509M, S52.509N, S52.509P, S52.509Q, S52.509R, S52.511K, S52.511M, S52.511N, S52.511P, S52.511Q, S52.511R, S52.512K, S52.512M, S52.512N, S52.512P, S52.512Q, S52.512R, S52.513K, S52.513M, S52.513N, S52.513P, S52.513Q, S52.513R, S52.514K, S52.514M, S52.514N, S52.514P, S52.514Q, S52.514R, S52.515K, S52.515M, S52.515N, S52.515P, S52.515Q, S52.515R, S52.516K, S52.516M, S52.516N, S52.516P, S52.516Q, S52.516R, S52.521K, S52.521P, S52.522K, S52.522P, S52.529K, S52.529P, S52.531K, S52.531M, S52.531N, S52.531P, S52.531Q, S52.531R, S52.532K, S52.532M, S52.532N, S52.532P, S52.532Q, S52.532R, S52.539K, S52.539M, S52.539N, S52.539P, S52.539Q, S52.539R, S52.541K, S52.541M, S52.541N, S52.541P, S52.541Q, S52.541R, S52.542K, S52.542M, S52.542N, S52.542P, S52.542Q, S52.542R, S52.549K, S52.549M, S52.549N, S52.549P, S52.549Q, S52.549R, S52.551K, S52.551M, S52.551N, S52.551P, S52.551Q, S52.551R, S52.552K, S52.552M, S52.552N, S52.552P, S52.552Q, S52.552R, S52.559K, S52.559M, S52.559N, S52.559P, S52.559Q, S52.559R, S52.561K, S52.561M, S52.561N, S52.561P, S52.561Q, S52.561R, S52.562K, S52.562M, S52.562N, S52.562P, S52.562Q, S52.562R, S52.569K, S52.569M, S52.569N, S52.569P, S52.569Q, S52.569R, S52.571K, S52.571M, S52.571N, S52.571P, S52.571Q, S52.571R, S52.572K, S52.572M, S52.572N, S52.572P, S52.572Q, S52.572R, S52.579K, S52.579M, S52.579N, S52.579P, S52.579Q, S52.579R, S52.591K, S52.591M, S52.591N, S52.591P, S52.591Q, S52.591R, S52.592K, S52.592M, S52.592N, S52.592P, S52.592Q, S52.592R, S52.599K, S52.599M, S52.599N, S52.599P, S52.599Q, S52.599R, S52.601K, S52.601M, S52.601N, S52.601P, S52.601Q, S52.601R, S52.602K, S52.602M, S52.602N, S52.602P, S52.602Q, S52.602R, S52.609K, S52.609M, S52.609N, S52.609P, S52.609Q, S52.609R, S52.611K, S52.611M, S52.611N, S52.611P, S52.611Q, S52.611R, S52.612K, S52.612M, S52.612N, S52.612P, S52.612Q, S52.612R, S52.613K, S52.613M, S52.613N, S52.613P, S52.613Q, S52.613R, S52.614K, S52.614M, S52.614N, S52.614P, S52.614Q, S52.614R, S52.615K, S52.615M, S52.615N, S52.615P, S52.615Q, S52.615R, S52.616K, S52.616M, S52.616N, S52.616P, S52.616Q, S52.616R, S52.621K, S52.621P, S52.622K, S52.622P, S52.629K, S52.629P, S52.691K, S52.691M, S52.691N, S52.691P, S52.691Q, S52.691R, S52.692K, S52.692M, S52.692N, S52.692P, S52.692Q, S52.692R, S52.699K, S52.699M, S52.699N, S52.699P, S52.699Q, S52.699R, S52.90XK, S52.90XM, S52.90XN, S52.90XP, S52.90XQ, S52.90XR, S52.91XK, S52.91XM, S52.91XN, S52.91XP, S52.91XQ, S52.91XR, S52.92XK, S52.92XM, S52.92XN, S52.92XP, S52.92XQ, S52.92XR, S59.001K, S59.001P, S59.002K, S59.002P,

© 2025 Park EE et al. *JAMA Network Open*.

		S59.009K, S59.009P, S59.011K, S59.011P, S59.012K, S59.012P, S59.019K, S59.019P, S59.021K, S59.021P, S59.022K, S59.022P, S59.029K, S59.029P, S59.031K, S59.031P, S59.032K, S59.032P, S59.039K, S59.039P, S59.041K, S59.041P, S59.042K, S59.042P, S59.049K, S59.049P, S59.091K, S59.091P, S59.092K, S59.092P, S59.099K, S59.099P, S59.101K, S59.101P, S59.102K, S59.102P, S59.109K, S59.109P, S59.111K, S59.111P, S59.112K, S59.112P, S59.119K, S59.119P, S59.121K, S59.121P, S59.122K, S59.122P, S59.129K, S59.129P, S59.131K, S59.131P, S59.132K, S59.132P, S59.139K, S59.139P, S59.141K, S59.141P, S59.142K, S59.142P, S59.149K, S59.149P, S59.191K, S59.191P, S59.192K, S59.192P, S59.199K, S59.199P, S59.201K, S59.201P, S59.202K, S59.202P, S59.209K, S59.209P, S59.211K, S59.211P, S59.212K, S59.212P, S59.219K, S59.219P, S59.221K, S59.221P, S59.222K, S59.222P, S59.229K, S59.229P, S59.231K, S59.231P, S59.232K, S59.232P, S59.239K, S59.239P, S59.241K, S59.241P, S59.242K, S59.242P, S59.249K, S59.249P, S59.291K, S59.291P, S59.292K, S59.292P, S59.299K, S59.299P, S62.001K, S62.001P, S62.002K, S62.002P, S62.009K, S62.009P, S62.011K, S62.011P, S62.012K, S62.012P, S62.013K, S62.013P, S62.014K, S62.014P, S62.015K, S62.015P, S62.016K, S62.016P, S62.021K, S62.021P, S62.022K, S62.022P, S62.023K, S62.023P, S62.024K, S62.024P, S62.025K, S62.025P, S62.026K, S62.026P, S62.031K, S62.031P, S62.032K, S62.032P, S62.033K, S62.033P, S62.034K, S62.034P, S62.035K, S62.035P, S62.036K, S62.036P, S62.101K, S62.101P, S62.102K, S62.102P, S62.109K, S62.109P, S62.111K, S62.111P, S62.112K, S62.112P, S62.113K, S62.113P, S62.114K, S62.114P, S62.115K, S62.115P, S62.116K, S62.116P, S62.121K, S62.121P, S62.122K, S62.122P, S62.123K, S62.123P, S62.124K, S62.124P, S62.125K, S62.125P, S62.126K, S62.126P, S62.131K, S62.131P, S62.132K, S62.132P, S62.133K, S62.133P, S62.134K, S62.134P, S62.135K, S62.135P, S62.136K, S62.136P, S62.141K, S62.141P, S62.142K, S62.142P, S62.143K, S62.143P, S62.144K, S62.144P, S62.145K, S62.145P, S62.146K, S62.146P, S62.151K, S62.151P, S62.152K, S62.152P, S62.153K, S62.153P, S62.154K, S62.154P, S62.155K, S62.155P, S62.156K, S62.156P, S62.161K, S62.161P, S62.162K, S62.162P, S62.163K, S62.163P, S62.164K, S62.164P, S62.165K, S62.165P, S62.166K, S62.166P, S62.171K, S62.171P, S62.172K, S62.172P, S62.173K, S62.173P, S62.174K, S62.174P, S62.175K, S62.175P, S62.176K, S62.176P, S62.181K, S62.181P, S62.182K, S62.182P, S62.183K, S62.183P, S62.184K, S62.184P, S62.185K, S62.185P, S62.186K, S62.186P, S62.201K, S62.201P, S62.202K, S62.202P, S62.209K, S62.209P, S62.211K, S62.211P, S62.212K, S62.212P, S62.213K, S62.213P, S62.221K, S62.221P, S62.222K, S62.222P, S62.223K, S62.223P, S62.224K, S62.224P, S62.225K, S62.225P, S62.226K, S62.226P, S62.231K, S62.231P, S62.232K, S62.232P, S62.233K, S62.233P, S62.234K, S62.234P, S62.235K, S62.235P, S62.236K, S62.236P, S62.241K, S62.241P, S62.242K, S62.242P, S62.243K, S62.243P, S62.244K, S62.244P, S62.245K, S62.245P, S62.246K, S62.246P, S62.251K, S62.251P, S62.252K, S62.252P, S62.253K, S62.253P, S62.254K, S62.254P, S62.255K, S62.255P, S62.256K, S62.256P, S62.291K, S62.291P, S62.292K, S62.292P, S62.299K, S62.299P, S62.300K, S62.300P, S62.301K, S62.301P, S62.302K, S62.302P, S62.303K, S62.303P, S62.304K, S62.304P, S62.305K, S62.305P, S62.306K, S62.306P, S62.307K, S62.307P, S62.308K, S62.308P, S62.309K, S62.309P, S62.310K, S62.310P, S62.311K, S62.311P, S62.312K, S62.312P, S62.313K, S62.313P, S62.314K, S62.314P, S62.315K, S62.315P, S62.316K, S62.316P, S62.317K, S62.317P, S62.318K, S62.318P, S62.319K, S62.319P, S62.320K, S62.320P, S62.321K, S62.321P, S62.322K, S62.322P, S62.323K, S62.323P, S62.324K, S62.324P, S62.325K, S62.325P, S62.326K, S62.326P, S62.327K, S62.327P, S62.328K, S62.328P, S62.329K, S62.329P, S62.330K, S62.330P, S62.331K, S62.331P, S62.332K, S62.332P, S62.333K, S62.333P, S62.334K, S62.334P, S62.335K, S62.335P, S62.336K, S62.336P, S62.337K, S62.337P, S62.338K, S62.338P, S62.339K, S62.339P, S62.340K, S62.340P, S62.341K, S62.341P, S62.342K, S62.342P, S62.343K, S62.343P, S62.344K, S62.344P, S62.345K, S62.345P, S62.346K, S62.346P, S62.347K, S62.347P, S62.348K, S62.348P, S62.349K, S62.349P, S62.350K, S62.350P, S62.351K, S62.351P, S62.352K, S62.352P,

© 2025 Park EE et al. *JAMA Network Open*.

		S62.353K, S62.353P, S62.354K, S62.354P, S62.355K, S62.355P, S62.356K, S62.356P, S62.357K, S62.357P, S62.358K, S62.358P, S62.359K, S62.359P, S62.360K, S62.360P, S62.361K, S62.361P, S62.362K, S62.362P, S62.363K, S62.363P, S62.364K, S62.364P, S62.365K, S62.365P, S62.366K, S62.366P, S62.367K, S62.367P, S62.368K, S62.368P, S62.369K, S62.369P, S62.390K, S62.390P, S62.391K, S62.391P, S62.392K, S62.392P, S62.393K, S62.393P, S62.394K, S62.394P, S62.395K, S62.395P, S62.396K, S62.396P, S62.397K, S62.397P, S62.398K, S62.398P, S62.399K, S62.399P, S62.501K, S62.501P, S62.502K, S62.502P, S62.509K, S62.509P, S62.511K, S62.511P, S62.512K, S62.512P, S62.513K, S62.513P, S62.514K, S62.514P, S62.515K, S62.515P, S62.516K, S62.516P, S62.521K, S62.521P, S62.522K, S62.522P, S62.523K, S62.523P, S62.524K, S62.524P, S62.525K, S62.525P, S62.526K, S62.526P, S62.600K, S62.600P, S62.601K, S62.601P, S62.602K, S62.602P, S62.603K, S62.603P, S62.604K, S62.604P, S62.605K, S62.605P, S62.606K, S62.606P, S62.607K, S62.607P, S62.608K, S62.608P, S62.609K, S62.609P, S62.610K, S62.610P, S62.611K, S62.611P, S62.612K, S62.612P, S62.613K, S62.613P, S62.614K, S62.614P, S62.615K, S62.615P, S62.616K, S62.616P, S62.617K, S62.617P, S62.618K, S62.618P, S62.619K, S62.619P, S62.620K, S62.620P, S62.621K, S62.621P, S62.622K, S62.622P, S62.623K, S62.623P, S62.624K, S62.624P, S62.625K, S62.625P, S62.626K, S62.626P, S62.627K, S62.627P, S62.628K, S62.628P, S62.629K, S62.629P, S62.630K, S62.630P, S62.631K, S62.631P, S62.632K, S62.632P, S62.633K, S62.633P, S62.634K, S62.634P, S62.635K, S62.635P, S62.636K, S62.636P, S62.637K, S62.637P, S62.638K, S62.638P, S62.639K, S62.639P, S62.640K, S62.640P, S62.641K, S62.641P, S62.642K, S62.642P, S62.643K, S62.643P, S62.644K, S62.644P, S62.645K, S62.645P, S62.646K, S62.646P, S62.647K, S62.647P, S62.648K, S62.648P, S62.649K, S62.649P, S62.650K, S62.650P, S62.651K, S62.651P, S62.652K, S62.652P, S62.653K, S62.653P, S62.654K, S62.654P, S62.655K, S62.655P, S62.656K, S62.656P, S62.657K, S62.657P, S62.658K, S62.658P, S62.659K, S62.659P, S62.660K, S62.660P, S62.661K, S62.661P, S62.662K, S62.662P, S62.663K, S62.663P, S62.664K, S62.664P, S62.665K, S62.665P, S62.666K, S62.666P, S62.667K, S62.667P, S62.668K, S62.668P, S62.669K, S62.669P, S62.90XK, S62.90XP, S62.91XK, S62.91XP, S62.92XK, S62.92XP, S72.001K, S72.001M, S72.001N, S72.001P, S72.001Q, S72.001R, S72.002K, S72.002M, S72.002N, S72.002P, S72.002Q, S72.002R, S72.009K, S72.009M, S72.009N, S72.009P, S72.009Q, S72.009R, S72.011K, S72.011M, S72.011N, S72.011P, S72.011Q, S72.011R, S72.012K, S72.012M, S72.012N, S72.012P, S72.012Q, S72.012R, S72.019K, S72.019M, S72.019N, S72.019P, S72.019Q, S72.019R, S72.021K, S72.021M, S72.021N, S72.021P, S72.021Q, S72.021R, S72.022K, S72.022M, S72.022N, S72.022P, S72.022Q, S72.022R, S72.023K, S72.023M, S72.023N, S72.023P, S72.023Q, S72.023R, S72.024K, S72.024M, S72.024N, S72.024P, S72.024Q, S72.024R, S72.025K, S72.025M, S72.025N, S72.025P, S72.025Q, S72.025R, S72.026K, S72.026M, S72.026N, S72.026P, S72.026Q, S72.026R, S72.031K, S72.031M, S72.031N, S72.031P, S72.031Q, S72.031R, S72.032K, S72.032M, S72.032N, S72.032P, S72.032Q, S72.032R, S72.033K, S72.033M, S72.033N, S72.033P, S72.033Q, S72.033R, S72.034K, S72.034M, S72.034N, S72.034P, S72.034Q, S72.034R, S72.035K, S72.035M, S72.035N, S72.035P, S72.035Q, S72.035R, S72.036K, S72.036M, S72.036N, S72.036P, S72.036Q, S72.036R, S72.041K, S72.041M, S72.041N, S72.041P, S72.041Q, S72.041R, S72.042K, S72.042M, S72.042N, S72.042P, S72.042Q, S72.042R, S72.043K, S72.043M, S72.043N, S72.043P, S72.043Q, S72.043R, S72.044K, S72.044M, S72.044N, S72.044P, S72.044Q, S72.044R, S72.045K, S72.045M, S72.045N, S72.045P, S72.045Q, S72.045R, S72.046K, S72.046M, S72.046N, S72.046P, S72.046Q, S72.046R, S72.051K, S72.051M, S72.051N, S72.051P, S72.051Q, S72.051R, S72.052K, S72.052M, S72.052N, S72.052P, S72.052Q, S72.052R, S72.059K, S72.059M, S72.059N, S72.059P, S72.059Q, S72.059R, S72.061K, S72.061M, S72.061N, S72.061P, S72.061Q, S72.061R, S72.062K, S72.062M, S72.062N, S72.062P, S72.062Q, S72.062R, S72.063K, S72.063M, S72.063N, S72.063P, S72.063Q, S72.063R, S72.064K, S72.064M, S72.064N, S72.064P, S72.064Q, S72.064R, S72.065K, S72.065M, S72.065N, S72.065P,

© 2025 Park EE et al. *JAMA Network Open*.

		S72.065Q, S72.065R, S72.066K, S72.066M, S72.066N, S72.066P, S72.066Q, S72.066R, S72.091K, S72.091M, S72.091N, S72.091P, S72.091Q, S72.091R, S72.092K, S72.092M, S72.092N, S72.092P, S72.092Q, S72.092R, S72.099K, S72.099M, S72.099N, S72.099P, S72.099Q, S72.099R, S72.101K, S72.101M, S72.101N, S72.101P, S72.101Q, S72.101R, S72.102K, S72.102M, S72.102N, S72.102P, S72.102Q, S72.102R, S72.109K, S72.109M, S72.109N, S72.109P, S72.109Q, S72.109R, S72.111K, S72.111M, S72.111N, S72.111P, S72.111Q, S72.111R, S72.112K, S72.112M, S72.112N, S72.112P, S72.112Q, S72.112R, S72.113K, S72.113M, S72.113N, S72.113P, S72.113Q, S72.113R, S72.114K, S72.114M, S72.114N, S72.114P, S72.114Q, S72.114R, S72.115K, S72.115M, S72.115N, S72.115P, S72.115Q, S72.115R, S72.116K, S72.116M, S72.116N, S72.116P, S72.116Q, S72.116R, S72.121K, S72.121M, S72.121N, S72.121P, S72.121Q, S72.121R, S72.122K, S72.122M, S72.122N, S72.122P, S72.122Q, S72.122R, S72.123K, S72.123M, S72.123N, S72.123P, S72.123Q, S72.123R, S72.124K, S72.124M, S72.124N, S72.124P, S72.124Q, S72.124R, S72.125K, S72.125M, S72.125N, S72.125P, S72.125Q, S72.125R, S72.126K, S72.126M, S72.126N, S72.126P, S72.126Q, S72.126R, S72.131K, S72.131M, S72.131N, S72.131P, S72.131Q, S72.131R, S72.132K, S72.132M, S72.132N, S72.132P, S72.132Q, S72.132R, S72.133K, S72.133M, S72.133N, S72.133P, S72.133Q, S72.133R, S72.134K, S72.134M, S72.134N, S72.134P, S72.134Q, S72.134R, S72.135K, S72.135M, S72.135N, S72.135P, S72.135Q, S72.135R, S72.136K, S72.136M, S72.136N, S72.136P, S72.136Q, S72.136R, S72.141K, S72.141M, S72.141N, S72.141P, S72.141Q, S72.141R, S72.142K, S72.142M, S72.142N, S72.142P, S72.142Q, S72.142R, S72.143K, S72.143M, S72.143N, S72.143P, S72.143Q, S72.143R, S72.144K, S72.144M, S72.144N, S72.144P, S72.144Q, S72.144R, S72.145K, S72.145M, S72.145N, S72.145P, S72.145Q, S72.145R, S72.146K, S72.146M, S72.146N, S72.146P, S72.146Q, S72.146R, S72.21XK, S72.21XM, S72.21XN, S72.21XP, S72.21XQ, S72.21XR, S72.22XK, S72.22XM, S72.22XN, S72.22XP, S72.22XQ, S72.22XR, S72.23XK, S72.23XM, S72.23XN, S72.23XP, S72.23XQ, S72.23XR, S72.24XK, S72.24XM, S72.24XN, S72.24XP, S72.24XQ, S72.24XR, S72.25XK, S72.25XM, S72.25XN, S72.25XP, S72.25XQ, S72.25XR, S72.26XK, S72.26XM, S72.26XN, S72.26XP, S72.26XQ, S72.26XR, S72.301K, S72.301M, S72.301N, S72.301P, S72.301Q, S72.301R, S72.302K, S72.302M, S72.302N, S72.302P, S72.302Q, S72.302R, S72.309K, S72.309M, S72.309N, S72.309P, S72.309Q, S72.309R, S72.321K, S72.321M, S72.321N, S72.321P, S72.321Q, S72.321R, S72.322K, S72.322M, S72.322N, S72.322P, S72.322Q, S72.322R, S72.323K, S72.323M, S72.323N, S72.323P, S72.323Q, S72.323R, S72.324K, S72.324M, S72.324N, S72.324P, S72.324Q, S72.324R, S72.325K, S72.325M, S72.325N, S72.325P, S72.325Q, S72.325R, S72.326K, S72.326M, S72.326N, S72.326P, S72.326Q, S72.326R, S72.331K, S72.331M, S72.331N, S72.331P, S72.331Q, S72.331R, S72.332K, S72.332M, S72.332N, S72.332P, S72.332Q, S72.332R, S72.333K, S72.333M, S72.333N, S72.333P, S72.333Q, S72.333R, S72.334K, S72.334M, S72.334N, S72.334P, S72.334Q, S72.334R, S72.335K, S72.335M, S72.335N, S72.335P, S72.335Q, S72.335R, S72.336K, S72.336M, S72.336N, S72.336P, S72.336Q, S72.336R, S72.341K, S72.341M, S72.341N, S72.341P, S72.341Q, S72.341R, S72.342K, S72.342M, S72.342N, S72.342P, S72.342Q, S72.342R, S72.343K, S72.343M, S72.343N, S72.343P, S72.343Q, S72.343R, S72.344K, S72.344M, S72.344N, S72.344P, S72.344Q, S72.344R, S72.345K, S72.345M, S72.345N, S72.345P, S72.345Q, S72.345R, S72.346K, S72.346M, S72.346N, S72.346P, S72.346Q, S72.346R, S72.351K, S72.351M, S72.351N, S72.351P, S72.351Q, S72.351R, S72.352K, S72.352M, S72.352N, S72.352P, S72.352Q, S72.352R, S72.353K, S72.353M, S72.353N, S72.353P, S72.353Q, S72.353R, S72.354K, S72.354M, S72.354N, S72.354P, S72.354Q, S72.354R, S72.355K, S72.355M, S72.355N, S72.355P, S72.355Q, S72.355R, S72.356K, S72.356M, S72.356N, S72.356P, S72.356Q, S72.356R, S72.361K, S72.361M, S72.361N, S72.361P, S72.361Q, S72.361R, S72.362K, S72.362M, S72.362N, S72.362P, S72.362Q, S72.362R, S72.363K, S72.363M, S72.363N, S72.363P, S72.363Q, S72.363R, S72.364K, S72.364M, S72.364N, S72.364P, S72.364Q, S72.364R, S72.365K, S72.365M, S72.365N, S72.365P, S72.365Q, S72.365R, S72.366K, S72.366M, S72.366N, S72.366P,

© 2025 Park EE et al. *JAMA Network Open*.

		S72.366Q, S72.366R, S72.391K, S72.391M, S72.391N, S72.391P, S72.391Q, S72.391R, S72.392K, S72.392M, S72.392N, S72.392P, S72.392Q, S72.392R, S72.399K, S72.399M, S72.399N, S72.399P, S72.399Q, S72.399R, S72.401K, S72.401M, S72.401N, S72.401P, S72.401Q, S72.401R, S72.402K, S72.402M, S72.402N, S72.402P, S72.402Q, S72.402R, S72.409K, S72.409M, S72.409N, S72.409P, S72.409Q, S72.409R, S72.411K, S72.411M, S72.411N, S72.411P, S72.411Q, S72.411R, S72.412K, S72.412M, S72.412N, S72.412P, S72.412Q, S72.412R, S72.413K, S72.413M, S72.413N, S72.413P, S72.413Q, S72.413R, S72.414K, S72.414M, S72.414N, S72.414P, S72.414Q, S72.414R, S72.415K, S72.415M, S72.415N, S72.415P, S72.415Q, S72.415R, S72.416K, S72.416M, S72.416N, S72.416P, S72.416Q, S72.416R, S72.421K, S72.421M, S72.421N, S72.421P, S72.421Q, S72.421R, S72.422K, S72.422M, S72.422N, S72.422P, S72.422Q, S72.422R, S72.423K, S72.423M, S72.423N, S72.423P, S72.423Q, S72.423R, S72.424K, S72.424M, S72.424N, S72.424P, S72.424Q, S72.424R, S72.425K, S72.425M, S72.425N, S72.425P, S72.425Q, S72.425R, S72.426K, S72.426M, S72.426N, S72.426P, S72.426Q, S72.426R, S72.431K, S72.431M, S72.431N, S72.431P, S72.431Q, S72.431R, S72.432K, S72.432M, S72.432N, S72.432P, S72.432Q, S72.432R, S72.433K, S72.433M, S72.433N, S72.433P, S72.433Q, S72.433R, S72.434K, S72.434M, S72.434N, S72.434P, S72.434Q, S72.434R, S72.435K, S72.435M, S72.435N, S72.435P, S72.435Q, S72.435R, S72.436K, S72.436M, S72.436N, S72.436P, S72.436Q, S72.436R, S72.441K, S72.441M, S72.441N, S72.441P, S72.441Q, S72.441R, S72.442K, S72.442M, S72.442N, S72.442P, S72.442Q, S72.442R, S72.443K, S72.443M, S72.443N, S72.443P, S72.443Q, S72.443R, S72.444K, S72.444M, S72.444N, S72.444P, S72.444Q, S72.444R, S72.445K, S72.445M, S72.445N, S72.445P, S72.445Q, S72.445R, S72.446K, S72.446M, S72.446N, S72.446P, S72.446Q, S72.446R, S72.451K, S72.451M, S72.451N, S72.451P, S72.451Q, S72.451R, S72.452K, S72.452M, S72.452N, S72.452P, S72.452Q, S72.452R, S72.453K, S72.453M, S72.453N, S72.453P, S72.453Q, S72.453R, S72.454K, S72.454M, S72.454N, S72.454P, S72.454Q, S72.454R, S72.455K, S72.455M, S72.455N, S72.455P, S72.455Q, S72.455R, S72.456K, S72.456M, S72.456N, S72.456P, S72.456Q, S72.456R, S72.461K, S72.461M, S72.461N, S72.461P, S72.461Q, S72.461R, S72.462K, S72.462M, S72.462N, S72.462P, S72.462Q, S72.462R, S72.463K, S72.463M, S72.463N, S72.463P, S72.463Q, S72.463R, S72.464K, S72.464M, S72.464N, S72.464P, S72.464Q, S72.464R, S72.465K, S72.465M, S72.465N, S72.465P, S72.465Q, S72.465R, S72.466K, S72.466M, S72.466N, S72.466P, S72.466Q, S72.466R, S72.471K, S72.471P, S72.472K, S72.472P, S72.479K, S72.479P, S72.491K, S72.491M, S72.491N, S72.491P, S72.491Q, S72.491R, S72.492K, S72.492M, S72.492N, S72.492P, S72.492Q, S72.492R, S72.499K, S72.499M, S72.499N, S72.499P, S72.499Q, S72.499R, S72.8X1K, S72.8X1M, S72.8X1N, S72.8X1P, S72.8X1Q, S72.8X1R, S72.8X2K, S72.8X2M, S72.8X2N, S72.8X2P, S72.8X2Q, S72.8X2R, S72.8X9K, S72.8X9M, S72.8X9N, S72.8X9P, S72.8X9Q, S72.8X9R, S72.90XK, S72.90XM, S72.90XN, S72.90XP, S72.90XQ, S72.90XR, S72.91XK, S72.91XM, S72.91XN, S72.91XP, S72.91XQ, S72.91XR, S72.92XK, S72.92XM, S72.92XN, S72.92XP, S72.92XQ, S72.92XR, S79.001K, S79.001P, S79.002K, S79.002P, S79.009K, S79.009P, S79.011K, S79.011P, S79.012K, S79.012P, S79.019K, S79.019P, S79.091K, S79.091P, S79.092K, S79.092P, S79.099K, S79.099P, S79.101K, S79.101P, S79.102K, S79.102P, S79.109K, S79.109P, S79.111K, S79.111P, S79.112K, S79.112P, S79.119K, S79.119P, S79.121K, S79.121P, S79.122K, S79.122P, S79.129K, S79.129P, S79.131K, S79.131P, S79.132K, S79.132P, S79.139K, S79.139P, S79.141K, S79.141P, S79.142K, S79.142P, S79.149K, S79.149P, S79.191K, S79.191P, S79.192K, S79.192P, S79.199K, S79.199P, S82.001K, S82.001M, S82.001N, S82.001P, S82.001Q, S82.001R, S82.002K, S82.002M, S82.002N, S82.002P, S82.002Q, S82.002R, S82.009K, S82.009M, S82.009N, S82.009P, S82.009Q, S82.009R, S82.011K, S82.011M, S82.011N, S82.011P, S82.011Q, S82.011R, S82.012K, S82.012M, S82.012N, S82.012P, S82.012Q, S82.012R, S82.013K, S82.013M, S82.013N, S82.013P, S82.013Q, S82.013R, S82.014K, S82.014M, S82.014N, S82.014P, S82.014Q, S82.014R, S82.015K, S82.015M, S82.015N, S82.015P, S82.015Q, S82.015R, S82.016K, S82.016M, S82.016N, S82.016P,

© 2025 Park EE et al. *JAMA Network Open*.

		S82.016Q, S82.016R, S82.021K, S82.021M, S82.021N, S82.021P, S82.021Q, S82.021R, S82.022K, S82.022M, S82.022N, S82.022P, S82.022Q, S82.022R, S82.023K, S82.023M, S82.023N, S82.023P, S82.023Q, S82.023R, S82.024K, S82.024M, S82.024N, S82.024P, S82.024Q, S82.024R, S82.025K, S82.025M, S82.025N, S82.025P, S82.025Q, S82.025R, S82.026K, S82.026M, S82.026N, S82.026P, S82.026Q, S82.026R, S82.031K, S82.031M, S82.031N, S82.031P, S82.031Q, S82.031R, S82.032K, S82.032M, S82.032N, S82.032P, S82.032Q, S82.032R, S82.033K, S82.033M, S82.033N, S82.033P, S82.033Q, S82.033R, S82.034K, S82.034M, S82.034N, S82.034P, S82.034Q, S82.034R, S82.035K, S82.035M, S82.035N, S82.035P, S82.035Q, S82.035R, S82.036K, S82.036M, S82.036N, S82.036P, S82.036Q, S82.036R, S82.041K, S82.041M, S82.041N, S82.041P, S82.041Q, S82.041R, S82.042K, S82.042M, S82.042N, S82.042P, S82.042Q, S82.042R, S82.043K, S82.043M, S82.043N, S82.043P, S82.043Q, S82.043R, S82.044K, S82.044M, S82.044N, S82.044P, S82.044Q, S82.044R, S82.045K, S82.045M, S82.045N, S82.045P, S82.045Q, S82.045R, S82.046K, S82.046M, S82.046N, S82.046P, S82.046Q, S82.046R, S82.091K, S82.091M, S82.091N, S82.091P, S82.091Q, S82.091R, S82.092K, S82.092M, S82.092N, S82.092P, S82.092Q, S82.092R, S82.099K, S82.099M, S82.099N, S82.099P, S82.099Q, S82.099R, S82.101K, S82.101M, S82.101N, S82.101P, S82.101Q, S82.101R, S82.102K, S82.102M, S82.102N, S82.102P, S82.102Q, S82.102R, S82.109K, S82.109M, S82.109N, S82.109P, S82.109Q, S82.109R, S82.111K, S82.111M, S82.111N, S82.111P, S82.111Q, S82.111R, S82.112K, S82.112M, S82.112N, S82.112P, S82.112Q, S82.112R, S82.113K, S82.113M, S82.113N, S82.113P, S82.113Q, S82.113R, S82.114K, S82.114M, S82.114N, S82.114P, S82.114Q, S82.114R, S82.115K, S82.115M, S82.115N, S82.115P, S82.115Q, S82.115R, S82.116K, S82.116M, S82.116N, S82.116P, S82.116Q, S82.116R, S82.121K, S82.121M, S82.121N, S82.121P, S82.121Q, S82.121R, S82.122K, S82.122M, S82.122N, S82.122P, S82.122Q, S82.122R, S82.123K, S82.123M, S82.123N, S82.123P, S82.123Q, S82.123R, S82.124K, S82.124M, S82.124N, S82.124P, S82.124Q, S82.124R, S82.125K, S82.125M, S82.125N, S82.125P, S82.125Q, S82.125R, S82.126K, S82.126M, S82.126N, S82.126P, S82.126Q, S82.126R, S82.131K, S82.131M, S82.131N, S82.131P, S82.131Q, S82.131R, S82.132K, S82.132M, S82.132N, S82.132P, S82.132Q, S82.132R, S82.133K, S82.133M, S82.133N, S82.133P, S82.133Q, S82.133R, S82.134K, S82.134M, S82.134N, S82.134P, S82.134Q, S82.134R, S82.135K, S82.135M, S82.135N, S82.135P, S82.135Q, S82.135R, S82.136K, S82.136M, S82.136N, S82.136P, S82.136Q, S82.136R, S82.141K, S82.141M, S82.141N, S82.141P, S82.141Q, S82.141R, S82.142K, S82.142M, S82.142N, S82.142P, S82.142Q, S82.142R, S82.143K, S82.143M, S82.143N, S82.143P, S82.143Q, S82.143R, S82.144K, S82.144M, S82.144N, S82.144P, S82.144Q, S82.144R, S82.145K, S82.145M, S82.145N, S82.145P, S82.145Q, S82.145R, S82.146K, S82.146M, S82.146N, S82.146P, S82.146Q, S82.146R, S82.151K, S82.151M, S82.151N, S82.151P, S82.151Q, S82.151R, S82.152K, S82.152M, S82.152N, S82.152P, S82.152Q, S82.152R, S82.153K, S82.153M, S82.153N, S82.153P, S82.153Q, S82.153R, S82.154K, S82.154M, S82.154N, S82.154P, S82.154Q, S82.154R, S82.155K, S82.155M, S82.155N, S82.155P, S82.155Q, S82.155R, S82.156K, S82.156M, S82.156N, S82.156P, S82.156Q, S82.156R, S82.161K, S82.161P, S82.162K, S82.162P, S82.169K, S82.169P, S82.191K, S82.191M, S82.191N, S82.191P, S82.191Q, S82.191R, S82.192K, S82.192M, S82.192N, S82.192P, S82.192Q, S82.192R, S82.199K, S82.199M, S82.199N, S82.199P, S82.199Q, S82.199R, S82.201K, S82.201M, S82.201N, S82.201P, S82.201Q, S82.201R, S82.202K, S82.202M, S82.202N, S82.202P, S82.202Q, S82.202R, S82.209K, S82.209M, S82.209N, S82.209P, S82.209Q, S82.209R, S82.221K, S82.221M, S82.221N, S82.221P, S82.221Q, S82.221R, S82.222K, S82.222M, S82.222N, S82.222P, S82.222Q, S82.222R, S82.223K, S82.223M, S82.223N, S82.223P, S82.223Q, S82.223R, S82.224K, S82.224M, S82.224N, S82.224P, S82.224Q, S82.224R, S82.225K, S82.225M, S82.225N, S82.225P, S82.225Q, S82.225R, S82.226K, S82.226M, S82.226N, S82.226P, S82.226Q, S82.226R, S82.231K, S82.231M, S82.231N, S82.231P, S82.231Q, S82.231R, S82.232K, S82.232M, S82.232N, S82.232P, S82.232Q, S82.232R, S82.233K, S82.233M, S82.233N, S82.233P,

© 2025 Park EE et al. *JAMA Network Open*.

		S82.233Q, S82.233R, S82.234K, S82.234M, S82.234N, S82.234P, S82.234Q, S82.234R, S82.235K, S82.235M, S82.235N, S82.235P, S82.235Q, S82.235R, S82.236K, S82.236M, S82.236N, S82.236P, S82.236Q, S82.236R, S82.241K, S82.241M, S82.241N, S82.241P, S82.241Q, S82.241R, S82.242K, S82.242M, S82.242N, S82.242P, S82.242Q, S82.242R, S82.243K, S82.243M, S82.243N, S82.243P, S82.243Q, S82.243R, S82.244K, S82.244M, S82.244N, S82.244P, S82.244Q, S82.244R, S82.245K, S82.245M, S82.245N, S82.245P, S82.245Q, S82.245R, S82.246K, S82.246M, S82.246N, S82.246P, S82.246Q, S82.246R, S82.251K, S82.251M, S82.251N, S82.251P, S82.251Q, S82.251R, S82.252K, S82.252M, S82.252N, S82.252P, S82.252Q, S82.252R, S82.253K, S82.253M, S82.253N, S82.253P, S82.253Q, S82.253R, S82.254K, S82.254M, S82.254N, S82.254P, S82.254Q, S82.254R, S82.255K, S82.255M, S82.255N, S82.255P, S82.255Q, S82.255R, S82.256K, S82.256M, S82.256N, S82.256P, S82.256Q, S82.256R, S82.261K, S82.261M, S82.261N, S82.261P, S82.261Q, S82.261R, S82.262K, S82.262M, S82.262N, S82.262P, S82.262Q, S82.262R, S82.263K, S82.263M, S82.263N, S82.263P, S82.263Q, S82.263R, S82.264K, S82.264M, S82.264N, S82.264P, S82.264Q, S82.264R, S82.265K, S82.265M, S82.265N, S82.265P, S82.265Q, S82.265R, S82.266K, S82.266M, S82.266N, S82.266P, S82.266Q, S82.266R, S82.291K, S82.291M, S82.291N, S82.291P, S82.291Q, S82.291R, S82.292K, S82.292M, S82.292N, S82.292P, S82.292Q, S82.292R, S82.299K, S82.299M, S82.299N, S82.299P, S82.299Q, S82.299R, S82.301K, S82.301M, S82.301N, S82.301P, S82.301Q, S82.301R, S82.302K, S82.302M, S82.302N, S82.302P, S82.302Q, S82.302R, S82.309K, S82.309M, S82.309N, S82.309P, S82.309Q, S82.309R, S82.311K, S82.311P, S82.312K, S82.312P, S82.319K, S82.319P, S82.391K, S82.391M, S82.391N, S82.391P, S82.391Q, S82.391R, S82.392K, S82.392M, S82.392N, S82.392P, S82.392Q, S82.392R, S82.399K, S82.399M, S82.399N, S82.399P, S82.399Q, S82.399R, S82.401K, S82.401M, S82.401N, S82.401P, S82.401Q, S82.401R, S82.402K, S82.402M, S82.402N, S82.402P, S82.402Q, S82.402R, S82.409K, S82.409M, S82.409N, S82.409P, S82.409Q, S82.409R, S82.421K, S82.421M, S82.421N, S82.421P, S82.421Q, S82.421R, S82.422K, S82.422M, S82.422N, S82.422P, S82.422Q, S82.422R, S82.423K, S82.423M, S82.423N, S82.423P, S82.423Q, S82.423R, S82.424K, S82.424M, S82.424N, S82.424P, S82.424Q, S82.424R, S82.425K, S82.425M, S82.425N, S82.425P, S82.425Q, S82.425R, S82.426K, S82.426M, S82.426N, S82.426P, S82.426Q, S82.426R, S82.431K, S82.431M, S82.431N, S82.431P, S82.431Q, S82.431R, S82.432K, S82.432M, S82.432N, S82.432P, S82.432Q, S82.432R, S82.433K, S82.433M, S82.433N, S82.433P, S82.433Q, S82.433R, S82.434K, S82.434M, S82.434N, S82.434P, S82.434Q, S82.434R, S82.435K, S82.435M, S82.435N, S82.435P, S82.435Q, S82.435R, S82.436K, S82.436M, S82.436N, S82.436P, S82.436Q, S82.436R, S82.441K, S82.441M, S82.441N, S82.441P, S82.441Q, S82.441R, S82.442K, S82.442M, S82.442N, S82.442P, S82.442Q, S82.442R, S82.443K, S82.443M, S82.443N, S82.443P, S82.443Q, S82.443R, S82.444K, S82.444M, S82.444N, S82.444P, S82.444Q, S82.444R, S82.445K, S82.445M, S82.445N, S82.445P, S82.445Q, S82.445R, S82.446K, S82.446M, S82.446N, S82.446P, S82.446Q, S82.446R, S82.451K, S82.451M, S82.451N, S82.451P, S82.451Q, S82.451R, S82.452K, S82.452M, S82.452N, S82.452P, S82.452Q, S82.452R, S82.453K, S82.453M, S82.453N, S82.453P, S82.453Q, S82.453R, S82.454K, S82.454M, S82.454N, S82.454P, S82.454Q, S82.454R, S82.455K, S82.455M, S82.455N, S82.455P, S82.455Q, S82.455R, S82.456K, S82.456M, S82.456N, S82.456P, S82.456Q, S82.456R, S82.461K, S82.461M, S82.461N, S82.461P, S82.461Q, S82.461R, S82.462K, S82.462M, S82.462N, S82.462P, S82.462Q, S82.462R, S82.463K, S82.463M, S82.463N, S82.463P, S82.463Q, S82.463R, S82.464K, S82.464M, S82.464N, S82.464P, S82.464Q, S82.464R, S82.465K, S82.465M, S82.465N, S82.465P, S82.465Q, S82.465R, S82.466K, S82.466M, S82.466N, S82.466P, S82.466Q, S82.466R, S82.491K, S82.491M, S82.491N, S82.491P, S82.491Q, S82.491R, S82.492K, S82.492M, S82.492N, S82.492P, S82.492Q, S82.492R, S82.499K, S82.499M, S82.499N, S82.499P, S82.499Q, S82.499R, S82.51XK, S82.51XM, S82.51XN, S82.51XP, S82.51XQ, S82.51XR, S82.52XK, S82.52XM, S82.52XN, S82.52XP, S82.52XQ, S82.52XR, S82.53XK, S82.53XM, S82.53XN, S82.53XP,

© 2025 Park EE et al. *JAMA Network Open*.

		S82.53XQ, S82.53XR, S82.54XK, S82.54XM, S82.54XN, S82.54XP, S82.54XQ, S82.54XR, S82.55XK, S82.55XM, S82.55XN, S82.55XP, S82.55XQ, S82.55XR, S82.56XK, S82.56XM, S82.56XN, S82.56XP, S82.56XQ, S82.56XR, S82.61XK, S82.61XM, S82.61XN, S82.61XP, S82.61XQ, S82.61XR, S82.62XK, S82.62XM, S82.62XN, S82.62XP, S82.62XQ, S82.62XR, S82.63XK, S82.63XM, S82.63XN, S82.63XP, S82.63XQ, S82.63XR, S82.64XK, S82.64XM, S82.64XN, S82.64XP, S82.64XQ, S82.64XR, S82.65XK, S82.65XM, S82.65XN, S82.65XP, S82.65XQ, S82.65XR, S82.66XK, S82.66XM, S82.66XN, S82.66XP, S82.66XQ, S82.66XR, S82.811K, S82.811P, S82.812K, S82.812P, S82.819K, S82.819P, S82.821K, S82.821P, S82.822K, S82.822P, S82.829K, S82.829P, S82.831K, S82.831M, S82.831N, S82.831P, S82.831Q, S82.831R, S82.832K, S82.832M, S82.832N, S82.832P, S82.832Q, S82.832R, S82.839K, S82.839M, S82.839N, S82.839P, S82.839Q, S82.839R, S82.841K, S82.841M, S82.841N, S82.841P, S82.841Q, S82.841R, S82.842K, S82.842M, S82.842N, S82.842P, S82.842Q, S82.842R, S82.843K, S82.843M, S82.843N, S82.843P, S82.843Q, S82.843R, S82.844K, S82.844M, S82.844N, S82.844P, S82.844Q, S82.844R, S82.845K, S82.845M, S82.845N, S82.845P, S82.845Q, S82.845R, S82.846K, S82.846M, S82.846N, S82.846P, S82.846Q, S82.846R, S82.851K, S82.851M, S82.851N, S82.851P, S82.851Q, S82.851R, S82.852K, S82.852M, S82.852N, S82.852P, S82.852Q, S82.852R, S82.853K, S82.853M, S82.853N, S82.853P, S82.853Q, S82.853R, S82.854K, S82.854M, S82.854N, S82.854P, S82.854Q, S82.854R, S82.855K, S82.855M, S82.855N, S82.855P, S82.855Q, S82.855R, S82.856K, S82.856M, S82.856N, S82.856P, S82.856Q, S82.856R, S82.861K, S82.861M, S82.861N, S82.861P, S82.861Q, S82.861R, S82.862K, S82.862M, S82.862N, S82.862P, S82.862Q, S82.862R, S82.863K, S82.863M, S82.863N, S82.863P, S82.863Q, S82.863R, S82.864K, S82.864M, S82.864N, S82.864P, S82.864Q, S82.864R, S82.865K, S82.865M, S82.865N, S82.865P, S82.865Q, S82.865R, S82.866K, S82.866M, S82.866N, S82.866P, S82.866Q, S82.866R, S82.871K, S82.871M, S82.871N, S82.871P, S82.871Q, S82.871R, S82.872K, S82.872M, S82.872N, S82.872P, S82.872Q, S82.872R, S82.873K, S82.873M, S82.873N, S82.873P, S82.873Q, S82.873R, S82.874K, S82.874M, S82.874N, S82.874P, S82.874Q, S82.874R, S82.875K, S82.875M, S82.875N, S82.875P, S82.875Q, S82.875R, S82.876K, S82.876M, S82.876N, S82.876P, S82.876Q, S82.876R, S82.891K, S82.891M, S82.891N, S82.891P, S82.891Q, S82.891R, S82.892K, S82.892M, S82.892N, S82.892P, S82.892Q, S82.892R, S82.899K, S82.899M, S82.899N, S82.899P, S82.899Q, S82.899R, S82.90XK, S82.90XM, S82.90XN, S82.90XP, S82.90XQ, S82.90XR, S82.91XK, S82.91XM, S82.91XN, S82.91XP, S82.91XQ, S82.91XR, S82.92XK, S82.92XM, S82.92XN, S82.92XP, S82.92XQ, S82.92XR, S89.001K, S89.001P, S89.002K, S89.002P, S89.009K, S89.009P, S89.011K, S89.011P, S89.012K, S89.012P, S89.019K, S89.019P, S89.021K, S89.021P, S89.022K, S89.022P, S89.029K, S89.029P, S89.031K, S89.031P, S89.032K, S89.032P, S89.039K, S89.039P, S89.041K, S89.041P, S89.042K, S89.042P, S89.049K, S89.049P, S89.091K, S89.091P, S89.092K, S89.092P, S89.099K, S89.099P, S89.101K, S89.101P, S89.102K, S89.102P, S89.109K, S89.109P, S89.111K, S89.111P, S89.112K, S89.112P, S89.119K, S89.119P, S89.121K, S89.121P, S89.122K, S89.122P, S89.129K, S89.129P, S89.131K, S89.131P, S89.132K, S89.132P, S89.139K, S89.139P, S89.141K, S89.141P, S89.142K, S89.142P, S89.149K, S89.149P, S89.191K, S89.191P, S89.192K, S89.192P, S89.199K, S89.199P, S89.201K, S89.201P, S89.202K, S89.202P, S89.209K, S89.209P, S89.211K, S89.211P, S89.212K, S89.212P, S89.219K, S89.219P, S89.221K, S89.221P, S89.222K, S89.222P, S89.229K, S89.229P, S89.291K, S89.291P, S89.292K, S89.292P, S89.299K, S89.299P, S89.301K, S89.301P, S89.302K, S89.302P, S89.309K, S89.309P, S89.311K, S89.311P, S89.312K, S89.312P, S89.319K, S89.319P, S89.321K, S89.321P, S89.322K, S89.322P, S89.329K, S89.329P, S89.391K, S89.391P, S89.392K, S89.392P, S89.399K, S89.399P, S92.001K, S92.001P, S92.002K, S92.002P, S92.009K, S92.009P, S92.011K, S92.011P, S92.012K, S92.012P, S92.013K, S92.013P, S92.014K, S92.014P, S92.015K, S92.015P, S92.016K, S92.016P, S92.021K, S92.021P, S92.022K, S92.022P, S92.023K, S92.023P, S92.024K, S92.024P, S92.025K, S92.025P, S92.026K, S92.026P, S92.031K, S92.031P, S92.032K, S92.032P,

© 2025 Park EE et al. *JAMA Network Open*.

		S92.033K, S92.033P, S92.034K, S92.034P, S92.035K, S92.035P, S92.036K, S92.036P, S92.041K, S92.041P, S92.042K, S92.042P, S92.043K, S92.043P, S92.044K, S92.044P, S92.045K, S92.045P, S92.046K, S92.046P, S92.051K, S92.051P, S92.052K, S92.052P, S92.053K, S92.053P, S92.054K, S92.054P, S92.055K, S92.055P, S92.056K, S92.056P, S92.061K, S92.061P, S92.062K, S92.062P, S92.063K, S92.063P, S92.064K, S92.064P, S92.065K, S92.065P, S92.066K, S92.066P, S92.101K, S92.101P, S92.102K, S92.102P, S92.109K, S92.109P, S92.111K, S92.111P, S92.112K, S92.112P, S92.113K, S92.113P, S92.114K, S92.114P, S92.115K, S92.115P, S92.116K, S92.116P, S92.121K, S92.121P, S92.122K, S92.122P, S92.123K, S92.123P, S92.124K, S92.124P, S92.125K, S92.125P, S92.126K, S92.126P, S92.131K, S92.131P, S92.132K, S92.132P, S92.133K, S92.133P, S92.134K, S92.134P, S92.135K, S92.135P, S92.136K, S92.136P, S92.141K, S92.141P, S92.142K, S92.142P, S92.143K, S92.143P, S92.144K, S92.144P, S92.145K, S92.145P, S92.146K, S92.146P, S92.151K, S92.151P, S92.152K, S92.152P, S92.153K, S92.153P, S92.154K, S92.154P, S92.155K, S92.155P, S92.156K, S92.156P, S92.191K, S92.191P, S92.192K, S92.192P, S92.199K, S92.199P, S92.201K, S92.201P, S92.202K, S92.202P, S92.209K, S92.209P, S92.211K, S92.211P, S92.212K, S92.212P, S92.213K, S92.213P, S92.214K, S92.214P, S92.215K, S92.215P, S92.216K, S92.216P, S92.221K, S92.221P, S92.222K, S92.222P, S92.223K, S92.223P, S92.224K, S92.224P, S92.225K, S92.225P, S92.226K, S92.226P, S92.231K, S92.231P, S92.232K, S92.232P, S92.233K, S92.233P, S92.234K, S92.234P, S92.235K, S92.235P, S92.236K, S92.236P, S92.241K, S92.241P, S92.242K, S92.242P, S92.243K, S92.243P, S92.244K, S92.244P, S92.245K, S92.245P, S92.246K, S92.246P, S92.251K, S92.251P, S92.252K, S92.252P, S92.253K, S92.253P, S92.254K, S92.254P, S92.255K, S92.255P, S92.256K, S92.256P, S92.301K, S92.301P, S92.302K, S92.302P, S92.309K, S92.309P, S92.311K, S92.311P, S92.312K, S92.312P, S92.313K, S92.313P, S92.314K, S92.314P, S92.315K, S92.315P, S92.316K, S92.316P, S92.321K, S92.321P, S92.322K, S92.322P, S92.323K, S92.323P, S92.324K, S92.324P, S92.325K, S92.325P, S92.326K, S92.326P, S92.331K, S92.331P, S92.332K, S92.332P, S92.333K, S92.333P, S92.334K, S92.334P, S92.335K, S92.335P, S92.336K, S92.336P, S92.341K, S92.341P, S92.342K, S92.342P, S92.343K, S92.343P, S92.344K, S92.344P, S92.345K, S92.345P, S92.346K, S92.346P, S92.351K, S92.351P, S92.352K, S92.352P, S92.353K, S92.353P, S92.354K, S92.354P, S92.355K, S92.355P, S92.356K, S92.356P, S92.401K, S92.401P, S92.402K, S92.402P, S92.403K, S92.403P, S92.404K, S92.404P, S92.405K, S92.405P, S92.406K, S92.406P, S92.411K, S92.411P, S92.412K, S92.412P, S92.413K, S92.413P, S92.414K, S92.414P, S92.415K, S92.415P, S92.416K, S92.416P, S92.421K, S92.421P, S92.422K, S92.422P, S92.423K, S92.423P, S92.424K, S92.424P, S92.425K, S92.425P, S92.426K, S92.426P, S92.491K, S92.491P, S92.492K, S92.492P, S92.499K, S92.499P, S92.501K, S92.501P, S92.502K, S92.502P, S92.503K, S92.503P, S92.504K, S92.504P, S92.505K, S92.505P, S92.506K, S92.506P, S92.511K, S92.511P, S92.512K, S92.512P, S92.513K, S92.513P, S92.514K, S92.514P, S92.515K, S92.515P, S92.516K, S92.516P, S92.521K, S92.521P, S92.522K, S92.522P, S92.523K, S92.523P, S92.524K, S92.524P, S92.525K, S92.525P, S92.526K, S92.526P, S92.531K, S92.531P, S92.532K, S92.532P, S92.533K, S92.533P, S92.534K, S92.534P, S92.535K, S92.535P, S92.536K, S92.536P, S92.591K, S92.591P, S92.592K, S92.592P, S92.599K, S92.599P, S92.811K, S92.811P, S92.812K, S92.812P, S92.819K, S92.819P, S92.901K, S92.901P, S92.902K, S92.902P, S92.909K, S92.909P, S92.911K, S92.911P, S92.912K, S92.912P, S92.919K, S92.919P, S99.001K, S99.001P, S99.002K, S99.002P, S99.009K, S99.009P, S99.011K, S99.011P, S99.012K, S99.012P, S99.019K, S99.019P, S99.021K, S99.021P, S99.022K, S99.022P, S99.029K, S99.029P, S99.031K, S99.031P, S99.032K, S99.032P, S99.039K, S99.039P, S99.041K, S99.041P, S99.042K, S99.042P, S99.049K, S99.049P, S99.091K, S99.091P, S99.092K, S99.092P, S99.099K, S99.099P, S99.101K, S99.101P, S99.102K, S99.102P, S99.109K, S99.109P, S99.111K, S99.111P, S99.112K, S99.112P, S99.119K, S99.119P, S99.121K, S99.121P, S99.122K, S99.122P, S99.129K, S99.129P, S99.131K, S99.131P, S99.132K, S99.132P,

© 2025 Park EE et al. *JAMA Network Open*.

		S99.139K, S99.139P, S99.141K, S99.141P, S99.142K, S99.142P, S99.149K, S99.149P, S99.191K, S99.191P, S99.192K, S99.192P, S99.199K, S99.199P, S99.201K, S99.201P, S99.202K, S99.202P, S99.209K, S99.209P, S99.211K, S99.211P, S99.212K, S99.212P, S99.219K, S99.219P, S99.221K, S99.221P, S99.222K, S99.222P, S99.229K, S99.229P, S99.231K, S99.231P, S99.232K, S99.232P, S99.239K, S99.239P, S99.241K, S99.241P, S99.242K, S99.242P, S99.249K, S99.249P, S99.291K, S99.291P, S99.292K, S99.292P, S99.299K, S99.299P
**Headache, including migraine**	307.81, 346.00, 346.01, 346.02, 346.03, 346.10, 346.11, 346.12, 346.13, 346.20, 346.21, 346.22, 346.23, 346.30, 346.31, 346.32, 346.33, 346.40, 346.41, 346.42, 346.43, 346.50, 346.51, 346.52, 346.53, 346.60, 346.61, 346.62, 346.63, 346.70, 346.71, 346.72, 346.73, 346.80, 346.81, 346.82, 346.83, 346.90, 346.91, 346.92, 346.93, 784.0	G43.001, G43.009, G43.011, G43.019, G43.101, G43.109, G43.111, G43.119, G43.401, G43.409, G43.411, G43.419, G43.501, G43.509, G43.511, G43.519, G43.601, G43.609, G43.611, G43.619, G43.701, G43.709, G43.711, G43.719, G43.801, G43.809, G43.811, G43.819, G43.821, G43.829, G43.831, G43.839, G43.901, G43.909, G43.911, G43.919, G43.B0, G43.B1, G43.C0, G43.C1, G44.1, G44.209, R51
**Chronic Pain due to other causes[1,2]**	568.0, 568.81, 568.82, 568.89, 569.0, 569.1, 569.2, 569.3, 569.41, 569.42, 569.43, 569.44, 569.49, 569.5, 569.60, 569.61, 569.62, 569.69, 569.71, 569.79, 569.81, 569.82, 569.83, 569.84, 569.85, 569.86, 569.87, 569.89, 574.10, 574.11, 574.20, 574.21, 574.40, 574.41, 574.50, 574.51, 574.70, 574.71, 574.80, 574.81, 574.90, 574.91, 575.10, 575.11, 575.12, 575.2, 575.3, 575.5, 575.6, 575.8, 575.9, 576.0, 576.2, 576.3, 576.4, 576.5, 576.8, 577.1, 577.2, 577.8, 577.9, 592.0,	K50.013, K50.014, K50.113, K50.114, K50.813, K50.814, K50.913, K50.914, K51.013, K51.014, K51.213, K51.214, K51.313, K51.314, K51.413, K51.414, K51.513, K51.514, K51.813, K51.814, K51.913, K51.914, K55.20, K55.21, K57.00, K57.01, K57.20, K57.21, K57.40, K57.41, K57.80, K57.81, K62.0, K62.1, K62.2, K62.3, K62.4, K62.5, K62.6, K62.7, K62.81, K62.82, K62.89, K62.9, K63.0, K63.1, K63.2, K63.3, K63.4, K63.81, K63.89, K66.0, K66.1, K66.8, K68.9, K80.10, K80.11, K80.12, K80.13, K80.18, K80.19, K80.20, K80.21, K80.30, K80.31, K80.32, K80.33, K80.34, K80.35, K80.36, K80.37, K80.40, K80.41, K80.44, K80.45, K80.46, K80.47, K80.50, K80.51, K80.60, K80.61, K80.64, K80.65, K80.66, K80.67, K80.70, K80.71, K80.80, K80.81, K81.1, K81.2, K81.9, K82.0, K82.1, K82.3, K82.4, K82.8, K82.9, K83.1, K83.2, K83.3, K83.4, K83.5, K83.8, K86.0, K86.1, K86.2, K86.3, K86.81, K86.89, K86.9, K87, K91.5, K91.850, K91.858, K92.89, K94.00, K94.01, K94.02, K94.03, K94.09, K94.10, K94.11, K94.12, K94.13, K94.19, M99.09, N11.1, N13.2, N13.8, N13.9, N20.0, N20.1, N20.2, N20.9, N22, N99.4, R10.10, R10.11, R10.12, R10.13, R10.2, R10.30, R10.31, R10.32, R10.33, R10.84, R10.9, R11.13, R18.8, R19.00, R19.01, R19.02, R19.03, R19.04, R19.05, R19.06, R19.07, R19.09

© 2025 Park EE et al. *JAMA Network Open*.

	592.1, 592.9, 593.4, 739.9, 789.00, 789.01, 789.02, 789.03, 789.04, 789.05, 789.06, 789.07, 789.09, 789.30, 789.31, 789.32, 789.33, 789.34, 789.35, 789.36, 789.37, 789.39	
**Chronic Pain, unspecified cause**[1,2]	338.21, 338.22, 338.28, 338.29, 338.4	G89.21, G89.22, G89.28, G89.29, G89.4
**Chronic Pain due to procedures**	CPT Codes: 20526, 20527, 20550, 20551, 20552, 20553, 20555, 20600, 20605, 20610, 20612, 21116, 23350, 24220, 25246, 26035, 27093, 27095, 27096, 27370, 27648	

eTable 4. Heart Failure (HF) Outcome *ICD-9*/*ICD-10* Definitions

ICD-9*	***ICD-10*****
Primary position<br><br>398.91^<br>402.01<br>402.11<br>402.91<br>404.01<br>404.03^<br>404.11<br>404.13^<br>404.91<br>404.93^<br>425.x^<br>428.x	Primary position<br><br>I09.81^<br>I09.9<br>I11.0<br>I13.0<br>I13.2<br>I25.5<br>I42.0<br>I42.1-I42.4^<br>I42.5-I42.9<br>I43.x<br>I50.x<br>I97.130^<br>I97.131^<br>P29.0

*ICD-9 codes have a reported PPV of 99%[1]
**ICD-10 codes have been shown to have a sensitivity of 0.80, a specificity of 0.98, and a PPV of 93.6%[2]
^Codes used for HF history only.

© 2025 Park EE et al. *JAMA Network Open*.

eTable 5. Extended Table of Variables Used to Develop Propensity Score (Unweighted)

Variable	Pregabalin % Yes	Gabapentin % Yes	SD	Overall % Yes
**Demographics**				
Male sex	6156 (33.06)	75836(33.32)	-.006	81992 (33.30)
Black (Or African-American)	1311 (7.04)	16395(7.2)	-.006	17706 (7.19)
Non-Hispanic White	13985(75.10)	182731(80.28)	-.125	
Other race[a] (American Indian/Alaska Native, Asian/Pacific Islander, Hispanic, other, and unknown)	3326 (17.86)	28490(12.52)	.149	31816 (12.92)
Rural zip code	4077 (21.89)	55136(24.22)	-.055	59213 (24.05)
**Chronic Pain Indications**				
Hospitalization	1025 (5.50)	10759 (4.73)	.035	11784 (4.79)
Arthralgia	9570 (51.39)	109602 (48.15)	.065	119172 (48.40)
Unspecified cause	3865 (20.76)	39676 (17.43)	.085	43541 (17.68)
Autoimmune/other rheumatic diseases	811(4.36)	7940 (3.49)	.045	8751 (3.55)
Back pain/degenerative back disorders	12734 (68.38)	154920 (68.06)	.007	167654 (68.09)
Fibromyalgia	3817(20.50)	30801 (13.53)	.186	34618 (14.06)
Headache, including migraine	2817 (15.13)	32624 (14.33)	.022	35441 (14.39)
Inflammatory	4558(24.48)	49637 (21.81)	.063	54195 (22.01)
Neuropathic	11744 (63.07)	138031 (60.64)	.050	149775 (60.82)
Other musculoskeletal/soft tissue pain	13793 (74.07)	162423 (71.36)	.061	176216 (71.56)
Other causes	5567(29.86)	62822 (27.63)	.049	68443 (27.80)
Procedures	5698(30.60)	60980 (26.79)	.084	66678 (27.08)
**Pain Medications**				
Short-Acting Opioid Analgesics Use	10910(58.59)	117197 (51.49)	.143	128107 (52.03)
Analgesics, other	449(2.41)	4806 (2.11)	.020	5255 (2.13)
Coxibs	1597 (8.58)	11303 (4.97)	.144	12900 (5.24)
Cyclobenzaprine	1187 (6.37)	132027 (5.72)	.027	14214 (5.77)
DMARDs	1628 (8.74)	15263 (6.71)	.076	16891 (6.86)
Other skeletal muscle relaxants	2253(12.10)	22634 (9.94)	.069	24887 (10.11)
NSAIDs, non-selective	7061 (37.92)	82160 (36.10)	.038	89221 (36.23)
Systemic oral corticosteroids	5647(30.32)	70049 (30.77)	-.010	75696 (30.74)

© 2025 Park EE et al. *JAMA Network Open*.

Migraines, triptans	339 (1.82)	3524 (1.55)	.021	3863 (1.57)
Anticonvulsants, Primary Use Mood Stabilizer	271(1.46)	2751 (1.21)	.022	3022 (1.23)
Anticonvulsants, Occasional Use Mood Stabilizer, Pain	403 (2.16)	3485 (1.53)	.047	3888 (1.58)
Bupropion	737 (3.96)	8127 (3.57)	.020	8864 (3.60)
Duloxetine	1887(10.13)	11853 (5.21)	.186	13740 (5.58)
Mirtazapine	421 (2.26)	4236 (1.86)	.028	4657 (1.89)
SNRI, Other	675 (3.62)	6892 (3.03)	.033	7567 (3.07)
SSRIs	3764(20.21)	44963 (19.75)	.011	48727 (19.79)
TCA/Heterocyclic	1243 (6.67)	10913 (4.79)	.081	12156 (4.94)
Benzodiazepines	6487 (34.84)	69654 (30.60)	.090	76141 (30.92)
Trazadone	1363 (7.32)	14670 (6.45)	.035	16033 (6.51)
Sedatives other than benzos	2335 (12.54)	21741(9.55)	.095	24076 (9.78)
**Cardiovascular Diagnoses**				
Ill-defined or unspecified descriptions or complications of heart disease	1013(5.44)	11930(5.24)	.009	12943 (5.26)
Cerebrovascular disease, late effects	208(1.12)	2367 (1.04)	.007	2575 (1.05)
Cardiovascular Hospitalization	367(1.97)	4451 (1.96)	.001	4818 (1.96)
Other chronic ischemic heart disease	4138(22.22)	46454 (20.41)	.044	50592 (20.55)
Arrhythmia: atrial fibrillation/flutter	1622(8.71)	19742 (8.67)	.001	21364 (8.68)
Myocardial infarction	620 (3.33)	7576 (3.33)	0	8196 (3.33)
Angina	836 (4.49)	8773 (3.85)	.032	9609 (3.90)
Arrhythmia, except atrial fibrillation	857 (4.60)	8644 (3.80)	.040	9501 (3.86)
Arrythmia, non-threatening (ICD10 only)	1390 (7.46)	17805 (7.82)	-.013	19195 (7.80)
Arterial embolism and thrombosis	704 (3.78)	7050 (3.10)	.037	7754 (3.15)
CABG	606 (3.25)	7466 (3.28)	-.001	8072 (3.28)
Cardiac symptoms	2175 (11.68)	23757 (10.44)	.040	25932 (10.53)
Conduction disorder	749 (4.02)	9329 (4.10)	-.004	10078 (4.09)
Diabetes (complications other than neuropathy)	2754 (14.79)	26867 (11.80)	.088	29621 (12.03)
Diabetes (any, other than with complications)	7462 (40.07)	77479 (34.04)	.125	84941 (34.50)
Venous disorders: phlebitis, thrombosis, varicosity with complications, lymphatics	1811 (9.73)	19203 (8.44)	.045	21014 (8.53)
Hypertension, benign or unspecified	14963 (80.35)	179656 (78.93)	.035	194619 (79.04)
Hypertension, malignant	256 (1.37)	2624 (1.15)	.020	2880 (1.17)

© 2025 Park EE et al. *JAMA Network Open*.

Hyperlipidemia	13886 (74.57)	167889 (73.76)	.018	181775 (73.82)
Hypotension	520 (2.79)	5716 (2.51)	.018	6236 (2.53)
Ischemic or unspecified stroke	2030 (10.90)	22013 (9.67)	.041	24043 (9.76)
Obesity, morbid	1678 (9.01)	18813 (8.27)	.027	20491 (8.32)
Obesity, not morbid or unspecified	3445 (18.50)	41403 (18.19)	.008	44848 (18.21)
Other cerebrovascular disease	548 (2.94)	6587 (2.89)	.003	7135 (2.90)
Pacemaker	567 (3.04)	6451 (2.83)	.012	7018 (2.85)
Peripheral vascular disease	3824 (20.53)	39419 (17.32)	.082	43243 (17.56)
Percutaneous intervention	832 (4.47)	9910 (4.35)	.006	10742 (4.36)
Smoking and smoking-related disorders	3483 (18.70)	43240 (19.00)	-.007	46723 (18.97)
Syncope	818 (4.39)	9514 (4.18)	.011	10332 (4.20)
TIA	520 (2.79)	5725 (2.52)	.017	6245 (2.54)
Valve disease other than mitral valve	1528 (8.21)	17510 (7.69)	.019	19038 (7.73)
Valve disease, mitral valve (including stenosis)	1372 (7.37)	14611 (6.42)	.037	15983 (6.49)
**Other Diagnoses and Procedures**				
Asphyxia or hypoxemia	306 (1.64)	3419 (1.50)	.011	3725 (1.51)
Asthma	1998 (10.73)	22549 (9.91)	.027	24547 (9.97)
Chronic obstructive pulmonary disease	2573 (13.82)	28056 (12.33)	.044	30629 (12.44)
Continuous positive airway pressure	1305 (7.01)	16363 (7.19)	-.007	17668 (7.18)
Home oxygen	975 (5.24)	11673 (5.13)	.005	12648 (5.14)
Other respiratory diseases	1202 (6.45)	14395 (6.32)	.005	15597 (6.33)
Pneumonia	629 (3.38)	7395 (3.25)	.007	8024 (3.26)
Sleep apnea	2159 (11.59)	26150 (11.49)	.003	28309 (11.50)
Other respiratory symptoms	6617 (35.53)	76834 (33.76)	.037	83451 (33.89)
Other cognitive impairment	840 (4.51)	9959 (4.38)	.007	10799 (4.39)
Alzheimer and other dementias	884 (4.75)	9101 (4.00)	.037	9985 (4.05)
Other neurologic disorders	697 (3.74)	6247 (2.74)	.056	6944 (2.82)
Parkinsons and other movement disorders	1496 (8.03)	19548 (8.59)	-.020	21044 (8.55)
Other peripheral nervous system disorders	4205 (22.58)	43159 (18.96)	.089	47364 (19.23)

© 2025 Park EE et al. *JAMA Network Open*.

Seizure disorders and convulsions	323 (1.73)	3559 (1.56)	.013	3882 (1.58)
Gastrointestinal bleeding, unspecified	442 (2.37)	5078 (2.23)	.010	5520 (2.24)
Gastrointestinal bleeding, lower	352 (1.89)	4355 (1.91)	-.002	4707 (1.91)
Gallbladder/bile duct	526 (2.82)	6320 (2.78)	.003	6846 (2.78)
Constipation, irritable bowel syndrome	2811 (15.10)	29217 (12.84)	.065	32028 (13.01)
Crohn disease/ulcerative colitis	203 (1.09)	2311 (1.02)	.007	2514 (1.02)
Liver, hepatitis	229 (1.23)	2241 (0.98)	.023	2470 (1.00)
Liver, other	1120 (6.01)	12064 (5.30)	.031	13184 (5.35)
Gastroparesis, intestinal obstruction	252 (1.35)	2543 (1.12)	.021	2795 (1.14)
Diverticulitis/diverticulosis without bleeding	1988 (10.68)	25185 (11.06)	-.012	27173 (11.04)
Esophagitis/gastritis/duodenitis without bleeding	2260 (12.14)	22087 (9.70)	.078	24347 (9.89)
Hemorrhoids without bleeding	1230 (6.61)	14309 (6.29)	.013	15539 (6.31)
Peptic ulcer disease without bleeding	381 (2.05)	3426 (1.51)	.410	3807 (1.55)
G.I. symptoms, abdominal pain	4409 (23.68)	48339 (21.24)	.058	52748 (21.42)
G.I. symptoms, dyspepsia	947 (5.09)	9115 (4.00)	.052	10062 (4.09)
G.I. symptoms, other lower	1734 (9.31)	19594 (8.61)	.025	21328 (8.66)
G.I. symptoms, other upper	2636 (14.16)	29588 (13.00)	.034	32224 (13.09)
G.I. symptoms, gastroesophageal reflux	5651 (30.35)	67706 (29.75)	.013	73357 (29.79)
Debility, not specified	1565 (8.40)	16886 (7.42)	.037	18451 (7.49)
Diabetic footwear	746 (4.01)	5612 (2.47)	.087	6358 (2.58)
Dysphagia	785 (4.22)	9273 (4.07)	.007	10058 (4.08)
Malnutrition/abnormal weight loss/feeding problems	919 (4.94)	10167 (4.47)	.022	11086 (4.50)
Limited mobility, cane or walker	755 (4.05)	7872 (3.46)	.031	8627 (3.50)
Limited mobility, wheelchair	253 (1.36)	2249 (0.99)	.034	2502 (1.02)
Osteoporosis	3260 (17.51)	35058 (15.40)	.057	38318 (15.56)
Chronic skin ulcer	636 (3.42)	6024 (2.65)	.045	6660 (2.70)
Unintentional fall (not vigorous activity)	1765 (9.48)	21541 (9.46)	0	23306 (9.46)
Urinary incontinence	1435 (7.71)	15482 (6.80)	.035	16917 (6.87)
Anemia, iron-deficiency	997 (5.35)	11275 (4.95)	.018	12272 (4.98)

© 2025 Park EE et al. *JAMA Network Open*.

Genitourinary bleeding	1197 (6.43)	14340 (6.30)	.005	15537 (6.31)
Bleeding, unspecified site	556 (2.99)	6062 (2.66)	.019	6618 (2.69)
Coagulation defects and other hemorrhagic conditions	646 (3.47)	7042 (3.09)	.021	7688 (3.12)
Bleeding, other sites	375 (2.01)	4234 (1.86)	.011	4609 (1.87)
Other disorders blood, blood forming organs	842 (4.52)	9451 (4.15)	.018	10293 (4.18)
Acute renal failure	337 (1.81)	3916 (1.72)	.007	4253 (1.73)
Chronic renal failure, Stage I-IV/Unspecified	2169 (11.65)	26376 (11.59)	.002	28545 (11.59)
Other renal disease	310 (1.66)	3578 (1.57)	.007	3888 (1.58)
Other kidney disorders	2349 (12.61)	27595 (12.12)	.015	29944 (12.16)
Other serious metabolic disorders	1105 (5.93)	12566 (5.52)	.018	13671 (5.55)
Adverse medication effect (not opioids or psychotropic drugs)	529 (2.84)	5649 (2.48)	.022	6178 (2.51)
History of cancer other than non-melanoma skin cancer	1091 (5.86)	14076 (6.18)	-.014	15167 (6.16)
Skin cancer, not melanoma	1002 (5.38)	13629 (5.99)	-.026	14631 (5.94)
Infections, EENT	7410 (39.79)	87003 (38.22)	.032	94413 (38.34)
Other serious endocrine disorders	496 (2.66)	5522 (2.43)	.015	6018 (2.44)
Hypovolemia	625 (3.36)	5890 (2.59)	.045	6515 (2.65)
Disorders potassium/sodium balance	1351 (7.25)	15205 (6.68)	.023	16556 (6.72)
Other fluid/electrolyte disorders	216 (1.16)	2353 (1.03)	.012	2569 (1.04)
Other hospitalization	252 (1.35)	2329 (1.02)	.030	2581 (1.05)
Adverse effect of medical device, graft, or procedure	945 (5.07)	9452 (4.15)	.044	10397 (4.22)
Other infectious and parasitic diseases, serious	1926 (10.34)	21799 (9.58)	.026	23725 (9.63)
Infections, minor	5055 (27.15)	56206 (24.69)	.056	61261 (24.88)
Urinary tract infections	3791 (20.36)	40902 (17.97)	.061	44693 (18.15)
Neoplasms, not malignant or carcinoma in situ	5392 (28.95)	70350 (30.91)	-.043	75742 (30.76)
Disorders of prostate	2593 (13.92)	29789 (13.09)	.024	32382 (13.15)
Swelling, mass, or lump: head, neck, chest, abdomen	501 (2.69)	5866 (2.58)	.007	6367 (2.59)
Goiter/other thyroid disorders	1536 (8.25)	17502 (7.69)	.021	19038 (7.73)
Hyperthyroidism	300 (1.61)	3520 (1.55)	.005	3820 (1.55)
Hypothyroidism	5680 (30.50)	64308 (28.25)	.049	69988 (28.42)

© 2025 Park EE et al. *JAMA Network Open*.

Prophylactic vaccination	12196 (65.49)	154168 (67.73)	-.047	166364 (67.56)
Major/moderate trauma, head	260 (1.40)	3275 (1.44)	-.004	3535 (1.44)
Hip replacement	649 (3.49)	7743 (3.40)	.005	8392 (3.41)
Other joint replacement	215 (1.15)	2395 (1.05)	.010	2610 (1.06)
Knee Replacement	1391 (7.47)	15863 (6.97)	.019	17254 (7.01)
Major/moderate trauma, other	1020 (5.48)	10648 (4.68)	.036	11668 (4.74)
Other trauma	5534 (29.72)	66122 (29.05)	.015	71656 (29.10)
Other fracture	1294 (6.95)	15611 (6.86)	.004	16905 (6.87)
Vertebral fracture	344 (1.85)	4376 (1.92)	-.006	4720 (1.92)
Anxiety disorder	3823 (20.53)	42992 (18.89)	.041	46815 (19.01)
Substance use disorder or dependence: ethanol	222 (1.19)	2909 (1.28)	-.008	3131 (1.27)
Sleep problem, insomnia	3562 (19.13)	39486 (17.35)	.046	43048 (17.48)
Mood disorders, bipolar disorder or major depression	3726 (20.01)	40249 (17.68)	.060	43975 (17.86)
Mood disorders, other	731 (3.93)	7706 (3.39)	.029	8437 (3.43)
Other psychiatric conditions	1207 (6.48)	13078 (5.75)	.031	14285 (5.80)
Substance use disorder or dependence: other drug	925 (4.97)	7324 (3.22)	.088	8249 (3.35)
Diabetes (neuropathy)	2932 (15.74)	25568 (11.23)	.132	28500 (11.57)
**Other Medications**				
Second generation antipsychotics	587 (3.15)	5087 (2.23)	.057	5674 (2.30)
H2RA	1484 (7.97)	18043 (7.93)	.002	19527 (7.93)
Proton-pump inhibitors	7212 (38.73)	77612 (34.10)	.096	84824 (34.45)
Sucralfate	441 (2.37)	4267 (1.87)	.034	4708 (1.91)
Alzheimer medications	788 (4.23)	7157 (3.14)	.058	7945 (3.23)
Parkinson's medications	1088 (5.84)	12120 (5.32)	.023	13208 (5.36)
Anticonvulsants, Infrequent Use Outside of Seizure Disorders	351 (1.88)	3937 (1.73)	.012	4288 (1.74)
Antibiotics, Cephalosporins first, second generation	3254 (17.47)	38388 (16.87)	.016	41642 (16.91)
Antibiotics, Cephalosporins third generation	1461 (7.85)	15434 (6.78)	.041	16895 (6.86)
Antibiotics, Ciprofloxacin	2647 (14.21)	29242 (12.85)	.040	31889 (12.95)
Antibiotics, Other Fluoroquinolones	1860 (9.99)	18326 (8.05)	.068	20186 (8.20)

© 2025 Park EE et al. *JAMA Network Open*.

Antibiotics, Macrolides	3999 (21.47)	43336 (19.04)	.061	47335 (19.22)
Antimicrobials, Other	2032 (10.91)	22074 (9.70)	.040	24106 (9.79)
Antibiotics, Penicillins	4816 (25.86)	54754 (24.06)	.042	59570 (24.19)
Antibiotics, Sulfonomides	1715 (9.21)	20050 (8.81)	.014	21765 (8.84)
Antibiotics, Tetracyclines	1647 (8.84)	19675 (8.64)	.007	21322 (8.66)
Antibiotics, UTI	1125 (6.04)	12829 (5.64)	.017	13954 (5.67)
Antifungal agents--azoles	1033 (5.55)	11049 (4.85)	.031	12082 (4.91)
Antifungal agents--other	512 (2.75)	5274 (2.32)	.028	5786 (2.35)
Antiviral agents	2225 (11.95)	22888 (10.06)	.061	25113 (10.20)
Estrogens	674 (3.62)	7419 (3.26)	.020	8093 (3.29)
Ondansetron	2589 (13.90)	29871 (13.12)	.023	32460 (13.18)
Asthma treatment	1469 (7.89)	15006 (6.59)	.050	16475 (6.69)
Beta-agonists	3181 (17.08)	34459 (15.14)	.053	37640 (15.29)
Bronchodilators, other	1566 (8.41)	16135 (7.09)	.049	17701 (7.19)
Inhaled corticosteroids	4144 (22.25)	47347 (20.80)	.035	51491 (20.91)
Bisphosphonates	1332 (7.15)	13763 (6.05)	.045	15095 (6.13)
Other osteoporosis drugs	803 (4.31)	7151 (3.14)	.062	7954 (3.23)
**Continuous covariates**	**Median [IQR]**	**Median [IQR]**	**Standardized Difference**	**Overall**
Total days of long-acting opioid use	0 [0, 0]	0 [0, 0]	-0.129	0 [0 , 0]
Total days of short-acting opioid use	7 [0, 87]	2 [0, 30]	-0.228	3 [0 , 31]
Total days of methadone use	0 [0, 0]	0 [0, 0]	-0.046	0 [0 , 0]
Total days of buprenorphine use	0 [0, 0]	0 [0, 0]	-0.04	0 [0 , 0]
Daily short-acting opioid MME dose (average)	18.47 [0, 40]	10 [0, 31.45]	-0.13	11.41 [0 , 32.16]
Number of days since most recent home health visit	0 [0, 0]	0 [0, 0]	-0.056	0 [0 , 0]
Number of home health visits	0 [0, 0]	0 [0, 0]	-0.081	0 [0 , 0]
Number of days since most recent outpatient	7 [3, 21]	10 [4, 26]	0.132	9 [3 , 26]
Number of outpatient visits in the last year	19 [12, 30]	18 [11, 27]	-0.119	18 [11 , 28]
Number of days since most recent inpatient	0 [0, 0]	0 [0, 0]	-0.026	0 [0 , 0]
Number of inpatient days in the last year	0 [0, 0]	0 [0, 0]	-0.042	0 [0 , 0]
Number of days since most recent emergency department	0 [0, 28]	0 [0, 20]	-0.029	0 [0 , 21]
Number of emergency department visits in the last year	0 [0, 1]	0 [0, 1]	-0.004	0 [0 , 1]
Age on Day	72 [69, 78]	73 [69, 78]	0.039	73 [69 , 78]
Calendar year at start of follow-up	2016 [2015, 2017]	2016 [2016, 2017]	0.125	2016 [2016 , 2017]

[a]Other race: Asian/Pacific Islander, American Indian/Alaska Native, Hispanic, other, and unknown

© 2025 Park EE et al. *JAMA Network Open*.

eTable 6. Extended Table of Variables Used to Develop Propensity Score (IPT Weighted)

Variable	Pregabalin % Yes	Gabapentin % Yes	Standardized Difference	Overall % Yes
**Demographics**				
Male sex	33.15	33.29	-.003	33.22
Black (Or African-American)	7.52	7.19	. 013	7.36
Non-Hispanic White	79.82	79.89	-.002	12.79
Other race[a] (American Indian/Alaska Native, Asian/Pacific Islander, Hispanic, other, and unknown)	12.66	12.92	-.008	
Rural Zip Code	24.15	24.04	.002	24.1
**Chronic Pain Indications**				
Hospitalization	4.9	4.79	.005	4.84
Arthralgia	48.64	48.4	.005	48.52
Unspecified cause	18.22	17.69	.014	17.96
Autoimmune/other rheumatic diseases	3.57	3.56	.001	3.57
Back pain/degenerative back disorders	67.8	68.09	-.006	67.95
Fibromyalgia	14.7	14.07	.018	14.39
Headache, including migraine	14.58	14.39	.005	14.49
Inflammatory	22.42	22.02	.010	22.22
Neuropathic	60.72	60.83	-.002	60.77
Other musculoskeletal/soft tissue pain	71.91	71.57	.008	71.74
Other causes	27.79	27.79	0	27.79
Pain procedures	27.5	27.09	.009	27.29
**Pain Medications**				
Short-Acting Opioid Analgesics Use	52.92	52.04	.018	52.48
Analgesics, other	2.24	2.14	.007	2.19
Coxibs	5.36	5.24	.005	5.3
Cyclobenzaprine	5.89	5.77	.005	5.83
DMARDs	6.95	6.86	.003	6.9
Other skeletal muscle relaxants	10.26	10.11	.005	10.18
NSAIDs, non-selective	36.33	36.23	.002	36.28
Systemic oral corticosteroids	31	30.74	.005	30.87
Migraines, triptans	1.64	1.57	.006	1.61
Anticonvulsants, Primary Use Mood Stabilizer	1.26	1.23	.003	1.24
Anticonvulsants, Occasional Use Mood Stabilizer, Pain	1.7	1.58	.009	1.64
Bupropion	3.65	3.6	.003	3.63
Duloxetine	5.88	5.59	.012	5.73
Mirtazapine	1.94	1.89	.003	1.91
SNRI, Other	3.15	3.07	.004	3.11
SSRIs	19.94	19.79	.004	19.87
TCA/Heterocyclic	5.1	4.94	.007	5.02
Benzodiazepines	31.32	30.93	.008	31.13
Trazadone	6.58	6.51	.003	6.54
Sedatives other than benzos	9.98	9.78	.007	9.88
**Cardiovascular Diagnoses**				
Ill-defined or unspecified descriptions or complications of heart disease	5.49	5.26	.010	5.37
Cerebrovascular disease, late effects	1.08	1.05	.003	1.06
Cardiovascular Hospitalization	2.02	1.96	.004	1.99
Other chronic ischemic heart disease	20.88	20.55	.008	20.72
Arrhythmia: atrial fibrillation/flutter	8.56	8.68	-.004	8.62
Myocardial infarction	3.42	3.33	.005	3.37
Angina	3.99	3.9	.005	3.95

© 2025 Park EE et al. *JAMA Network Open*.

Arrhythmia, except atrial fibrillation	3.93	3.86	.004	3.9
Arrythmia, non-threatening (ICD10 only)	7.86	7.8	.002	7.83
Arterial embolism and thrombosis	3.05	3.15	-.005	3.1
CABG	3.41	3.28	.007	3.34
Cardiac symptoms	10.55	10.53	.001	10.54
Conduction disorder	4.19	4.09	.005	4.14
Diabetes (complications other than neuropathy)	12.21	12.04	.005	12.12
Diabetes (any, other than with complications)	34.94	34.5	.009	34.72
Venous disorders: phlebitis, thrombosis, varicosity with complications, lymphatics	8.71	8.54	.006	8.62
Hypertension, benign or unspecified	79.27	79.04	.006	79.16
Hypertension, malignant	1.18	1.17	.001	1.17
Hyperlipidemia	73.78	73.82	-.001	73.8
Hypotension	2.61	2.53	.005	2.57
Ischemic or unspecified stroke	9.94	9.77	.006	9.85
Obesity, morbid	8.43	8.32	.004	8.38
Obesity, not morbid or unspecified	18.45	18.22	.006	18.33
Other cerebrovascular disease	3.03	2.9	.008	2.96
Pacemaker	2.83	2.85	-.001	2.84
Peripheral vascular disease	17.66	17.57	.002	17.61
Percutaneous intervention	4.54	4.37	.008	4.45
Smoking and smoking-related disorders	19.47	18.98	.013	19.23
Syncope	4.35	4.2	.008	4.27
TIA	2.63	2.54	.006	2.58
Valve disease other than mitral valve	7.81	7.73	.003	7.77
Valve disease, mitral valve (including stenosis)	6.52	6.49	.001	6.51
**Cardiovascular Medications**				
Angiotensin converting enzyme inhibitors	29.69	29.69	0	29.69
Angiotensin receptor blocker	25.94	25.78	.004	25.86
Anticoagulants, NOACs	3.87	3.81	.003	3.84
Anticoagulants, Heparin, Low Molecular Weight Heparin, Other	4.44	4.38	.003	4.41
Anticoagulants, Vitamin K antagonists	3.89	3.89	0	3.89
Antihypertensives, Other	6.03	5.98	.002	6
Anti-arrhythmics	6.58	6.47	.004	6.52
Beta-blockers	35.02	34.79	.005	34.91
Calcium channel blockers	27.99	27.88	.002	27.93
Hypoglycemics, DPP4 Inhibitors	4.71	4.59	.006	4.65
Hypoglycemics, Thiazolidinediones	1.74	1.8	-.005	1.77
Hypoglycemics, Insulin	7.72	7.38	.013	7.55
Hypoglycemics, Metformin	19.35	19.23	.003	19.29
Hypoglycemics, SGLT2	1.17	1.18	0	1.18
Hypoglycemics, Sulfonylureas	9.4	9.18	.007	9.29
Diuretics, Potassium Sparing (and with hydrochlorothiazide)	5.95	5.91	.002	5.93
Lipid-lowering Drugs, Ezetimibe	3.02	2.91	.006	2.96
Lipid-lowering Drugs, Fibrates	4.42	4.43	-.001	4.43
Lipid-lowering Drugs, Other	1.84	1.81	.002	1.82
Lipid-lowering Drugs, Statins	55.79	55.73	.001	55.76
Diuretics, Loop	10.36	10	.012	10.18
Anti-anginals, nitrates	5.37	5.35	.001	5.36
Antiplatelet, P2Y12	7.49	7.35	.005	7.42
Antiplatelet, Other	1.33	1.26	.006	1.3
Diuretics, Thiazide	26.84	26.97	-.003	26.9
**Other Diagnoses and procedures**				
Asphyxia or hypoxemia	1.52	1.51	0	1.52
Asthma	10.25	9.97	.009	10.11

© 2025 Park EE et al. *JAMA Network Open*.

Chronic obstructive pulmonary disease	12.54	12.44	.003	12.49
Continuous positive airway pressure	7.15	7.17	-.001	7.16
Home oxygen	5.21	5.14	.003	5.18
Other respiratory diseases	6.56	6.34	.009	6.45
Pneumonia	3.35	3.26	.005	3.3
Sleep apnea	11.48	11.5	0	11.49
Other respiratory symptoms	34.39	33.89	.011	34.14
Other cognitive impairment	4.4	4.39	.001	4.39
Alzheimer and other dementias	4.16	4.06	.005	4.11
Other neurologic disorders	2.98	2.82	.009	2.9
Parkinsons and other movement disorders	8.78	8.55	.008	8.67
Other peripheral nervous system disorders	19.83	19.24	.015	19.54
Seizure disorders and convulsions	1.64	1.58	.005	1.61
Gastrointestinal bleeding, unspecified	2.26	2.24	.002	2.25
Gastrointestinal bleeding, lower	1.97	1.91	.004	1.94
Gallbladder/bile duct	2.82	2.78	.002	2.8
Constipation, irritable bowel syndrome	13.22	13.01	.006	13.11
Crohn disease/ulcerative colitis	1.07	1.02	.004	1.04
Liver, hepatitis	.96	1	-.004	0.98
Liver, other	5.24	5.35	-.005	5.3
Gastroparesis, intestinal obstruction	1.15	1.14	.002	1.14
Diverticulitis/diverticulosis without bleeding	11.21	11.04	.005	11.12
Esophagitis/gastritis/duodenitis without bleeding	9.9	9.89	0	9.9
Hemorrhoids without bleeding	6.39	6.31	.003	6.35
Peptic ulcer disease without bleeding	1.53	1.55	-.002	1.54
G.I. symptoms, abdominal pain	21.49	21.42	.002	21.46
G.I. symptoms, dyspepsia	4	4.09	-.004	4.04
G.I. symptoms, other lower	8.71	8.66	.002	8.68
G.I. symptoms, other upper	13.21	13.09	.004	13.15
G.I. symptoms, gastroesophageal reflux	30.04	29.8	.005	29.92
Debility, not specified	7.54	7.49	.002	7.52
Diabetic footwear	2.54	2.58	-.003	2.56
Dysphagia	4.1	4.08	.001	4.09
Malnutrition/abnormal weight loss/feeding problems	4.43	4.5	-.003	4.47
Limited mobility, cane or walker	3.65	3.51	.008	3.58
Limited mobility, wheelchair	1.09	1.02	.007	1.05
Osteoporosis	15.69	15.56	.004	15.63
Chronic skin ulcer	2.73	2.71	.001	2.72
Unintentional fall (not vigorous activity)	9.63	9.47	.006	9.55
Urinary incontinence	7	6.87	.005	6.94
Anemia, iron-deficiency	5.15	4.99	.007	5.07
Genitourinary bleeding	6.33	6.31	.001	6.32
Bleeding, unspecified site	2.76	2.69	.004	2.72
Coagulation defects and other hemorrhagic conditions	3.2	3.12	.005	3.16
Bleeding, other sites	1.97	1.87	.007	1.92
Other disorders blood, blood forming organs	4.27	4.18	.004	4.23
Acute renal failure	1.86	1.73	.010	1.79
Chronic renal failure, Stage I-IV/Unspecified	11.69	11.6	.003	11.64
Other renal disease	1.66	1.58	.006	1.62
Other kidney disorders	12.32	12.16	.005	12.24
Other serious metabolic disorders	5.6	5.55	.002	5.58
Adverse medication effect (not opioids or psychotropic drugs)	2.66	2.51	.009	2.59
History of cancer other than non-melanoma skin cancer	6.03	6.16	-.006	6.09

© 2025 Park EE et al. *JAMA Network Open*.

Skin cancer, not melanoma	5.98	5.94	.002	5.96
Infections, ENT	38.26	38.34	-.002	38.3
Other serious endocrine disorders	2.48	2.44	.002	2.46
Hypovolemia	2.69	2.64	.003	2.67
Disorders potassium/sodium balance	6.87	6.73	.006	6.8
Other fluid/electrolyte disorders	1.1	1.04	.005	1.07
Other hospitalization	1.06	1.05	.001	1.06
Adverse effect of medical device, graft, or procedure	4.35	4.23	.006	4.29
Other infectious and parasitic diseases, serious	9.78	9.64	.005	9.71
Infections, minor	25.37	24.89	.011	25.13
Urinary tract infections	18.53	18.16	.010	18.34
Neoplasms, not malignant or carcinoma in situ	30.6	30.76	-.003	30.68
Disorders of prostate	13.02	13.15	-.004	13.08
Swelling, mass, or lump: head, neck, chest, abdomen	2.54	2.59	-.003	2.56
Goiter/other thyroid disorders	7.64	7.73	-.003	7.69
Hyperthyroidism	1.59	1.55	.003	1.57
Hypothyroidism	28.48	28.43	.001	28.45
Prophylactic vaccination	67.2	67.56	-.008	67.38
Major/moderate trauma, head	1.45	1.44	.002	1.44
Hip replacement	3.31	3.41	-.005	3.36
Other joint replacement	1.09	1.06	.003	1.08
Knee Replacement	7.28	7.01	.010	7.14
Major/moderate trauma, other	4.83	4.74	.004	4.79
Other trauma	29.55	29.11	.010	29.33
Other fracture	6.9	6.87	.001	6.88
Vertebral fracture	2.03	1.92	.008	1.98
Anxiety disorder	19.33	19.02	.008	19.18
Substance use disorder or dependence: ethanol	1.34	1.27	.006	1.31
Sleep problem, insomnia	17.63	17.49	.004	17.56
Mood disorders, bipolar disorder or major depression	18.29	17.87	.011	18.08
Mood disorders, other	3.42	3.43	-.001	3.42
Other psychiatric conditions	5.68	5.8	-.005	5.74
Substance use disorder or dependence: other drug	3.57	3.36	.012	3.46
Diabetes (neuropathy)	11.91	11.58	.010	11.75
**Other Medications**				
Second generation antipsychotics	2.46	2.31	.010	2.39
H2RA	8.14	7.93	.008	8.04
Proton-pump inhibitors	34.45	34.45	0	34.45
Sucralfate	1.94	1.91	.002	1.93
Alzheimer medications	3.17	3.23	-.003	3.2
Parkinson's medications	5.49	5.36	.006	5.43
Anticonvulsants, Infrequent Use Outside of Seizure Disorders	1.7	1.74	-.003	1.72
Antibiotics, Cephalosporins first, second generation	17.08	16.91	.005	17
Antibiotics, Cephalosporins third generation	6.96	6.86	.004	6.91
Antibiotics, Ciprofloxacin	13.23	12.95	.008	13.09
Antibiotics, Other Fluoroquinolones	8.38	8.2	.006	8.29
Antibiotics, Macrolides	19.25	19.22	.001	19.24
Antimicrobials, Other	9.92	9.79	.004	9.86
Antibiotics, Penicillins	24.11	24.19	-.002	24.15
Antibiotics, Sulfonomides	8.92	8.84	.003	8.88
Antibiotics, Tetracyclines	8.72	8.66	.002	8.69

© 2025 Park EE et al. *JAMA Network Open*.

Antibiotics, UTI	5.8	5.67	.006	5.74
Antifungal agents--azoles	5.08	4.91	.008	4.99
Antifungal agents--other	2.46	2.35	.007	2.41
Antiviral agents	10.37	10.2	.006	10.29
Estrogens	3.34	3.29	.003	3.31
Ondansetron	13.37	13.18	.005	13.28
Asthma treatment	6.82	6.69	0.005	6.75
Beta-agonists	15.56	15.29	0.008	15.43
Bronchodilators, other	7.28	7.19	0.004	7.24
Inhaled corticosteroids	21	20.91	0.002	20.96
Bisphosphonates	6	6.13	-0.006	6.06
Other osteoporosis drugs	3.22	3.23	-0.001	3.22
**Continuous Variables**	**Pregabalin Median [IQR[**	**Gabapentin Median [IQR]**	**Standardized Difference**	**Overall**
Total days of long-acting opioid use	0 [0, 0]	0 [0, 0]	0.011	0 [0 , 0]
Total days of short-acting opioid use	3 [0, 37]	3 [0, 31]	0.023	3 [0 , 34]
Total days of methadone use	0 [0, 0]	0 [0, 0]	0.004	0 [0 , 0]
Total days of buprenorphine use	0 [0, 0]	0 [0, 0]	0.003	0 [0 , 0]
Daily short-acting opioid MME dose (average)	14.3 [0, 33.79]	11.4 [0, 32.16]	0.013	13.5 [0 , 33.16]
Number of days since most recent home health visit	0 [0, 0]	0 [0, 0]	0.007	0 [0 , 0]
Number of home health visits	0 [0, 0]	0 [0, 0]	0.011	0 [0 , 0]
Number of days since most recent outpatient	8 [3, 24]	10 [3, 26]	0.028	9 [3 , 25]
Number of outpatient visits in the last year	18[11, 28]	18 [11, 28]	0.015	18 [11 , 28]
Number of days since most recent inpatient	0 [0, 0]	0 [0, 0]	0.009	0 [0 , 0]
Number of inpatient days in the last year	0 [0, 0]	0 [0, 0]	0.012	0 [0 , 0]
Number of days since most recent emergency department	0 [0, 27]	0 [0, 21]	0.015	0 [0 , 23]
Number of emergency department visits in the last year	0 [0, 1]	0 [0, 1]	0.024	0 [0 , 1]
Age on Day	73 [69, 78]	73 [69, 78]	-0.001	73 [69 , 78]
Calendar year at start of follow-up	2016 [2015, 2017	2016 [2016, 2017	-0.013	2016 [2015 , 2017]

[a]Other race: Asian/Pacific Islander, American Indian/Alaska Native, Hispanic, other, and unknown

© 2025 Park EE et al. *JAMA Network Open*.

eTable 7. Negative Control for the Outcome (Hip Fracture)

	Pregabalin			Gabapentin			Hazard Ratio (95% CI)			
**Model**	**Person years**	**N events**	**Rate per 1000 years**	**Person years**	**N events**	**Rate per 1000**	**Unadjusted**		**Adjusted**	
No history of CHF	7993	41	5.13	120836	630	5.21	0.98	(0.72, 1.35)	1.02	(0.71, 1.46)
competing risk							0.98	(0.72, 1.34)	1.02	(0.71, 1.46)
**Race**										
Whites only	6044	35	5.79	99060	565	5.7	1.01	(0.72, 1.42)	1.06	(0.72, 1.55)
All other races	*									
**Gender**										
Female only	5177	29	5.6	78028	486	6.23	0.9	(0.62, 1.31)	0.96	(0.63, 1.48)
Male only	*									
**Diabetes**										
No diabetes	4110	23	5.6	70862	382	5.39	1.04	(0.68, 1.58)	1.16	(0.72, 1.85)
competing risk							1.04	(0.68, 1.58)	1.15	(0.72, 1.85)
diabetes	*									
competing risk							0.93	(0.58, 1.49)	0.82	(0.49, 1.37)
**CVD**										
No CVD	5381	21	3.9	84857	398	4.69	0.83	(0.54, 1.29)	1.06	(0.66, 1.73)
competing risk							0.83	(0.54, 1.29)	1.06	(0.65, 1.73)
CVD	2613	20	7.65	35978	232	6.45	1.18	(0.75, 1.87)	0.94	(0.57, 1.54)
competing risk							1.18	(0.75, 1.86)	0.93	(0.57, 1.54)

* Rows were censored for having very small numbers

© 2025 Park EE et al. *JAMA Network Open*.

eTable 8. E-Values

	Unadjusted			Adjusted		
**Analysis**	**Hazard Ratio**	**Lower, Upper**	**E-value**	**Hazard Ratio adjusted**	**Lower, Upper**	**E-value**
**Primary Analysis**	1.48	(1.23, 1.77)	2.3228523	1.48	(1.19, 1.77)	2.3228523
**Competing risk**	1.47	(1.23, 1.77)	2.301203946	1.45	(1.45, 1.77)	2.257774721
**History of Cardiovascular Disease**						
**No**	1.2	(0.93, 1.56)	1.689897949	1.21	(0.91, 1.60)	1.714083326
**Yes**	1.8	(1.39, 2.32)	3	1.85	(1.38, 2.47)	3.10399362
**Race**						
White	1.73	(1.42, 2.11)	2.853788236	1.65	(1.32, 2.05)	2.685615759
Non-White	0.72	(0.44, 1.18)	…	0.69	(0.39, 1.22)	…
**Sex**						
Female	1.63	(1.31, 2.03)	2.643360745	1.57	(1.23, 2.00)	2.515991543
Male	1.23	(0.88, 1.70)	1.761883446	1.27	(0.86, 1.89)	1.855576639
**Outpatient**	1.27	(1.04, 1.55)	1.855576639	1.27	(1.02, 1.58)	1.855576639
**Outpatient or Inpatient**	1.32	(1.12, 1.56)	1.969923072	1.34	(1.12, 1.60)	2.014981481
**All-cause mortality**	1.25	(0.98, 1.61)	1.809016994	1.26	(0.95, 1.76)	1.832363521

© 2025 Park EE et al. *JAMA Network Open*.

eTable 9. Proportional Assumption for Hazard Ratios

	rho	chi2	*df*	Prob>chi2
**Pregabalin**	0.01	0.15	1	0.7013
**Global test**		0.15	1	0.7013

eTable 10. Restricted Mean Survival Time

	Number of subjects	Restricted mean	Std. err.	[95% conf. interval]	Longest follow uptime
**Gabapentin**	227615	1419.117(*)	1.392355	[1416.39, 1421.85]	1458
**Pregabalin**	18622	1369.216(*)	7.201233	[1355.1, 1383.33]	1427
**Total**	246237	1417.919(*)	1.382393	[1415.21, 1420.63]	

eTable 11. Inverse Probability Weight Summary Statistics

	Gabapentin	Pregabalin
**IPTW Range**	[1.010, 2.364]	[1.652, 86.294]
**Effective Size**		
Unweighted	227615	18622
Weighted	246269094	244697723

eReferences

1. Rathore SS, Wang Y, Druss BG, Masoudi FA, Krumholz HM. Mental Disorders, Quality of Care, and Outcomes Among Older Patients Hospitalized With Heart Failure: An Analysis of the National Heart Failure Project. *Arch Gen Psychiatry*. 2008;65(12):1402. doi:10.1001/archpsyc.65.12.1402

2. So L, Evans D, Quan H. ICD-10 coding algorithms for defining comorbidities of acute myocardial infarction. *BMC Health Serv Res*. 2006;6(1):161. doi:10.1186/1472-6963-6-161

© 2025 Park EE et al. *JAMA Network Open*.
