# Supplementary material for: Initiation of Pregabalin vs Gabapentin and Development of Heart Failure
Source: JAMA Netw Open. 2025 Aug 1;8(8):e2524451. doi: 10.1001/jamanetworkopen.2025.24451 (PMC12317353; doi:10.1001/jamanetworkopen.2025.24451)
Supplement: Supplement 2. — Data Sharing Statement [file jamanetwopen-e2524451-s002.pdf]

Data Sharing Statement

Park. Initiation of Pregabalin vs Gabapentin and Development of Heart Failure. *JAMA Netw Open*. Published August 01, 2025. doi:10.1001/jamanetworkopen.2025.24451

Data
Data available: Yes
Data types: Other (please specify)
Additional Information: Data presented in this research cannot be shared publicly because of they are the property of the U.S. CMS Chronic Conditions Warehouse (CCW) and contain protected health information.
How to access data: Data are available from the CCW for researchers who meet the criteria for access to confidential data. Access to the CCW data is through the Research Data Assistance Center, www.resdac.org. Any programming codes and supporting documentation are available upon request.
When available: With publication

Supporting Documents
Document types: Statistical/analytic code
How to access documents: Data are available from the CCW for researchers who meet the criteria for access to confidential data. Access to the CCW data is through the Research Data Assistance Center, www.resdac.org. Any programming codes and supporting documentation are available upon request.
When available: With publication

Additional Information
Who can access the data: Researchers who meet the criteria for access to confidential data
Types of analyses: For any purpose
Mechanisms of data availability: After approval through the Research Data Assistance Center, www.resdac.org
